# How Do the Indices based on the EAT-Lancet Recommendations Measure Adherence to Healthy and Sustainable Diets? A Comparison of Measurement Performance in Adults from a French National Survey

**DOI:** 10.1016/j.cdnut.2025.104565

**Published:** 2025-02-07

**Authors:** Agustín R Miranda, Florent Vieux, Matthieu Maillot, Eric O Verger

**Affiliations:** 1 MoISA, University of Montpellier, CIHEAM-IAMM, CIRAD, INRAE, Institut Agro, IRD, Montpellier, France; 2 MS-Nutrition, Marseille, France

**Keywords:** sustainable diet, planetary boundaries, healthy diet, dietary index, dietary assessment, food systems, validity, nutrient adequacy

## Abstract

**Background:**

Measuring adherence to EAT-Lancet recommendations for healthy and sustainable diets is challenging, leading to diverse methods and a lack of consensus on standardized metrics. Available indices vary mainly in scoring systems, food components, units, energy adjustments, and cut-off points.

**Objectives:**

To evaluate and compare the measurement performance of 9 dietary indices for assessing adherence to EAT-Lancet reference diet.

**Methods:**

This cross-sectional study utilized repeated 24-h dietary recall data from 1723 adults in the French Third Individual and National Study on Food Consumption Survey (INCA3, 2014–2015). Sociodemographic, nutritional, and environmental variables were analyzed to assess the validity and reliability of dietary indices.

**Results:**

The 4 indices assessing their food components with proportional scoring captured dietary variability, were less dependent on energy intake and converged to a large extent with nutritional indicators. Although the 3 binary indices showed a stronger correlation with environmental indicators, 1 proportional index converged with both domains. Indices had valid unidimensional structures, meaning that the combination of food components within each index accurately reflected the same construct, supporting the use of total scores. Furthermore, the indices differed between sociodemographic groups, demonstrating concurrent-criterion validity. Higher scores were associated with higher nutritional quality and lower environmental impact, but with unfavorable results for zinc intake, vitamin B12, and water use. A low concordance rate (32%–43%) indicated that indices categorized individuals differently.

**Conclusions:**

Researchers must align study objectives with the applicability, assumptions, and significance of chosen indices. Indices using proportional scoring allow a global understanding of dietary health and sustainability, being advantageous in precision-focused research (for example, clinical trials or epidemiological research). Conversely, indices based on binary scoring offer a simplified perspective, serving as valuable tools for surveys, observational studies, and public health. Recognizing their strengths and limitations is crucial for a comprehensive assessment of diets and their implications.

## Introduction

Currently, global policy agenda emphasizes nutritional strategies focused on supplying vital nutrients, reducing environmental impact, and advancing long-term sustainability [1]. Organizations such as the FAO and the WHO call for healthy and sustainable food patterns that are accessible, affordable, safe, equitable, and culturally acceptable [2]. This issue aligns with the 17 United Nations’ Sustainable Development Goals (SDGs) targeting hunger eradication, improved nutrition, and food system sustainability [3]. However, meeting the goals of operating within planetary boundaries and promoting healthy diets remains a challenge. Planetary boundaries are essential environmental limits that must be respected to maintain the global balance and overall functioning of the Earth system, but current food systems, particularly food production practices, threaten these limits significantly [4]. Food systems include all the aspects of feeding and nourishing people, from production to consumption, involving multiple resources and their impact on nutrition, health, economy, society, and environment [5]. Food systems contribute significantly to climate change with 34% of greenhouse gas emissions (GHGE) [6] and 70% of freshwater consumption, leading to resource depletion, and contribute to pollution, land use, and biodiversity loss [7].

To advance the achievement of the SDGs and Paris Climate Agreement commitments, the EAT-Lancet Commission introduced a planetary health diet in 2019 as a global standard for adults [7]. This reference diet, based on 2500 kcal per day for a 70-kg 30-year-old man or 60-kg 30-year-old woman with moderate-to-high physical activity, sets ranges for specific food groups to promote healthy eating and sustainable food production (Supplemental Table 1). In this regard, the planetary health diet prioritizes the consumption of vegetables, fruits, legumes, whole grains, nuts, and fish, although limiting the intake of red meat and tubers. It also promotes moderate consumption of eggs, poultry, and dairy products [7]. Although this diet can serve as benchmarks, criticisms include impracticality for poor settings, and its adult-focused targets may not apply directly to vulnerable groups [8]. Despite its limitations, the EAT-Lancet initiative offers an innovative framework for sustainability, fostering consensus and stimulating discussions among academics, community organizations, and policymakers [9,10]. It has encouraged a re-evaluation of food systems in the context of climate change, prompting governments to closely examine these systems, inspire sustainable policies, and conduct a systemic analysis of potential trade-offs and benefits [9]. Given its impact, the EAT-Lancet report remains a critical tool that needs for driving transformative change in global diets and food systems.

Upon the release of the EAT-Lancet report, measuring adherence to the planetary health diet faced challenges, leading to diverse methods without consensus in their development [11]. Early instruments assess adherence using binary scoring (that is, for each food component within the index, a score of 1 is assigned when meeting the recommendation and 0 for not meeting). Among these are the EAT-Lancet Diet Score (ELDS), the Healthy and Sustainable Diet Index (HSDI), and the Dietary Index (DI), based on data from the United Kingdom, Mexico, and Germany, respectively [[12], [13], [14]]. Although they are associated with health and environmental outcomes, the validity of these indices remains to be explored. Recent indices have refined their designs by incorporating proportional scoring, adjustments for energy intake, and interchangeability between food components. Among these, the World Index for Sustainability and Health (WISH) was developed using data from Vietnam and is positively associated with health indicators [15]. The Planetary Health Diet Index (PHDI) and the Healthy Reference Diet (HRD) are based on data from 2 large cohorts, respectively, from Brazil and the Netherlands and are associated with cardiovascular, nutritional, and environmental indicators [[16], [17], [18], [19]]. Likewise, the EAT-Lancet Diet Index (ELD-I), developed with data from France, shows a positive correlation with nutritional quality and a negative correlation with environmental impact [20]. Finally, the EAT-Lancet Index (ELI), developed with Swedish data, shows associations with reduced mortality and a lower risk of chronic diseases [21,22], whereas the Sustainable and Healthy Diet Index (SHDI), derived from the Gambian Integrated Household Survey, lacks documented associations with nutritional and environmental indicators [23]. Despite notable progress in the development of these indices, there are still gaps on their validity, and they have not yet been comprehensively compared using data from the same sample.

Although efforts have been made to develop methods for measuring adherence to EAT-Lancet diet, further research is needed for the assessment of various measurement properties to ensure their validity and reliability [24,25]. Some indices like PHDI and ELD-I have undergone validation, but most have not reported their validity indicators. This is crucial because many indices may lack representativity due to study design, participant characteristics, or sample size. Dietary indices, particularly those based on EAT-Lancet diet, require validation using nutritional and environmental indicators. When selecting a dietary index, aligning it with research goals, comprehending the scoring system, and ensuring a robust and unbiased validation process are crucial to enhance reliability in nutritional epidemiology [26].

In this context, the aim of this study was to assess and compare the measurement performance, focusing on the aspects of validity and reliability, of 9 dietary indices representing the EAT-Lancet reference diet using a national representative sample from France.

## Materials and Methods

### Study population and design

Data were extracted from the French Third Individual and National Study on Food Consumption Survey (INCA3). The INCA3 is a nationally representative cross-sectional survey conducted on 4114 individuals in mainland France between February 2014 and September 2015. The methodology and study design of this survey are described in detail elsewhere [27]. In this study, participants aged ≥18 y old were included and mis-reporters (that is, participants who under- or over-report their food intake) were excluded, using the basal metabolic rate estimated using the Henry equation and the cut-off values proposed by Black [28,29]. Thus, the final sample contained 1723 adults (723 men and 1000 women). The flowchart of study participants is detailed in Supplemental Figure 1.

The INCA3 study was carried out in accordance with the Declaration of Helsinki and received approval from the French Data Protection Authority (Decision DR 2013–228) on May 2, 2013, following a favorable opinion from the Advisory Committee on Information Processing in Health Research on January 30, 2013 (Opinion 13.055). Verbal informed consent was obtained from all participants before their voluntary inclusion in the study. Verbal consent was witnessed and formally recorded.

### Dietary data

Dietary intake was assessed through 3 nonconsecutive dietary recalls, 2 weekdays and 1 weekend, over a 3-wk period. During the 3 selected days, individuals were asked to describe their food consumption by identifying all foods and beverages consumed during the day or at night. They were asked to describe these foods in as much detail as possible (for example, brand, cooking method, preservation method, and sugar/fat/salt content) and to quantify them using a picture book of food portions sizes and household measurements specifically validated for the INCA3 study. Data were collected by telephone by trained interviewers using the standardized 24-h recall program GloboDiet developed by the International Agency for Research on Cancer [27]. To account for variations in food intake across different seasons and days of the week, all seasons and days were included in the study design.

During the initial home visit, participants were provided with the necessary tools for the dietary interviews, including the picture book, and were thoroughly instructed on its use. Next, participants were contacted by telephone on 3 occasions to conduct the dietary interviews. A computerized algorithm was employed to ensure the capture of 2 weekdays and 1 weekend day. In cases where participants did not have the picture book available during the call, a follow-up appointment was scheduled later the same day to complete the interview. The interview days were not disclosed to the participants to prevent them from predicting and adjusting their food intake.

Energy and nutrient contents of foods were based on the 2016 database from the French Centre d'Information sur la Qualité des Aliments [30]. The traditional recipes or dishes containing various foods were disaggregated into their ingredients based on mean recipes obtained from an existing recipe database, and on recipes sourced from the most popular cooking website in France (that is, marmiton.org) [31]. Dietary intakes were calculated by calculating the mean of the data from the 3 24-h dietary recalls. The dietary data were used to calculate EAT-Lancet adherence indices, nutritional quality and environmental scores for each individual from the INCA3.

### Estimation of indices of adherence to the EAT-Lancet Diet

The indices were selected due to their relevance and the differences in their characteristics, such as the metric, scoring systems, and cut-off points, which allow for a differentiated operationalization of the EAT-Lancet diet [11,32,33]. TABLE 1, TABLE 2 display the correspondences between the food components and the reference values for the 9 indices based on EAT-Lancet recommendations. Supplemental Material lists the food items, scoring criteria, and cut-offs points that were considered in each food component (Supplemental Tables 2–11).

**TABLE 1:** Equivalences between the food components of the proportional indices based on the EAT-Lancet recommendations and their scoring standards.

Proportional scoring								
WISH^1^ from 0 to 130 13 food components		PHDI^2^ from 0 to 150 16 food components		ELD-I^1^ from –∞ to +∞ 14 food components		HRD^1^^,^^3^ from 0 to 140 14 food components		
Whole grains	≥125 (100–150)	Whole cereals	≥32.4	Whole grains	≤464	Whole grains	≥464	≥372
—		Tubers and potatoes	1.6 (0–3.1)	Potatoes and tuber	≤100	Tubers or starchy vegetables	50 (0–150)	40 (0–120)
Vegetables	300 (200–600)	Vegetables	≥3.1	Vegetables	≥200	Vegetables	≥300	≥240
Fruits	200 (100–300)	Fruits	≥5.0	Fruits	≥100	Fruits	≥200	≥160
Dairy foods	250 (0–500)	Dairy	6.1 (0–12.2)	Dairy foods	≤500	Dairy foods	250 (0–750)	200 (0–600)
Red meat	14 (0–28)	Red meat	0 (0–2.4)	Beef, lamb, pork	≤28	Beef, lamb and pork	≤14	≤12
Chicken and other poultry	29 (0–58)	Chicken and substitutes	0 (0–5.0)	Chicken and poultry	≤58	Chicken and other poultry	29 (0–88)	23 (0–69)
Eggs	13 (0–25)	Eggs	0.8 (0–1.5)	Eggs	≤25	Eggs	13 (0–38)	10 (0–30)
Fish	28 (0–100)	Fish and seafood	1.6 (0–5.7)	Fish	≤100	Fish	28 (0–128)	22 (0–102)
Legumes	75 (0–100)	Legumes	≥11.3	Legumes	≤100	Dry beans, lentils, and peas	≥50	≥40
Nuts	50 (0–75)	Nuts and peanuts	≥11.6	Nuts	≥25	Nuts	50 (0–150)	40 (0–120)
Unsaturated oils	40 (20–80)	Vegetable oils	16.5 (0–30.7)	Unsaturated oils	≤80	Added fats (Unsaturated/Saturated fats ratio)	≥13	≥11
Saturated oils	11.8 (0–11.8)	Animal fats	0 (0–1.4)	Saturated oils	≤11.8			
Added sugars	31 (0–31)	Added sugars	0 (0–4.8)	Added sugars	≤31	Added sugars	≤31	≤25
—		DGV/total ratio	29 (0–100)	—		—		
—		ReV/total ratio	38.5 (0–100)	—		—		
—		—		—		Soy foods	≥25	≥20

Abbreviations: DGV/total ratio, ratio between the energy intake of dark green vegetables and the total of vegetables; ELD-I, EAT-Lancet Diet Index; HRD, Healthy Reference Diet; PHDI, Planetary Health Diet Index; ReV/total ratio, ratio between the energy intake of red and orange vegetables and the total of vegetables; WISH, World Index for Sustainability and Health.

The reference intervals, applicable to certain indexes in their calculations, are presented within parentheses.

1 Values expressed as g/d.

2 Values expressed as caloric percentage from the reference diet proposed by the EAT-Lancet Commission.

3 Standardized daily at 2500 kcal/day for men (first column) and 2000 kcal/day for women (second column).

**TABLE 2:** Equivalences between the food components of the graded and binary indices based on the EAT-Lancet recommendations and their scoring standards.

Graded scoring				Binary scoring					
ELI^1^ from 0 to 42 14 food components		SHDI^1^ from 0 to 48 16 food components		HSDI^2^ from 0 to 13 13 food components		ELDS^1^ from 0 to 14 14 food components		DI^1^ from 0 to 18 18 food components	
Whole grains	232	Whole grains	>116	Whole grains	≥32.44	Whole grains	≤464	Whole grains and all grains	≤464
Potatoes	50 (0–100)	Potatoes and cassava	>50	Tubers or starchy vegetables	≤1.56	Tubers and starchy vegetables	≤100	Tubers or starchy vegetables	≤100
Vegetables	300 (200–600)	All vegetables	>300	Vegetables	≥3.12	Vegetables	≥200	Vegetables	≥200-≤600
Fruits	200 (100–300)	All fruits	>200	Fruits	≥5.02	Fruits	≥100	Fruits	≥100-≤300
Dairy	250 (0–500)	Dairy	250–500	Milk and dairy	≤6.12	Dairy foods	≤500	Dairy foods	≤500
Beef and lamb	7 (0–14)	Beef and lamb	7–14	Beef and pork	≤0.64	Beef, lamb, pork	≤28	Beef and lamb	≤14
Pork	7 (0–14)	Pork	7–14					Pork	≤14
Poultry	29 (0–58)	Poultry	29–58	Chicken and other poultry	≤2.48	Chicken, other poultry	≤58	Chicken and other poultry	≤58
Eggs	13 (0–25)	Eggs	13–25	Eggs	≤1.00	Eggs	≤25	Eggs	≤25
Fish	28 (0–100)	Fish	>28	Fish and seafood	≤1.60	Fish	≤100	Fish	≤100
Legumes	75 (0–150)	Beans, lentils and peas	>75	Legumes, soybeans and tree nuts	≥23.0	Dry beans, lentils, peas	≤100	Dry beans, lentils, peas	≤100
Nuts	50 (0–100)	Peanuts and tree nuts	>50			Peanuts or tree nuts	≥25	All nuts	≥25
Unsaturated oils	40 (20–80)	Unsaturated oils	>40	Unsaturated fats	≥14.16	Added fats	0.8	Unsaturated oils	≥20-≤80
				Saturated fats	≤3.84			—	
Added sugar	31 (0–31)	Added sugar	7.75–31	Added sugars	≤5.00	Added sugar	≤31	All sweeteners	≤31
—		—		—		Soy foods	≤50	Soy foods	≤50
—		—		—		—		Lard or tallow	≤5
—		—		—		—		Butter	0
—		Refined grains	<116	—		—		—	
—		Palm oil	<1.7	—		—		Palm oil	<6.8

Abbreviations. DI, Dietary Index; ELDS, EAT-Lancet Diet Score; ELI, EAT-Lancet Index; HSDI, Healthy and Sustainable Diet Index; SHDI, Sustainable and Healthy Diet Index.

The reference intervals, applicable to certain indexes in their calculations, are presented within parentheses.

1 Balues expressed as g/d.

2 Values expressed as caloric percentage from the reference diet proposed by the EAT-Lancet Commission.

#### World Index for Sustainability and Health

The WISH is an index based on a proportional scoring that includes 13 food components [15]. Each food component is scored on a scale ranging from 0 (noncompliance) to 10 (full compliance) points, using reference values in grams based on both the healthiness and environmental sustainability of the food component. Subsequently, the scores for the food components are summed to calculate the total score, which ranges from 0 to 130 (the higher, the greater adherence to a healthy and sustainable diet). Concerning saturated fats and added sugars, both components are scored using a binary scoring: 10 points if consumption is equal to or below the recommended intake and 0 points if it exceeds it. More information about the WISH is available elsewhere [15].

#### Planetary Health Diet Index

The PHDI is an index comprised of 16 food components that are scored using a proportional system based on reference values expressed as ratios of energy intake [16]. Energy intake ratios are defined as the sum of calories from all foods in a food component divided by the total calories from all foods in the PHDI index (that is, energy within a food component in the numerator, whereas the sum of the energy of all foods included in the PHDI in the denominator). Each food component was categorized either in an adequacy component (nuts and peanuts, fruits, legumes, vegetables, and whole grain cereals), optimum component (eggs, dairy products, fish and seafood, tubers and potatoes, and vegetable oils), moderation component (red meat, chickens and substitutes, animal fats, and added sugars) or ratio component (dark green vegetables/total vegetables and red–orange vegetables/total vegetables). Adequacy, moderation, and optimum components could score a maximum of 10 points, whereas the ratio ones could score a maximum of 5 points. The sum of these components results in a total score that ranges from 0 to 150 points. Higher scores indicate greater adherence to the EAT-Lancet diet. More information is available elsewhere [16].

#### EAT-Lancet Diet Index

The ELD-I index assesses the proximity of a diet to the EAT-Lancet reference for 14 food components using a proportional scoring [20]. ELD-I calculations are adjusted by individual energy intake, with a reference of 2500 kcal. Additionally, this index distinguishes between different food components, awarding positive scores when the consumption of recommended foods exceeds the reference and when the consumption of foods to limit is below it. Conversely, it assigns negative scores when the consumption of recommended foods is below the reference and when the consumption of foods to limit exceeds the reference. As a result, ELD-I calculation yields a continuous unbounded score that can be either positive or negative. Higher scores reflect a greater alignment with the EAT-Lancet recommendations. More information about the development of ELD-I index is available elsewhere [20].

#### Healthy Reference Diet

The HRD index uses a proportional scoring system to assess the alignment of a diet with the EAT-Lancet reference across 14 food components [19]. It incorporates sex-specific energy adjustments for cut-off criteria, with the thresholds for men set at 2500 kcal/d and for women at 2000 kcal/d. Participants received proportional scores ranging from 0 to 10 for each recommendation, with the total score spanning from 0 (indicating no adherence) to 140 (indicating complete adherence). The HRD score categorizes food groups into 4 components: adequacy (for example, whole grains, vegetables, and fruits), moderation (for example, added sugars and beef, lamb, and pork), optimum (for example, tubers and starchy vegetables, dairy foods, and eggs), and ratio (for example, added fats), based on their established associations with chronic diseases, as supported by relevant research. Further details regarding can be found elsewhere [19].

#### EAT-Lancet Index

The ELI index consists of 14 food components divided into 2 groups: 7 positive components or “emphasized foods” and 7 negative components or “limited foods” [21]. The alignment of dietary intake in grams per day (without adjustment on daily energy) with the EAT-Lancet recommendations is assessed using a scoring system based on a graded scale ranging from 0 (noncompliance) to 3 points (high compliance). As a result, the total score of the ELI DI ranges from 0 to 42 points. More information about the ELI index, scoring criteria, and cut-offs points is available elsewhere [21].

#### Sustainable and Healthy Diet Index

The SHDI evaluates the adherence to the EAT-Lancet recommendations through a graded scoring system based on the consumption levels of 16 food groups [23]. Higher scores are awarded for beneficial foods (for example, vegetables, fruits, and whole grains), whereas points are deducted for harmful foods (for example, beef, pork, potatoes, and added sugars). The SHDI includes cut-off scores tailored to micronutrient adequacy and uses a 0–3-point system to assess food group intakes. The final SHDI score, ranging from 0 to 48, reflects the degree of adherence to the EAT-Lancet diet, with higher scores indicating better compliance. Further details on the SHDI index, scoring criteria, and cut-off points are available elsewhere [23].

#### Healthy and Sustainable Diet Index

The HSDI assesses the degree of compliance with EAT-Lancet recommendations through a binary scoring [13]. This index analyses the percentage of energy intake from 13 food components (based on a daily energy of 2500 kcal), assigning one point when the recommended energy percentage is met and zero points otherwise. The food components are then summed, resulting in a scale ranging from 0 to 13 points, where a higher score reflects greater adherence to EAT-Lancet recommendations. More information about the HSDI index is available elsewhere [13].

#### EAT-Lancet Diet Score

The ELDS index consists of 14 food components and relies on a binary scoring [12]. One point is assigned to each component for meeting each of the recommended intakes in terms of grams per day without energy adjustment. The sum of all components results in a total score that ranges from 0 to 14 points. Higher scores indicate a greater level of adherence to the EAT-Lancet recommendations. More information about the development of ELDS index, scoring criteria, and cut-offs points is available elsewhere [12].

#### Dietary Index

The DI index comprises 18 food components to assess adherence to the EAT-Lancet diet and uses a binary scoring system [14]. First, each food component was standardized to a 2500-kcal/day reference by multiplying the food component’s intake by 2500 kcal and dividing the product by the individual’s energy intake, in alignment with the EAT-Lancet reference diet. Participants received 1 point for food component intakes that met the recommendations, and 0 points for those that did not, resulting in DI scores ranging from 0 (low adherence) to 18 (high adherence). Further information on DI index is available elsewhere [14].

### Nutritional quality assessments

#### Assessment of nutrient adequacy

The PANDiet quality index, used for evaluating nutritional adequacy [34], is a comprehensive measure assessing the probability of meeting recommended intake levels for 33 nutrients. Comprising 2 subscores—“adequacy” and “moderation”—the former involves calculating the mean of probabilities for 27 nutrients, whereas the latter computes the meanprobabilities for 6 nutrients that should be kept within upper limits. Nutrients for adequacy subscore include proteins, total carbohydrates, dietary fiber, total fats, 4 essential fatty acids, 11 vitamins, and 10 minerals. In contrast, moderation subscore focuses on limiting intake of proteins, total carbohydrates, total sugars, total fats, saturated fatty acids, and sodium. Each subscore undergoes multiplication by 100, followed by the calculation of their mean. This process yields the total PANDiet score, which ranges from 0 to 100. The PANDiet metrics are expressed as likelihood of adequacy, with higher scores implying superior diet quality and greater nutrient adequacy. The dietary reference values used to calculate PANDiet were based on guidelines issued by the French Agency for Food, Environmental and Occupational Health and Safety (see Supplemental Table 12).

#### Global Diet Quality Score

The Global Diet Quality Score (GDQS) is a recently developed score designed to assess diet quality based on 25 food categories considered critical for nutrient supply and chronic disease risk prevention [35]. This score is composed of 16 healthy food components (higher intake results in a higher score), 7 unhealthy food components (higher intake results in a lower score), and 2 food components considered unhealthy when consumed in excess (resulting in a score of 0 for both insufficient and excessive intake). The total GDQS score is calculated by adding up the points assigned to the 25 food components and ranges from 0 to 49 points. It is further divided down into 2 subscores: GDQS+, which reflects the sum of the healthy food components with a range between 0 and 32, and GDQS–, which is based on the unhealthy or overconsumed food components, with a score ranging from 0 to 17 (Supplemental Table 13).

#### Comprehensive Diet Quality Index

The Comprehensive Diet Quality Index (cDQI) discriminates between the intake of plant and animal food components considered beneficial or detrimental to health [36]. The cDQI is composed of 11 components of plant-based foods (pDQI) and 6 components of animal-based foods (aDQI). Healthy foods receive positive scores, in contrast to unhealthy foods that receive negative scores. The total cDQI score ranges from 0 to 85 (Supplemental Table 14).

#### Dietary Inflammatory Index

The Dietary Inflammatory Index (DII) was used to assess the inflammatory potential of the diet [37]. The DII was developed following an extensive literature review that identified 45 dietary parameters (foods or nutrients) associated with 6 inflammatory biomarkers: IL-1β, IL-4, IL-6, IL-10, TNF-α, and C-reactive protein. In this study, a total of 34 out of the possible dietary parameters were used to calculate the DII. The computation includes comparing dietary intake with the global standard, calculating Z-scores, converting them to centered proportions, multiplying by inflammatory effect scores for each parameter, and summing to get the total DII score (that is, a higher DII indicates a more proinflammatory diet) [38]. The specific steps for the calculation are available in Supplemental Material (Supplemental Table 15).

#### Composite Dietary Antioxidant Index

The Composite Dietary Antioxidant Index (CDAI) was calculated with the aim of assessing the overall exposure to dietary antioxidants [39]. The CDAI is a score that integrates various dietary antioxidants, including vitamins A, C, and E, manganese, selenium, and zinc, and it reflects an individual’s dietary antioxidant profile. To obtain the CDAI, each of these 6 dietary antioxidants was standardized by subtracting the sex-specific mean and dividing by the sex-specific standard deviation, and then, they were summed up (Supplemental Material, page 26). The higher the CDAI, the greater the bioavailability of antioxidants in the diet, suggesting a potentially higher level of defense against oxidative stress and health protection [40].

### Environmental data

The analysis of environmental impact was carried out using the Agribalyse 3.1.1 database, which was developed by the French Agency for the Environment and Energy Management [41]. Agribalyse 3.1.1 provides reference data on the environmental impacts of agricultural and food products through a database constructed using the Life Cycle Assessment methodology, considering different stages of the food chain. In this study, the aggregated score product environmental footprint (PEF) and the following 14 metrics were used: GHGE (kg carbon dioxide eq), exposure ionizing radiation (kg U235 eq), photochemical ozone formation (kg NMVOC eq), ozone depletion (Freon-11), emission of particulate matter in change (mortality due to particulate matter emissions), acidification (mol H+ eq), terrestrial eutrophication (mol N eq), freshwater eutrophication (kg P eq), marine eutrophication (kg N eq), freshwater ecotoxicity in (CTUe), water use (m^3^ world eq), land use (loss of soil organic matter content in kg carbon deficit), fossils resource use (MJ), and metals and minerals resource use (kg Sb eq). The complete methodology of Agribalyse 3.1.1 is explained elsewhere [42].

### Other data

Sociodemographic variables included sex (woman or man), age group (18–44 y, 45–64 y, or 65–79 y), educational level (primary and middle school, high school, 1 to 3 y of post-secondary education, or ≥4 y of post-secondary education), income per consumption unit (< €900/month/CU, €900–€1340/month/CU, €1340–€1850/month/CU, or ≥ €1850/month/CU), weight status according to WHO body mass index categories (underweight, normal, overweight, obesity, and morbid obesity), smoking habit (smoker or nonsmoker), and level of physical activity assessed by an adapted version of the Recent Physical Activity Questionnaire (categorized as low, moderate, or high) [27,43]. Additionally, the ratio between PANDiet score and PEF was calculated to combine both nutritional adequacy and global environmental indicator into a single indicator, which was then analyzed for trends.

### Statistical analysis

Statistical analyses were performed using Stata (version 18, StataCorp), and the threshold for statistical significance was *P* < 0.05. The weighting factors supplied within the INCA3 survey were employed in the analyses to address the complex survey design and to guarantee national representativeness [27]. Mean and standard errors (SEs) were calculated for numerical variables, and percentages for categorical ones. Q–Q plot and Kernel density histograms were used to assess the distribution of the indices against normal distribution. Correlation coefficients were interpreted following established guidelines, where coefficients below 0.2, between 0.2 and 0.39, 0.4 and 0.59, 0.6 and 0.79, and 0.8 to 1 indicate very weak, weak, moderate, strong, and very strong associations, respectively [44]. Table 3 summarizes the strategies used to evaluate measurement performance of EAT-Lancet indices.

**TABLE 3:** Strategies used to evaluate measurement performance of EAT-Lancet indices.

	Definition	Question	Method
Reliability	Reliability encompasses the homogeneity of food components, which reflects how each food component interacts and aligns with others. Internal consistency reliability assesses the uniformity of the construct being measured, evaluating the correlation among components and ensuring the index's stability, consistency, and accuracy.	To what extent do the food components consistently measure the same construct? Are all food components aligned with the overall construct the index is designed to measure? Does each food component effectively contribute to the index?	Homogeneity was assessed by calculating the correlations between food components as well as the correlation of each food component with the total score. Internal consistency reliability was evaluated using Guttman’s lambda coefficients, which include 6 measures derived from the analysis of the total variance of the index, the variance of each component, and the covariance between them.
Structural validity	It evaluates whether the food groups in an index are appropriately organized and effectively represent the intended underlying dimensions.	What is the underlying structure of the index? To what extent does the dietary index demonstrate unidimensionality, making it suitable for use as a total score?	Structural equation modeling was conducted to evaluate the unidimensionality of the indices, and goodness-of-fit indices were computed to assess the model's overall fit.
Index variability	It refers to the extent to which an index captures a broad range of scores across individuals in a population. A sufficient level of variability ensures that the index can effectively differentiate between individuals or groups with varying levels of adherence.	Does the index allow for sufficient variation in scores among individuals?	The percentile distribution, spanning from the first to the 99th percentile, was analyzed to assess the range and variability of the index scores.
Relationship with total energy intake	It refers to the extent to which an index measures diet quality independently of the overall quantity of energy consumed. Evaluating this relationship ensures that the index is not confounded by caloric intake.	Is the index capable of measuring diet quality independently of energy?	Pearson’s correlation analysis was conducted to investigate the association between the indices and total energy intake.
Interindex concordance	It refers to the degree of agreement between 2 or more indices that aim to measure similar constructs. It evaluates how closely the indices classify individuals into the same or similar categories, such as quantiles or adherence levels.	To what extent do the indices agree in classifying individuals into quantiles?	The proportion of participants classified into the same quantile, adjacent quantile, and opposite extreme quantile was analyzed. Additionally, Fleiss’ kappa coefficients were calculated, with a value of 1 indicating perfect agreement and values closer to 0 reflecting poor concordance between the indices.
Concurrent-criterion validity	It evaluates how well a dietary index correlates with external criteria, for example, in distinguishing groups with known differences in diet quality.	Does the index distinguish between groups known to have differences in diet quality?	It was assessed by comparing the indices across various sociodemographic groups with known differences in dietary patterns. Models of analysis of covariance were used for comparisons.
Convergent validity	It assesses the degree to which a dietary index correlates with other established measures of similar dietary constructs.	Does the index correlate with other indicators that measure similar constructs (for example, nutritional health, environmental impact)?	Given that indices based on the EAT-Lancet have been designed to assess healthy and sustainable diets, their convergent validity was evaluated by analyzing Spearman's correlations with established nutritional and environmental indicators.
			Additionally, differences, effect sizes, and trends in nutrient adequacy and environmental impact across the index quantiles were assessed to examine how dietary patterns and sustainability outcomes vary.

#### Assessment of reliability and structural validity of indices

To assess homogeneity of food components within each index, correlations between them (that is, intercomponent correlations) were calculated using different methods according to the data to test how each food component behaves individually with respect to the others. Pearson’s correlation was used for proportional indices, polychoric correlation for those using a graded scale, and tetrachoric correlation for dichotomous data. Also, Pearson’s correlation and point-biserial correlation were used to calculate the relationships between each component and the total score (that is, component-total correlations), with values above 0.80 considered as redundant.

Internal consistency reliability is a measure of the extent to which the food components in a DI measure the same underlying construct. The internal consistency reliability was assessed by calculating Guttman’s lambda (λ) coefficients. These coefficients encompass 6 measures (λ_1_–λ_6_) based on the analysis of the total variance of the index, the variance of each of its components, and the covariance between them. Briefly, to calculate each λ coefficient, the 3 parameters are set differently, such as summing the variance or computing the variance on a per-component basis, and different adjustments are applied, considering factors like the number of food components [45]. Special attention is focused on λ_4_, which represents the maximum split-half reliability and measures how all parts of an instrument contribute equally to what is being measured.

Structural equation modeling (SEM) was used to test the structural validity of the indices. Maximum likelihood estimation was adopted to determine the model fit when predicting the correlations among food components (observed variables) through a single underlying continuous latent variable (total index score). Recommended goodness-of-fit indices were calculated: χ^2^ to degree of freedom ratio (χ^2^/df), root mean square error of approximation, comparative fit index (CFI), standardized root mean square residual, and coefficient of determination (CD). For HSDI, ELDS, and DI, generalized structural equation modeling (GSEM) was used, as they are based on a binary scoring; thus, goodness-of-fit indices could not be calculated.

#### Index variability and relationship with total energy intake

The percentile distribution, ranging from the first to the 99th percentile, was computed for the 9 indices to assess their ability to capture dietary variability, which is crucial for nutritional metrics as it indicates sensitivity to detect sufficient data variation [24]. Likewise, Pearson’s coefficients were calculated between the indices and total energy intake (TEI) to test if these are independent of the TEI.

#### Examination of concordance between indices

Furthermore, the degree of concordance among indices was analyzed [46]. For this, we determined the proportion of participants classified in the same quintile, the adjacent quintile, and the opposite extreme quintile in relation to the indices based on the total score. Quartiles were employed instead of quintiles for binary indices due to the limited range of variation in total scores, which can result in imbalanced categories. Alluvial diagrams were used to visualize transitions between quantiles (that is, quintiles or quartiles). Additionally, Fleiss’ kappa (κ) coefficients were calculated, with a score of 1 indicating perfect concordance, whereas a score close to 0 suggests poor concordance between the indices. The κ coefficients were interpreted as follows: <0.00 as poor; 0.00–0.20 as slight; 0.21–0.40 as fair; 0.41–0.60 as moderate; 0.61–0.80 as substantial, and 0.81–1.0 as almost perfect concordance [47].

#### Analyses of indices according to sociodemographic variables

Comparing different demographic groups with known differences allows for the evaluation of concurrent-criterion validity (that is, measurement performance when assessed against an external criterion) [24]. Therefore, the means of the total scores were compared across different sociodemographic groups using analysis of covariance (ANCOVA). Additionally, the Jonckheere–Terpstra trend test was conducted to examine the trend of the indices across demographic groups.

#### Analysis of convergent validity using validated nutritional quality and environmental indicators

Measures related to a particular phenomenon are expected to be highly correlated, suggesting that they converge and are measuring the same underlying construct [25]. Therefore, because the 9 indices based on the EAT-Lancet recommendations have been proposed as a measure of healthy and sustainable diets, correlations with nutritional and environmental indicators were analyzed by calculating Spearman’s coefficients (ρ).

#### Analysis of trends across quintiles

A series of analyses of variance (ANOVA) was used to test the means of PANDiet and environmental impact indicators among quintiles/quartiles of the 9 indices. ANOVA effect sizes were expressed as partial eta-squared coefficients (η^2^) to describe the proportion of the total variation of score that can be attributed to each variable. The η^2^ cut-off points were as follows: 0.01 (small effect), 0.06 (moderate effect), and 0.14 (large effect). Trends were assessed using the Jonckheere–Terpstra test. Violin plots were used to visually illustrate the differences in PEF and PANDiet between the lowest and the highest quintiles/quartiles.

## Results

### Descriptive characteristics

**FIGURE 1:**
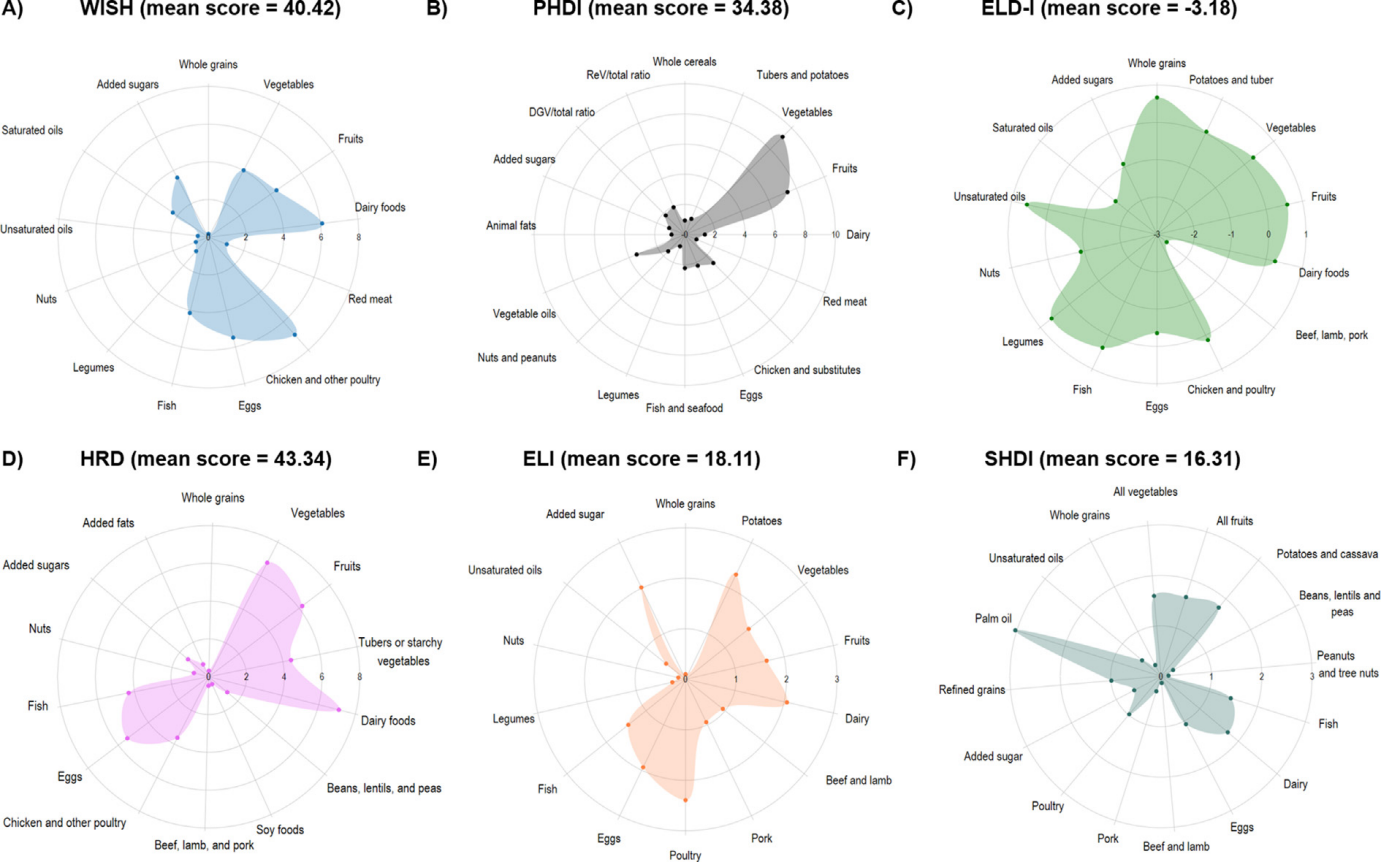
Mean scores of the food components of EAT-Lancet-based indices in the French Third Individual and National Study on Food Consumption Survey (INCA3, *n* = 1723).

The 9 indices presented a normal distribution (Supplemental Figure 2). As for the WISH, the score ranged from 2 to 97 points, with a mean of 40.42 points (SE = 0.37). Differences were observed in the scores for the food components (Figure 1). In particular, the chicken and other poultry, dairy foods, and eggs food components obtained mean scores above 5 points. In contrast, whole grains, unsaturated oils, nuts, and legumes were the groups with the lowest scores, all with means below one point.

Regarding the PHDI index, the mean total score was 34.38 points (SE = 0.28), within a range that varied between 2.89 and 78.60. The food components that obtained the highest scores were vegetables, fruits, vegetable oils, and chicken and substitutes, whereas the red meat, legumes, animal fats, whole cereals, tubers and potatoes, and added sugars components showed scores below 1 point (Figure 1).

Regarding the ELD-I index, the mean for the total score was –3.18 points (SE = 0.81), ranging between –113.11 and 104.55 points. The food components with the highest scores were unsaturated oils, whole grains, legumes, fish, and fruits, whereas the lowest scores were for beef, lamb and pork, saturated oils, added sugars, nuts, and eggs, all with negative scores (Figure 1).

The mean HRD score was 43.34 (SE = 0.31), with values ranging from 6.98 to 93.28. Vegetables, fruits, and dairy foods received the highest scores, whereas whole grains, nuts, beef, lamb, and pork, and soy foods had the lowest scores (Figure 1).

In relation to the ELI index, the total score ranged from 7 to 32 points, with a mean of 18.11 (SE = 0.10). The food components with the highest scores were poultry, potatoes, dairy, and added sugars, whereas whole grains, nuts, legumes, unsaturated oils, beef, and lamb and pork registered lower scores (Figure 1).

As for the SHDI, the mean score was 16.31 (SE = 0.10), with values ranging from 5 to 32 points. The food components receiving the highest scores included palm oil, all vegetables, all fruits, potatoes and cassava, and dairy. In contrast, whole grains, peanuts and tree nuts, beans, lentils, peas, pork, beef, and lamb had the lowest mean scores (Figure 1).

**FIGURE 2:**
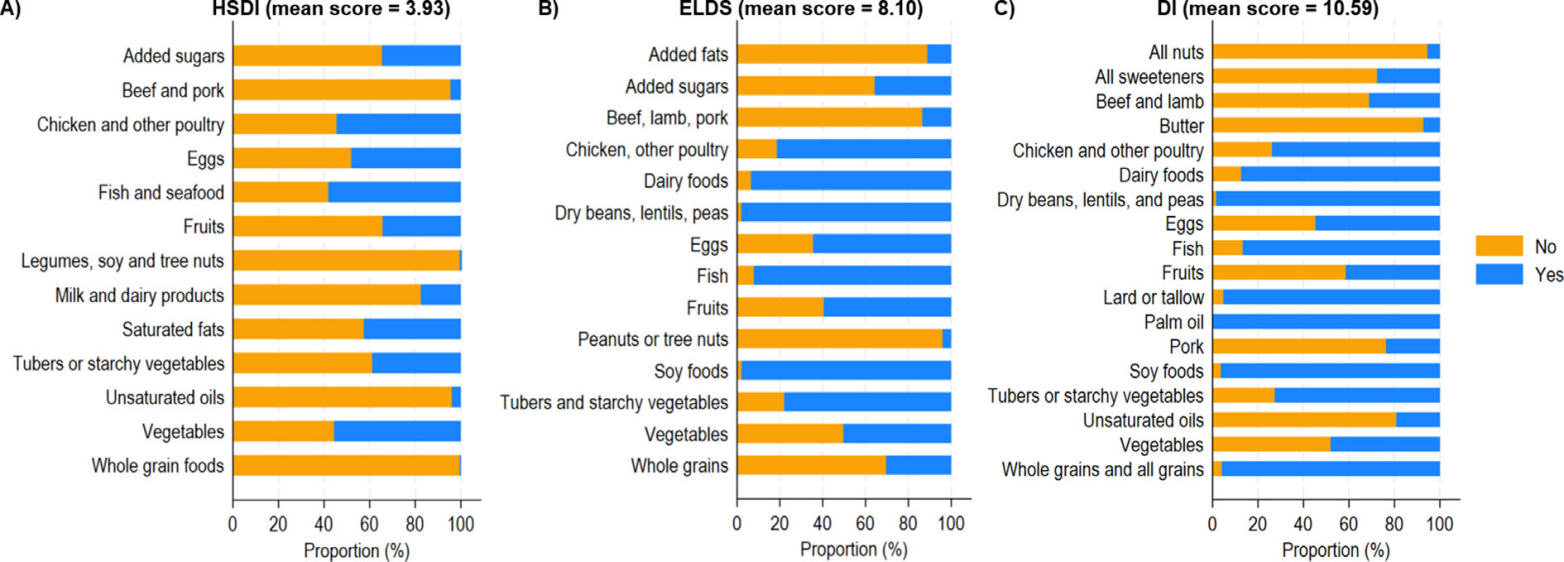
Proportion of participants adhering to EAT-Lancet recommendations through binary indices in the French Third Individual and National Study on Food Consumption Survey (INCA3, *n* = 1723).

On the other hand, the indices based on a binary scoring showed a dissimilar behavior. In this sense, the HSDI had a mean of 3.93 points (SE = 0.04) in a range that varied between 0 and 10 points. The components with the highest proportion of participants meeting the recommendations were fish and seafood, vegetables, and chicken and other poultry, with over 50% compliance (Figure 2). In contrast, <1% of participants met the target intake for legumes, soybeans and tree nuts, and whole grain foods. For the ELDS, the mean was 8.10 points (SE = 0.04, range = 4–12), with dry beans, lentils and peas, soy foods, dairy foods, and fish being the groups with the highest target compliance (Figure 2). Conversely, peanuts and tree nuts, added fats and beef, lamb and pork were less compliant. Finally, the mean DI score was 10.59 (SE = 0.04, range 7–16), with palm oil, dry beans, lentils, peas, soy foods, dairy foods, lard or tallow, and whole and all grains receiving the highest compliance scores. In contrast, the food components with the lowest proportion of participants meeting the recommendations included all nuts, butter, all sweeteners, unsaturated oils, and beef and lamb (Figure 2).

### Reliability and structural validity

ELD-I was the index with the highest λ coefficients, mainly for split-half reliability (λ4 = 0.57), followed by WISH (λ4 = 0.47), ELI (λ4 = 0.47), and PHDI (λ4 = 0.46). Conversely, the DI had the lowest λ coefficients (<0.40). Moreover, all intercomponent correlations were below 0.80, suggesting an absence of redundancy. Furthermore, all items contributed significantly to the total score as the component-total correlations were statistically significant, with the exception of chicken and poultry from the ELD-I, legumes from the HSDI, and soy foods from the ELDS. Curves with λ coefficients and correlation matrices are presented in Supplemental Material (Supplemental Figure 3 and Supplemental Tables 16–24).

SEM models confirmed the unidimensional structural validity of the WISH, PHDI, ELD-I, HRD, ELI, and SHDI indices (Table 4). Although the indices showed a similar fit profile, the SHDI, PHDI, and ELD-I were the most robust in explaining the data variability (CD = 0.617, 0.568, and 0.466, respectively) and had high incremental indices (CFI > 0.90). Because HSDI, ELDS, and DI are composed of dichotomous items, they were modeled by GSEM, which confirmed their unidimensional structure (results not shown).

**TABLE 4:** Fit indices for confirmatory factor analysis.

	Expected values	WISH	PHDI	ELD-I	HRD	ELI	SHDI
χ2/df	2.50	1.65	1.77	2.34	1.70	1.60	1.62
RMSEA	<0.08	0.019	0.021	0.028	0.020	0.019	0.019
CFI	≥0.90	0.922	0.904	0.911	0.903	0.928	0.900
SRMR	<0.05	0.025	0.027	0.028	0.026	0.023	0.024
CD	The higher, the better	0.323	0.568	0.466	0.441	0.364	0.617

Abbreviations: χ^2^/df: Chi-square to degree of freedom ratio; CD, coefficient of determination; CFI, Comparative Fit Index; RMSEA, Root Mean Square Error of Approximation; SRMR, Standardized Root Mean Square Residual.

### Capture of diet variability and energy independence

ELD-I presented the greatest difference in scores between the first percentile (–84.01) compared with the 99th percentile (74.72), followed by WISH (6.92 compared with 75.44), HRD (16.00 compared with 76.42), and PHDI (10.31 compared with 63.62). HSDI and ELDS showed the lowest change across percentiles (Supplemental Figure 4). In addition, the correlation of the indices with TEI was analyzed. TEI showed no correlation with SHDI and negligible correlation with DI (r = –0.081), ELD-I (r = –0.106), PHDI (r = –0.088), and HRD (r = –0.106). Regarding the other indices, correlations with energy intake were low (*P* < 0.0001): for HSDI, r = –0.227; for WISH, r = –0.254; for ELI, r = –0.279; and for ELDS, r = –0.306. More details are available in Supplemental Table 25.

### Interindex concordance

**FIGURE 3:**
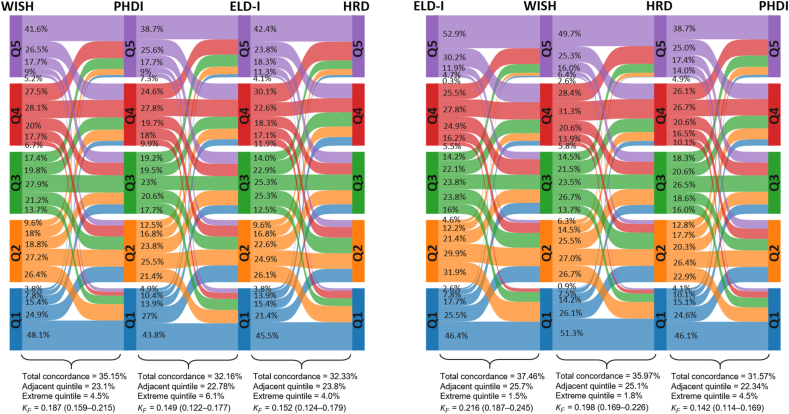
Interindex concordance among EAT-Lancet-based indices quintiles in the French Third Individual and National Study on Food Consumption Survey (INCA3, *n* = 1723). Proportion of participants classified in the same quintile (total concordance), the adjacent quintile, and the opposite extreme quintile. KF, Fleiss’ kappa.

**FIGURE 4:**
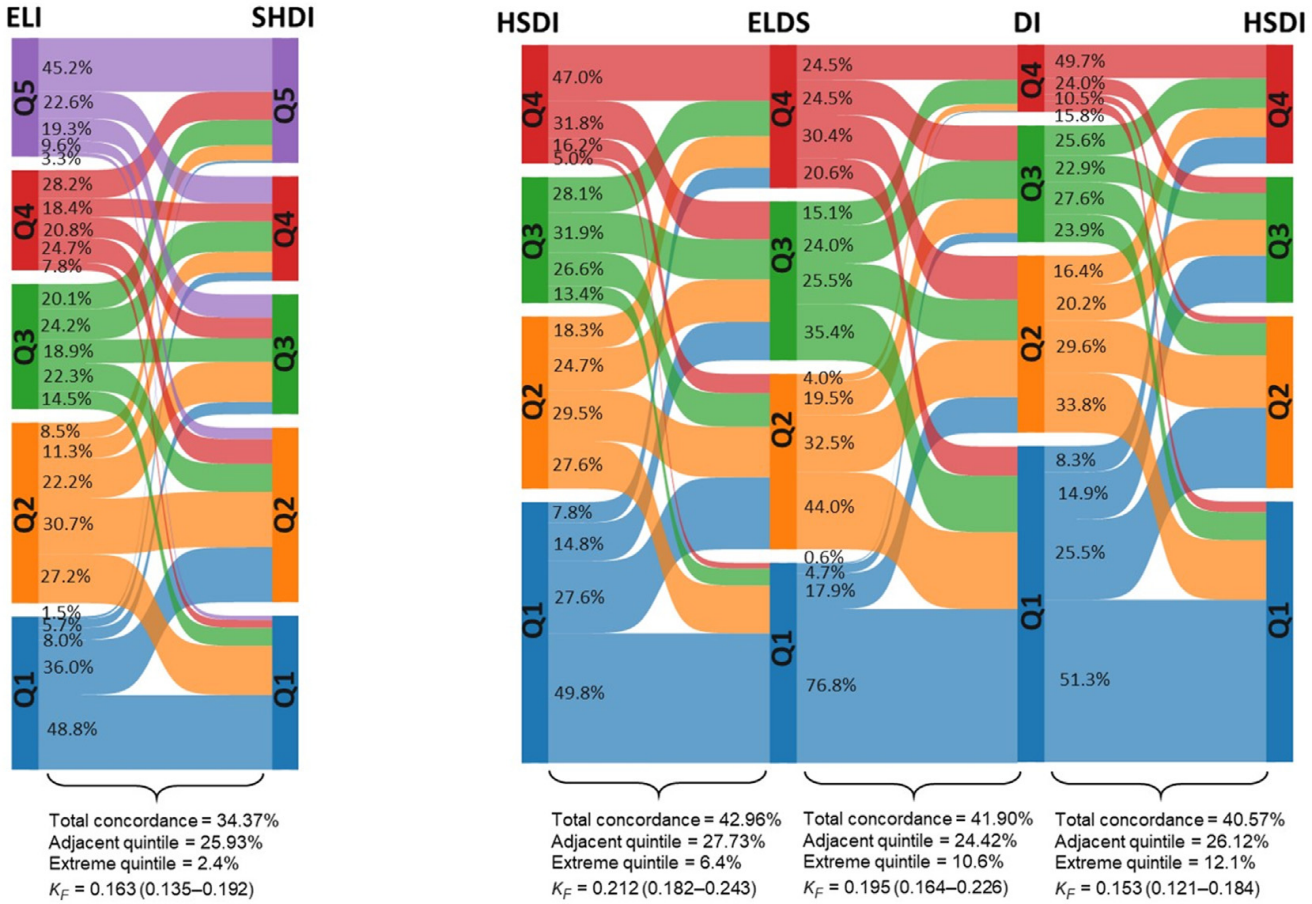
Interindex concordance among EAT-Lancet-based indices quintiles/quartiles in the French Third Individual and National Study on Food Consumption Survey (INCA3, *n* = 1723). Quintiles were calculated for graded scoring indices, whereas quartiles were used for binary indices. Proportion of participants classified in the same quintile/quartile (total concordance), the adjacent quintile/quartile, and the opposite extreme quintile/quartile. KF, Fleiss’ kappa.

The alluvial plots in FIGURE 3, FIGURE 4 show the concordance among the indices. Total concordance (that is, individuals classified in the same quintile/quartile) was below 50% for all paired comparisons. Moreover, between 22% and 28% of the participants were classified in adjacent quintiles/quartiles. The classification percentages in the opposite extreme quintile/quartile ranged from 1% to 12%. Moreover, κ coefficients indicated slight or fair concordance between the indices (FIGURE 3, FIGURE 4).

#### Concurrent-criterion validity

According to ANCOVA models (Table 5), women had significantly higher means in WISH, ELI, PHDI, and ELDS scores than men. In addition, older age groups scored higher on all indices, except for DI. On the other hand, the means in PHDI, ELD-I, and ELI were significantly different according to educational level, with those with higher level having a higher mean score than those with lower formal education. Likewise, those individuals with higher income had higher scores in all indices, with the exception of SHDI and HSDI. Regarding weight status, individuals in the lower BMI groups had higher scores; however, the trend was only confirmed for ELD-I and ELI. Conversely, nonsmokers have higher means of WISH, ELD-I, and ELI. The level of physical activity was only related to the WISH, ELD-I, HRD, and SHDI indices.

**TABLE 5:** Association between each EAT-Lancet index and sociodemographic characteristics in adults from the French Third Individual and National Study on Food Consumption Survey (INCA3).

Characteristics (%)	WISH	PHDI	ELD-I	HRD	ELI	SHDI	HSDI	ELDS	DI
Sex									
Women (58%)	41.49 (0.49)	34.86 (0.35)	–1.37 (1.12)	44.10 (0.42)	18.72 (0.12)	16.63 (0.14)	3.94 (0.05)	8.34 (0.05)	10.60 (0.04)
Men (42%)	39.34 (0.57)	33.90 (0.44)	–5.01 (1.18)	42.58 (0.46)	17.48 (0.15)	15.99 (0.16)	3.92 (0.06)	7.85 (0.05)	10.57 (0.06)
*F*	7.88	4.45	0.69	1.26	25.09	2.05	0.67	45.04	0.19
*P value*	0.0051	0.0351	0.4059	0.2622	<0.0001	0.1521	0.4129	<0.0001	0.6610
*P-for-trend*	n/a	n/a	n/a	n/a	n/a	n/a	n/a	n/a	n/a
Age									
18–44 y old (36%)	35.61 (0.59)	31.67 (0.45)	–9.75 (1.25)	40.18 (0.49)	16.95 (0.16)	14.87 (0.16)	3.61 (0.06)	7.78 (0.06)	10.45 (0.06)
45–64 y old (39%)	42.49 (0.57)	36.21 (0.43)	–1.59 (1.36)	44.89 (0.49)	18.82 (0.15)	17.04 (0.16)	4.12 (0.06)	8.22 (0.06)	10.71 (0.06)
≥65 y old (25%)	48.93 (0.68)	37.79 (0.55)	10.99 (1.52)	48.50 (0.60)	19.72 (0.18)	18.65 (0.19)	4.39 (0.07)	8.69 (0.07)	10.70 (0.07)
*F*	49.36	30.65	26.70	33.69	49.26	69.29	13.8	25.44	2.14
*P* value	<0.0001	<0.0001	<0.0001	<0.0001	<0.0001	<0.0001	<0.0001	<0.0001	0.1186
*P for-trend*	<0.0001	<0.0001	<0.0001	<0.0001	<0.0001	<0.0001	<0.0001	<0.0001	0.0313
Education									
Primary and middle school (37%)	41.89 (0.59)	34.40 (0.46)	–3.75 (1.37)	43.33 (0.50)	18.17 (0.15)	16.50 (0.17)	4.08 (0.07)	8.12 (0.06)	10.63 (0.06)
High school (20%)	37.13 (0.89)	32.04 (0.59)	–11.04 (1.86)	41.25 (0.70)	17.10 (0.21)	15.68 (0.23)	3.69 (0.09)	7.83 (0.08)	10.40 (0.08)
1 to 3 y of post-secondary education (22%)	40.64 (0.86)	35.84 (0.63)	1.92 (1.66)	44.99 (0.69)	18.66 (0.23)	16.40 (0.23)	3.94 (0.08)	8.11 (0.08)	10.51 (0.08)
≥4 y of post-secondary education (21%)	39.65 (0.76)	35.17 (0.58)	0.93 (1.58)	43.78 (0.65)	18.37 (0.21)	16.38 (0.22)	3.78 (0.08)	8.29 (0.07)	10.73 (0.07)
*F*	1.37	7.01	4.24	1.95	5.71	1.61	0.82	2.34	1.92
*P value*	0.2501	0.0001	0.0054	0.1203	0.0007	0.1862	0.4815	0.0714	0.1251
*P-for-trend*	0.5636	0.0003	0.0042	0.0106	0.0076	0.4021	0.5076	0.0008	0.0293
Monthly income^1^									
<900 €/month/CU (17%)	37.47 (0.87)	33.03 (0.75)	–13.23 (1.96)	40.76 (0.71)	17.28 (0.24)	15.68 (0.23)	3.94 (0.10)	7.71 (0.09)	10.39 (0.09)
900–1340 €/month/CU (22%)	40.07 (0.85)	32.79 (0.62)	–2.85 (2.00)	42.44 (0.69)	17.99 (0.22)	16.33 (0.23)	3.81 (0.09)	8.12 (0.07)	10.48 (0.08)
1340–1850 €/month/CU (24%)	41.89 (0.81)	35.24 (0.63)	0.99 (1.69)	44.08 (0.66)	18.65 (0.21)	16.43 (0.22)	3.99 (0.08)	8.20 (0.08)	10.67 (0.08)
≥1850 €/month/CU (37%)	42.02 (0.64)	35.83 (0.42)	0.88 (1.27)	45.57 (0.53)	18.56 (0.17)	16.93 (0.18)	4.01 (0.07)	8.33 (0.06)	10.73 (0.06)
*F*	3.2	3.52	6.83	4.17	2.94	1.03	1.30	6.86	3.07
*P value*	0.0225	0.0146	0.0001	0.0060	0.0322	0.3773	0.2729	0.0001	0.0268
*P for-trend*	<0.0001	<0.0001	0.0002	<0.0001	<0.0001	<0.0001	0.0748	<0.0001	0.0058
Weight status									
Underweight (3%)	33.96 (2.50)	29.75 (1.92)	–17.92 (5.90)	39.48 (2.28)	16.22 (0.77)	15.49 (0.67)	3.29 (0.22)	7.41 (0.24)	10.56 (0.23)
Normal (49%)	39.42 (0.54)	33.59 (0.40)	–0.83 (1.09)	43.47 (0.43)	18.30 (0.14)	16.12 (0.14)	3.86 (0.06)	8.12 (0.05)	10.60 (0.05)
Overweight (34%)	42.68 (0.63)	35.71 (0.46)	–3.24 (1.39)	44.30 (0.53)	18.26 (0.15)	16.59 (0.18)	4.10 (0.07)	8.19 (0.06)	10.66 (0.06)
Obesity (10%)	40.17 (1.06)	35.02 (0.87)	–9.43 (2.91)	42.51 (0.89)	17.58 (0.29)	16.73 (0.30)	3.90 (0.13)	8.01 (0.10)	10.40 (0.10)
Morbid obesity (4%)	38.51 (1.98)	35.11 (1.33)	–1.31 (4.26)	37.52 (1.78)	17.37 (0.46)	15.59 (0.57)	4.02 (0.19)	7.85 (0.20)	10.27 (0.18)
*F*	4.77	2.78	5.95	7.10	7.92	1.77	3.30	4.20	1.92
*P value*	0.0008	0.0256	0.0001	<0.0001	<0.0001	0.1331	0.0105	0.0022	0.1042
*P for-trend*	0.7019	0.7524	0.0853	0.5536	0.0180	0.0778	0.1508	0.1261	0.0678
Smoking status^2^									
No (75%)	41.66 (0.43)	35.15 (0.32)	–0.25 (0.92)	44.44 (0.36)	18.43 (0.11)	16.75 (0.12)	3.96 (0.04)	8.20 (0.04)	10.60 (0.04)
Yes (25%)	36.68 (0.72)	32.64 (0.54)	–12.24 (1.67)	40.13 (0.61)	17.12 (0.20)	15.07 (0.20)	3.87 (0.09)	7.80 (0.07)	10.55 (0.07)
*F*	7.82	2.20	14.01	15.90	9.59	20.11	0.27	3.65	0.16
*P value*	0.0052	0.1387	0.0002	0.0001	0.0020	<0.0001	0.6047	0.0561	0.6880
*P-for-trend*	n/a	n/a	n/a	n/a	n/a	n/a	n/a	n/a	n/a
Physical activity									
Low (38%)	39.17 (0.62)	33.34 (0.43)	–5.14 (1.32)	42.71 (0.54)	17.90 (0.16)	16.19 (0.18)	3.79 (0.06)	8.01 (0.06)	10.47 (0.06)
Moderate (51%)	41.38 (0.55)	35.02 (0.43)	–2.57 (1.19)	43.92 (0.44)	18.31 (0.14)	16.50 (0.14)	4.02 (0.06)	8.14 (0.05)	10.66 (0.05)
High (11%)	39.01 (1.18)	33.43 (0.87)	–1.59 (2.50)	43.06 (0.90)	17.61 (0.32)	16.02 (0.33)	3.82 (0.12)	7.93 (0.11)	10.58 (0.11)
*F*	3.01	2.74	0.51	0.10	2.59	0.07	2.71	2.95	1.75
*P value*	0.0498	0.0328	0.5985	0.9070	0.0750	0.9307	0.0671	0.0524	0.1749
*P-for-trend*	0.0388	0.1345	0.0011	0.0857	0.0692	0.0420	0.1958	0.8481	0.0803

*P* value = *P* referred to ANCOVA; *P*-for-trend = P referred to Jonckheere–Terpstra test for trend ordered predictors. n/a, not applicable.

1 Income per consumption unit.

2 “Currently smoking” behavior.

#### Convergent validity: correlation with nutritional measures

The mean PANDiet score was 64.83 (SE = 0.13). Correlations were the lowest (ρ < 0.15) for the HSDI and ELDS binary indices, null for DI, and ranged from 0.22 to 0.34 among other indices. Moreover, significant positive correlations were found between the adequacy subscore (mean = 63.42; SE = 0.29) and the WISH, PHDI, ELD-I, HRD, ELI, and SHDI indices, with ρ ranging from 0.07 to 0.31. Regarding the moderation subscore (mean = 66.23; SE = 0.24), most indices were positively related (ρ between 0.06 and 0.21) with PHDI and ELD-I exhibiting the lowest correlation coefficients. Conversely, SHDI and DI showed no significant correlation with moderation.

When analyzing the adequacy at nutrient level, the results behaved differently according to the scoring system used. In general, the HSDI and ELDS indices (both based on a binary scoring) correlated inversely with several nutrients: protein, DHA, EPA+DHA, riboflavin, niacin, pantothenic acid, vitamin B-6, vitamin B-12, vitamin D, iodine, phosphorus, zinc, calcium, and iron. Similarly, the DI index was negatively associated with some of these nutrients, whereas most other correlations were not significant.

**FIGURE 5:**
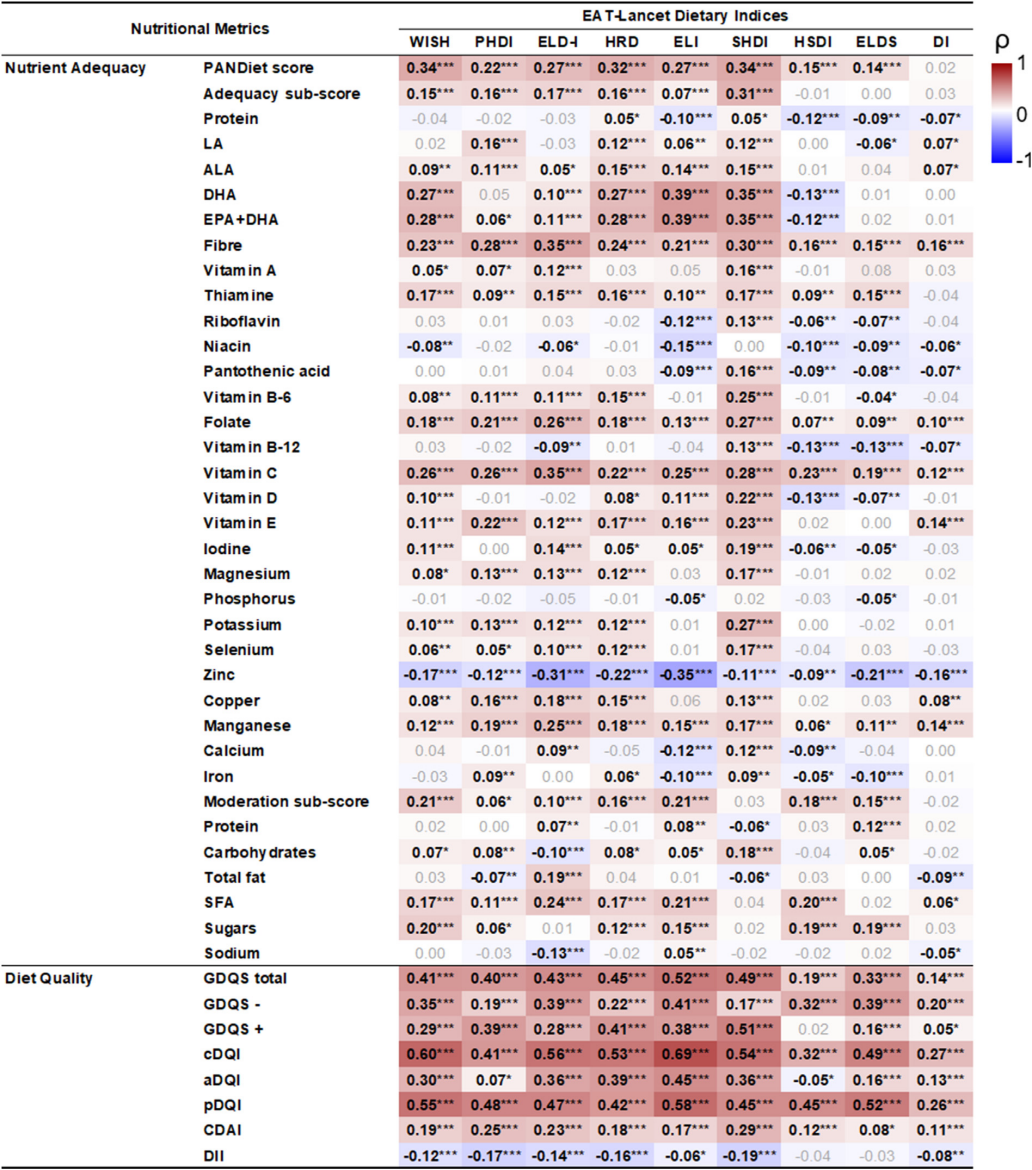
Correlations between the nutritional variables and the EAT-Lancet-based indices in the French Third Individual and National Study on Food Consumption Survey (INCA3, *n* = 1723). Heat map plotting Spearman’s correlation coefficients (ρ): red indicates positive correlations, white indicates no correlations, and blue indicates negative correlations. PANDiet, Probability of Nutritional Adequacy. ∗*P* < 0.05, ∗∗*P* < 0.001, ∗∗∗*P* < 0.0001.

On the other hand, indices using proportional scoring were positively correlated with the adequacy of most nutrients, including polyunsaturated fatty acids, vitamins (for example, A, thiamine, B-6, and E), and minerals (for example, manganese, magnesium, copper, and selenium). However, certain negative associations were found between some of these indices. In this sense, the ELD-I correlated inversely with vitamin B12, carbohydrates, and sodium, whereas the WISH and PHDI correlated inversely with niacin and total fat, respectively. Regarding the indices based on graded scoring, SHDI demonstrated the strongest correlations with most nutrients but showed a very weak inverse association with the moderation of protein and total fats. ELI shared traits with both proportional and binary ones. Although it showed positive associations with several indicators, such as polyunsaturated fatty acids and vitamins D and E, it was negatively related to others, such as protein, B-complex vitamins, phosphorus, calcium, and iron. The likelihoods of adequacy of fiber, thiamine, folate, vitamin C, and manganese were positively related to all 9 indices. Likewise, the likelihood of zinc adequacy correlated inversely with all of them. More details of the correlations are shown in Figure 5.

Furthermore, the indices showed significant correlations with other nutritional quality scores. The 9 indices were positively associated with the GDQS (correlation coefficients between 0.14 and 0.52) and cDQI total scores (correlation coefficients between 0.27 and 0.69). In addition, most indices were associated with an antioxidant and anti-inflammatory diet, except the HSDI and ELDS. As such, these indices showed positive and weak correlations with CDAI (correlation coefficients between 0.08 and 0.29) and negative very weak correlations with DII (correlation coefficients between –0.06 and –0.19). Conversely, negligible or null correlations were found between the indices based on binary scoring and DII and CDAI.

#### Convergent validity: correlation with environmental impact indicators

**FIGURE 6:**
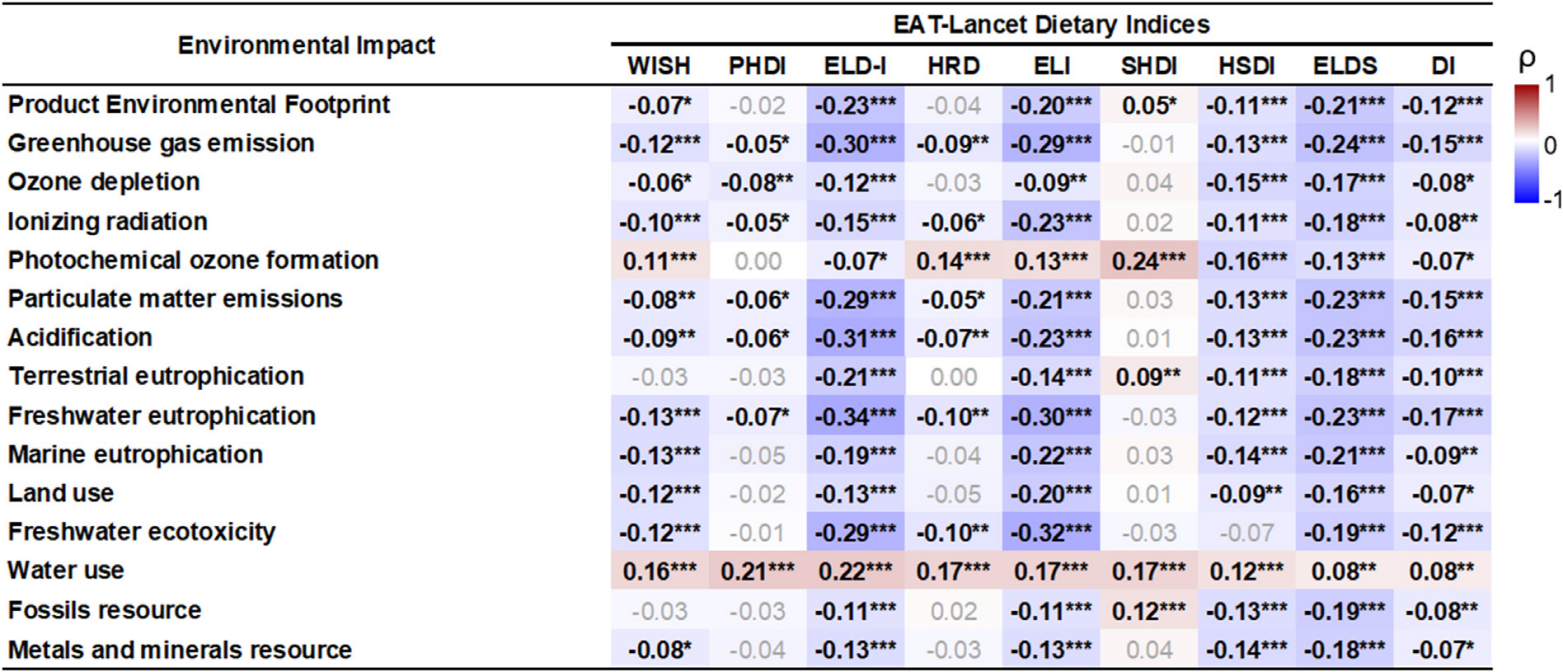
Correlations between the Environmental Impact and the EAT-Lancet-based indices in the French Third Individual and National Study on Food Consumption Survey (INCA3, *n* = 1723). Heat map plotting Spearman correlation coefficients (ρ): red indicates positive correlations, white indicates no correlations, and blue indicates negative correlations. The product environmental footprint is an aggregated indicator of the 14 environmental metrics. ∗*P* < 0.05, ∗∗*P* < 0.001, ∗∗∗*P* < 0.0001.

Figure 6 shows a heat map for the correlation analysis between the indices and the aggregated indicator PEF and 14 individual metrics. Overall, the indices were negatively correlated with the indicators (highest ρ = –0.34), with the exception of water use and photochemical ozone formation, this latter only for WISH, HRD, ELI, and SHDI. However, it is important to mention that the highest correlations were found for ELD-I and ELI, whereas SHDI and PHDI was the index that showed the weakest (ρ< –0.10) and least significant coefficients. Moreover, positive correlations were observed between SHDI and PEF, terrestrial eutrophication, and fossil resource use.

### Trends across level of adherence

**FIGURE 7:**
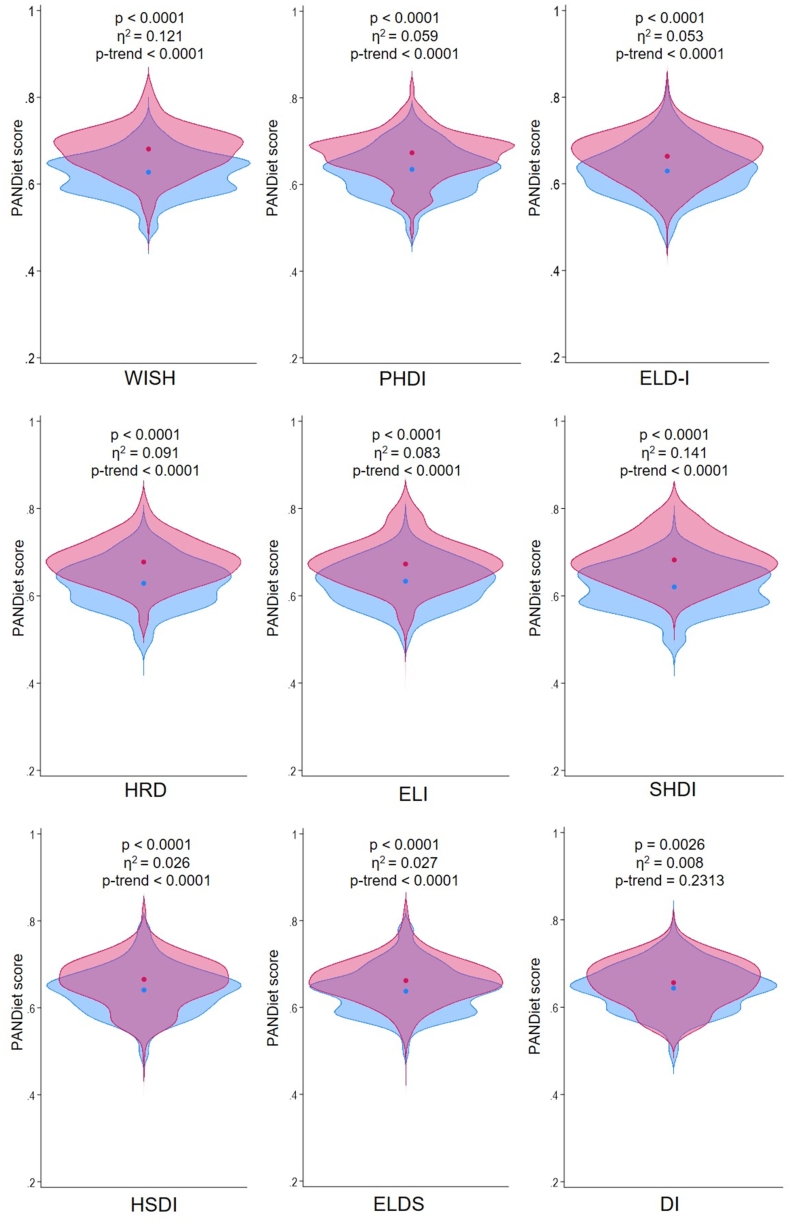
Violin plots comparing the distribution of PANDiet scores between the highest (in red) and lowest quintile/quartile (in blue) of EAT-Lancet-based indices in the French Third Individual and National Study on Food Consumption Survey (INCA3, *n* = 1723). *P* values and effect sizes obtained by ANOVA comparisons. The Jonckheere–Terpstra test for trend was used. The circles denote the mean values.

The probability of nutritional adequacy was compared across quintiles/quartiles to identify trends. Regarding the PANDiet score, all indices exhibited significant differences between quantiles, with the indices using a proportional scoring system showing the best performances (Figure 7). However, the SHDI, WISH, HRD, and ELI indices revealed moderate-to-large effects on the PANDiet index (η^2^ = 0.141, 0.121, 0.091, and 0.083, respectively), whereas the effects of PHDI and ELD-I were small in magnitude (η^2^ = 0.059 and 0.053, respectively). Differences were also found when analyzing particular trends at the level of the PANDiet score components (Supplemental Tables 26–34). In this regard, the WISH had its main effects on vitamin C, the moderation subscore, and sugars, whereas the PHDI index had the least significant changes, with its main effect on fiber and vitamin C. As for ELD-I index, positive trends with moderate-to-large effect sizes were found for vitamin C, fiber, folate, and manganese. Additionally, significant differences were found, albeit of small magnitude, for polyunsaturated fatty acids (ALA, DHA, and EPA+DHA), vitamins (A, riboflavin, niacin, pantothenic acid, B-6, D, and E), and minerals (iodine, magnesium, potassium, selenium, copper, and calcium). Although protein, LA, vitamin B-12, zinc, iron, carbohydrates, and sodium components showed a negative trend across the ELD-I quintiles, all were of small magnitude, except for zinc, which had a moderate effect size, or even showed no significant trends, as was the case with iron and proteins. On the other hand, noteworthy are the moderate-sized positive trends of the ELI and HRD indices for DHA, EPA+DHA, vitamin C, and negative trends with respect to zinc. Significant differences were observed for the majority of micronutrients across SHDI quantiles, showing positive trends with moderate effect sizes in the adequacy of DHA, EPA+DHA, fiber, vitamins B6 and C, folate, iodine, and potassium. Regarding the HSDI, ELDS, and DI indices, significant differences and trends were infrequent and of small magnitude.

**FIGURE 8:**
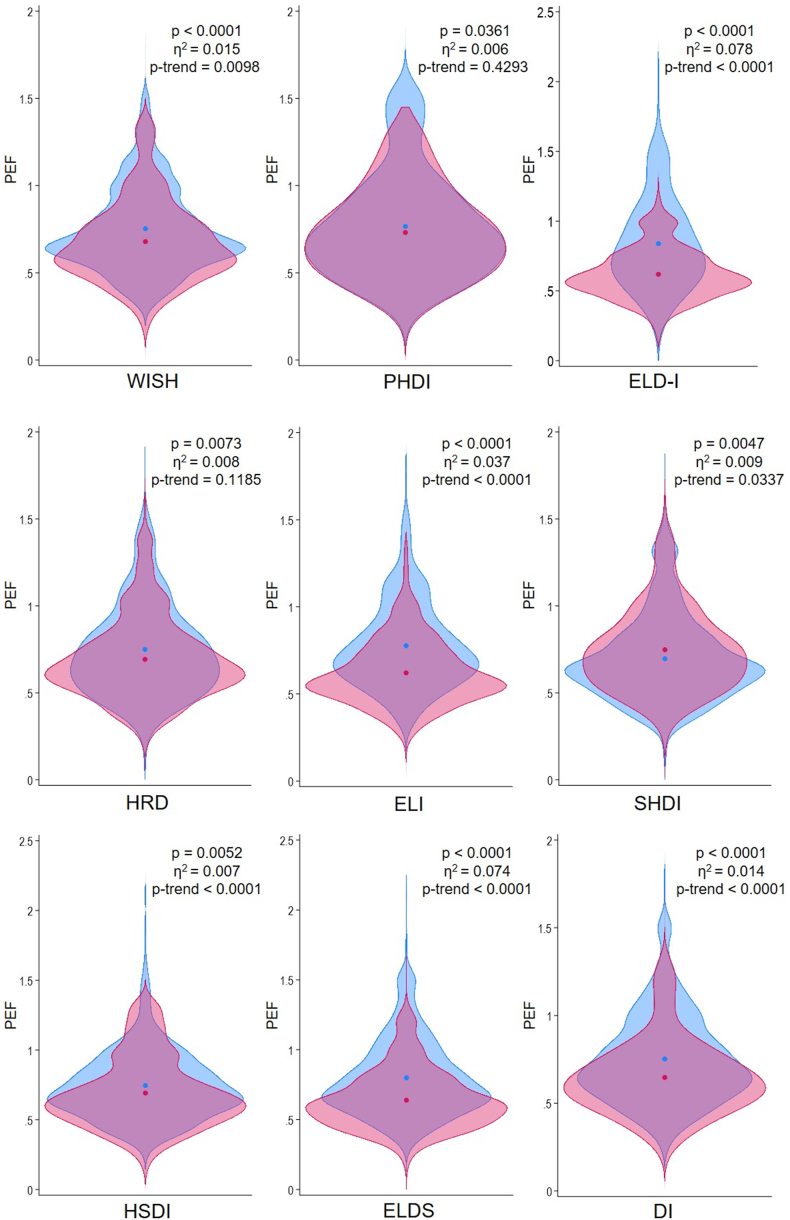
Violin plots comparing the distribution of product environmental footprint (PEF) between the highest (in red) and lowest quintile/quartile (in blue) of EAT-Lancet-based indices in the French Third Individual and National Study on Food Consumption Survey (INCA3, *n* = 1723). *P* values and effect sizes obtained by ANOVA comparisons. The Jonckheere–Terpstra test for trend was used. The circles denote the mean values.

As displayed in Figure 8, the greatest difference in PEF was found between the quintiles of ELD-I (*P* < 0.0001), with a moderate effect size (η^2^ = 0.081) and an inverse trend (*P* < 0.0001). Significant differences across quantiles were observed for most indices, with the exception of the PHDI and HRD indices, which did not exhibit significant trends. Importantly, SHDI was the only index that exhibited a positive trend in PEF, although the effect size was small. Regarding the specific environmental metrics (Supplemental Tables 35–43), the ELD-I index also showed the strongest differences, displaying negative trends with a strong effect on freshwater eutrophication and moderate effects on GHGE, particulate matter emissions, acidification, and freshwater ecotoxicity. Furthermore, it demonstrated a negative relationship with the other environmental indicators, excepting water use that was positive. Among the other indices, ELI stood out, exhibiting negative trends in GHGE, freshwater eutrophication, and freshwater ecotoxicity, with small effects on the other indicators. Additionally, although significant differences were found across quintiles of WISH (all of small magnitude), trends were ruled out for terrestrial eutrophication and fossil resource. HRD also exhibited differences of small magnitude for most indicators, with the exceptions of marine eutrophication, fossil resource use, and metals and minerals resources. No significant trends were observed for PEF, ozone depletion, and terrestrial eutrophication. SHDI exhibited the smallest number of environmental impact indicators that differed across quintiles, with even positive trends observed for photochemical formation, terrestrial eutrophication, water use, and fossil resource. On the side of binary scoring indices, ELDS was associated with negative effects on a greater number of indicators than HSDI and DI. Similar to ELD-I, ELDS showed negative trends with moderate effects for GHGE, particulate matter emissions, acidification, and freshwater eutrophication.

On the other hand, PANDiet/PEF ratio correlated significantly with WISH (ρ = 0.17, *P* < 0.0001), PHDI (ρ = 0.08, *P* = 0.0006), ELD-I (ρ = 0.31, *P* < 0.0001), HRD (ρ = 0.13, *P* < 0.0001), ELI (ρ = 0.28, *P* < 0.0001), HSDI (ρ = 0.16, *P* < 0.0001), ELDS (ρ = 0.26, *P* < 0.0001), and DI (ρ = 0.13, *P* < 0.0001). SHDI did not correlate with the PANDiet/PEF ratio (ρ = 0.04, *P* = 0.0766). Furthermore, positive and significant trend was observed in the relationship between the PANDiet/PEF ratio across quintiles/quartiles, with moderate size effects for ELD-I (η^2^ = 0.088, p-for-trend <0.0001), ELDS (η^2^ = 0.089, p-for-trend < 0.0001) and ELI (η^2^ = 0.076, p-for-trend < 0.0001), and small effects for WISH (η^2^ = 0.040, p-for-trend < 0.0001), HRD (η^2^ = 0.022, p-for-trend < 0.0001), HSDI (η^2^ = 0.026, p-for-trend < 0.0001), and DI (η^2^ = 0.025, p-for-trend < 0.0001). However, no significant differences were found in this variable for the PHDI and SHDI indices. More details are available in Supplemental Tables 35–43.

Finally, the most pronounced differences in food intake across quantiles were observed for fruits, vegetables, and legumes, with higher consumption in the last quantile across all indices. In contrast, the intake of tubers, added sugars, and beef and lamb was lower in this quantile. The consumption of fish, nuts, and unsaturated oils appeared to be relatively consistent across quantiles, with a slight increase in the last quantile (Supplemental Figure 5).

## Discussion

This study is the first to comprehensively evaluate the validity and reliability of 9 dietary indices representing the EAT-Lancet reference diet, using a national representative sample. Although some of these indices have been partially validated in prior research, this study offers a comprehensive analysis of all indices on the same sample. Briefly, our findings indicate that the most reliable indices are those using proportional scoring, especially those adjusted for energy intake (for example, ELD-I), which robustly captured dietary variability, independently of energy intake. Although the indices proved to be unidimensional and concurrently valid in differentiating scores based on sociodemographic factors, there was discordance in the classifications of individuals. In addition, the 9 indices demonstrated varied associations with nutrition and environmental impact, with significant weak correlations to nutritional adequacy and environmental impact but stronger correlations to diet quality. Notably, findings highlight that indices based on proportional scoring were mainly associated with nutrition, whereas indices with binary scoring were more linked to environmental impact. Nonetheless, ELD-I was associated with both nutritional and environmental domains; however, WISH, HRD, and SHDI outperformed ELD-I concerning essential fatty acids and vitamins. DI demonstrated a poor association with nutritional health but a good correlation with environmental impact, whereas SHDI exhibited best correlation with nutritional health but the poorest with environmental impact. Furthermore, although all indices were associated with lower adequacy of certain nutrients, such as zinc, and higher water use, the magnitudes of these associations were relatively modest.

Reliability was evaluated by focusing on internal consistency and relationships between food components scores [24]. ELD-I and WISH exhibited the best internal consistency, whereas DI showed lower λ coefficients. Although there is no fixed rule for determining when λ is high enough, the context and researcher’s judgment are crucial, especially in nutrition, where lower coefficients are common due to the complexity of human diet [24,25,48]. Internal consistency is not strictly necessary, but knowing this property has implications for confidence in the indices [24].

Similar to previous reports, the impact of individual components on the total score varied significantly [15,21]. In this sense, fruits and vegetables demonstrated robust correlations, underscoring their importance in evaluating both health and sustainability. Conversely, whole grains and legumes exhibited weaker correlations, which could be due to the challenges associated with meeting targets for less frequently consumed foods. Although all food components contribute, the indices currently assign them equal weights despite variations in their impact on health and the environment [49,50], suggesting a potential improvement by assigning different weights to better reflect their relative impact [51,52]. Also, indices using proportional scoring captured more the interindividual variability, especially ELD-I, increasing the validity in assessing and comparing diets [53]. This was expected, as the ability to capture data variability depends on the type of measurement used. Unbounded continuous measures, such as ELD-I, allow detailed representation by covering a full range of values within an infinite spectrum. On the other hand, bounded continuous measures, such as WISH and PHDI, restrict variability to specific values within a finite set. In addition, measures based on binary indices (that is, HSDI, ELDS, and DI), by classifying food compounds into only 2 categories, further limit the representation of variability. Consequently, the selection of metric type can markedly shape the comprehensiveness and precision of the analysis. Furthermore, ELD-I, PHDI, and DI were not affected by the amount of energy consumed, unlike other indices that showed moderate inverse correlations with energy intake. This energy adjustment in ELD-I, PHDI, and DI [14,16,20] avoids biases associated with unbalanced calorie diets, where high energy intake may result in high scores [54].

Despite the theoretical expectation of significant correlations between indices assessing adherence to EAT-Lancet recommendations, our study found low concordance among indices. The limited concordance may be attributed to differences in index design, including components, thresholds, weighting, and scoring systems. These findings align with previous studies comparing indices for Mediterranean diet adherence [55,56], emphasizing the importance of considering methodological differences in interpreting similar results.

In terms of structural validity, the unidimensionality of all indices was confirmed, with optimal fit, indicating plausible representations of the underlying relationships between food components [57]. Moreover, SHDI, ELD-I, and PHDI explained the variability of the food components at 61%, 57%, and 47% supporting their robust structural validity. Unidimensionality, regardless of whether the concept encompasses multiple domains or facets, is a key requirement for instruments that rely on a “total score,” such as the EAT-Lancet indices. The unidimensionality found in this study suggests that the food components within each index are associated with a single concept of a healthy and sustainable diet, thus supporting the use of a total score to simplify its comprehension and applicability [58,59]. However, it is recommended that the use of total scores be complemented by a detailed analysis of the food components. Furthermore, differences according to sociodemographics were found, supporting their concurrent-criterion validity. Although some indices did not reach statistical significance, the general pattern indicated that scores were higher in women, older individuals, with higher income, higher education, lower BMI, no-smokers, and physically active. These differences among demographic groups are consistent with previous studies on EAT-Lancet recommendations [[60], [61], [62], [63], [64]].

Regarding convergent validity, the indices presented variations in their correlation with nutritional adequacy. Among the indices evaluated, the SHDI exhibited the strongest positive correlations with the majority of the nutrients assessed. This result was expected, as the scoring system of the SHDI was adapted to avoid assigning positive scores for the nonconsumption of certain foods, thereby ensuring a better adequacy of micronutrient intake [23]. According to the authors, assigning positive scores for the nonconsumption of foods such as red meats could be an indirect indicator of inadequate micronutrient intake, which aligns with our results for the ELI index. The latter also employs a graded scoring system, but unlike the SHDI, it favors the nonconsumption of certain food groups. The proportional scoring indices, especially ELD-I and HRD, showed a positive, albeit weak, correlation with most PANDiet metrics (including, total PANDiet score, subscores, and nutrient adequacies). This is similar to a previous study showing that, despite a reduction in animal food consumption, the highest ELD-I quintiles had an increase in PANDiet score [20]. In contrast, indices with binary scoring were negatively associated with nutritional adequacy for several nutrients, supporting the need to establish minimum intake values to improve the accuracy of nutritional index measurements [65,66]. Regardless of the scoring system, an inverse relationship was observed between the EAT-Lancet indices and the nutritional adequacy of zinc and vitamin B-12, supporting previous findings [65,67]. Proportional indices demonstrated greater validity by correlating closely and more strongly with dietary quality indicators, such as the GDQS and cDQI (ρ < 0.69). These associations were expected, given that the indices promote the intake of healthy animal and plant foods, which are sources of antioxidant and anti-inflammatory compounds [[68], [69], [70]]. Overall, the results of these analyses support the association of the indices with a healthy diet, promoting nutritional adequacy and the consumption of antioxidant and anti-inflammatory compounds, with potential health benefits.

As for convergent validity related to environmental impact, stronger correlations were found for the ELD-I and ELI. Several studies support the positive impact of EAT-Lancet recommendations on the environment, such as significant reductions in GHGE and land use [18,71]. In addition, the food components that contribute most to the indices (for example, fruits and vegetables) are consistent with results on the effect of their increased intake on environmental aspects [72,73]. However, it is important to note that although these dietary patterns may have environmental benefits, the trade-off, such as increased water use [74], must be considered at the national and/or subnational level, given the water stress in a large number of countries [31,75]. Although the SHDI demonstrated strong performance with regard to nutritional health indicators, its association with environmental impact was weak, even correlating with increased environmental pressure. This highlights the need to consider both human health and environmental factors when designing planetary health metrics, aiming for an optimal balance between the 2 domains although taking the specific context into account.

Discrepancies in mean values of DI compared with the original studies suggest different consumption patterns according to population and geographic location. For instance, the WISH was lower in our study compared with the original [15], potentially reflecting variations in dietary habits between France and Vietnam. Furthermore, although the original study reported perfect scores for added sugars and saturated oils, in our context, fewer participants indicated not consuming these food components [15]. Although Vietnam has undergone a nutritional transition in recent decades, characterized by an increase in sugar and fat consumption, there is still evidence of lower consumption of these foods compared with France (46.5 g/day compared with 92.84 g/day and 8 g/day compared with 16 g/day, respectively) [[76], [77], [78]]. Moreover, the high compliance with the fruit and vegetable recommendations in the Brazilian validation study results in almost perfect scores for these groups [16], possibly because that study does not fully reflect the diet of the country. In a more recent evaluation, the authors applied the PHDI to a nationally representative survey from Brazil, finding that the mean scores in both groups are lower than those obtained in our study. This was expected, considering that fruit and vegetable consumption is higher in France than in Brazil (378 compared with 150 g/d, respectively) [79,80].

Regarding the ELD-I index, the pattern was similar to those of the study that developed the index, which was expected as samples are from the same geographical context [20]. In line with previous evidence, we observed a lack of variation in the unsaturated oils component, suggesting that the threshold based on the EAT-Lancet recommendations may not be appropriate for consumption levels in France [67]. These results indicate that the cut-off point established on the basis of the EAT-Lancet report exceeds the mean consumption level observed in France (around 8 g/d) according to the ELD-I criteria (≤80 g/d) [78].

On the other hand, the HRD pattern observed in this study closely aligns with that reported for other European countries in one recent preprint study published by the index's developers [19,81]. Similar to our findings, participants in the European Prospective Investigation into Cancer and Nutrition (EPIC) cohort exhibited high scores for dietary recommendations related to vegetables, fruits, tubers, and eggs, and low scores for added sugars, legumes, soy, and nuts. Specifically, greater similarities were noted with nations geographically proximate to France. For instance, scores related to dairy foods, red meat, and added sugars were comparable to those of Italy and Spain. In contrast, scores for poultry, added fats, and fish were more consistent with those reported for Germany, the United Kingdom, and the Netherlands. The scores for these food groups in Sweden and Denmark diverged significantly from the findings of this study. Finally, the total HRD score for the EPIC cohort was 64 points, higher than the 43 points observed in this study. This difference may be attributed to shifts in dietary habits over time, as the dietary data for the EPIC cohort were predominantly collected only at baseline stage in the 1990s [82]. Additionally, the use of a food frequency questionnaire in the EPIC cohort may have led to an overestimation of dietary intake data [82].

Regarding ELI index, we found a mean score similar to that of the Swedish study [21], although the food components with higher scores differed, possibly due to differences in consumption patterns between Sweden and France [83,84]. In this sense, the Swedish cohort used for the design of the ELI index reported a lower consumption of vegetables (< 200 g/d), and a higher consumption of potatoes (> 100 g/d) and fish (> 50 g/d) compared with the INCA3 [78,79]. The mean SHDI score in France was higher than that observed in the Gambia, likely due to the inherently low dietary diversity of Gambian diets [23,85]. This limited diversity partially explains the lower alignment with the healthy and sustainable dietary guidelines outlined by the EAT-Lancet Commission: overconsumption of a limited number of food groups, such as polished white rice, bread, oils, and added sugars, alongside the underconsumption of others [23].

Our findings revealed a mean DI score consistent with that reported in the original German cohort study, emphasizing similar challenges in adhering to recommendations for added sugars, nuts, fats, and pork [14]. Interestingly, the original study reported 100% compliance with the recommendations for legumes and soy foods, raising questions about the adequacy of the DI’s current thresholds (≤100 g/d and ≤50 g/d, respectively). In this sense, OECD/FAO data indicate that the mean consumption of dry legumes in the European Union between 2017 and 2019 was only 9.6 g/d, below the global mean of 21.1 g/d [86]. These data suggest that meeting the threshold for legumes and soy foods might not reflect sufficient dietary intake, calling for a critical reassessment of the scoring criteria.

Geographical divergences were also found for the other binary indices. For example, the mean score in the HSDI was twice as high in the original study conducted in Mexico [13]. This is because a significant proportion of the participants met the recommendations for several food components, such as tubers, unsaturated fats, fish, saturated fats, and beef, which aligns with Mexican dietary patterns [87]. Similarly, in the case of the ELDS index, differences were observed in the high compliance groups in the original UK sample compared with our sample in France [12]. It should be noted that in the original studies, there was no variability in the scores of the unsaturated fats in the HSDI and the dry beans, lentils, and peas in the ELDS, which was not replicated in our sample.

Overall, the differences in scores between the studies reflect variations in dietary consumption patterns in different regions and populations, emphasizing the importance of considering the context and specific characteristics when interpreting the dietary indices [88]. However, these findings confirm the sensitivity of the indices in capturing dietary cultural variability. Likewise, discrepancies in dietary intake could stem from variances in the intrinsic features of the study designs, such as the method employed for nutritional assessment (that is, 24-h dietary recall, food frequency questionnaires, or food diaries) and the number of days covered (that is, single or repeated measure).

These findings should be interpreted within the context of some limitations. Although sampling weights were applied to enhance representativeness, the cross-sectional design may limit the generalizability of the findings to contexts beyond France. Additionally, the estimation of predictive validity was constrained by the cross-sectional nature of the study, and assessing associations with health outcomes beyond anthropometry was not possible due to the absence of such data in the INCA3 survey. Future longitudinal studies are encouraged to analyze the link with noncommunicable diseases to strengthen validity research. Second, the manual disaggregation of complex dishes may introduce errors, so a continuous effort in the construction of composition tables and standardized recipes is necessary to accurately estimate the population intake in France. Nevertheless, it is crucial to acknowledge inherent limitations in the EAT-Lancet recommendations that may result in methodological inconsistencies. In this sense, the imprecision and lack of clarity regarding specific food groups within the EAT-Lancet diet can present challenges in its operationalization, especially in estimating the quantities of fats and added sugars, fostering uncertainty and personal interpretation [89,90]. Also, it would be useful to clearly define and standardize the quantification method for certain food components, such as whole grains or legumes, specifying whether intake should be reported in grams of cooked or dry weight. This would improve comparisons and prevent discrepancies arising from user interpretation. In this study, results are presented using grams of food intake; however, we confirmed that findings remain consistent when utilizing grams of dry weight, as expected due to the low intake of these food groups (unpublished data). Third, acknowledging limitations in the Agribalyse v.3.1.1 database is crucial, including the absence of soil carbon measurement in GHGE, information on biodiversity, phytosanitary product impact, and waste [91]. Additionally, incomplete water use inventory data highlights the need for considering spatial and temporal variability [92]. This demonstrates the continuing need for more comprehensive databases, incorporating various estimates related to food production methods, for accurate assessments of dietary sustainability in future research.

As the results of this study suggest, the measurement performance of indices assessing the adherence to the planetary health diet proposed by the EAT-Lancet Commission may vary, potentially impacting the reliability and validity of these indices. Therefore, it is essential to establish clear criteria for the contribution of each food component in the indices, including the number of components, scoring criteria, the use of adequate cut-off points, energy adjustment, and component weighting, aiming to enhance coherence among existing indices. To achieve this, we recommend following the framework provided by Waijers et al. [50] regarding key considerations in constructing a dietary index: *1*) it needs to have a clear objective, *2*) a rationale for the choice of index components, *3*) clear information on assigning foods to food groups, *4*) include an exact quantification of the index components against cut-off values, *5*) energy adjustment (or not), and *6*) information on the relative contribution of individual components to the total score.

Furthermore, we also consider essential that, with the launch of the EAT-Lancet diet 2.0 in 2025, a consensus should be reached on how to measure adherence to its recommendations. This will help to avoid an “overdevelopment” of indices, similar to what has happened with the dietary indices assessing the adherence to the Mediterranean diet [55,93]. An excessive proliferation of indices may cause challenges in terms of consistency and comparability between studies, making it difficult to identify a common standard for assessing adherence to the EAT-Lancet diet and further complicate the interpretation of research results and the implementation of these recommendations. Finally, it is crucial for future studies to explore the application of EAT-Lancet indices in vulnerable populations, including children, adolescents, and women of reproductive age [94]. This aligns with the growing interest in tailoring these indices to specific groups, each with unique physiological nutrient requirements [66,95,96]. These requirements encompass essential nutrients found in animal-based foods, fruits, vegetables, and legumes, highlighting the importance of considering the distinct nutritional needs of these populations when assessing their adherence to healthy and sustainable dietary patterns [94].

In conclusion, the different approaches to assess adherence to a sustainable and healthy diet are complementary, and the superiority of one method over another cannot be asserted. Thus, it is crucial to carefully address methodological issues to better understand the utility and applicability of these indices, including the precise clarification of objectives and assumptions, as well as a detailed description of score composition. In this regard, although indices like the ELD-I tend to reflect the healthiness and sustainability of the diet, others may be more valid for examining 1 of these 2 domains. For example, WISH is particularly effective as an indicator of diet adequacy. The choice of an index will depend on the specific needs of researchers. In practical terms, quantitative scoring indices are valuable tools in studies where precision and granularity are important such as clinical trials or epidemiological studies. Despite the associated cost of reduced variability and loss of statistical power, binary scoring indices find utility in surveys, observational studies, and public health interventions. Therefore, understanding the advantages and disadvantages of each index is relevant for interpreting the results of such investigations.

Given the ongoing development of new indices for assessing adherence to the EAT-Lancet recommendations, it is essential to conduct comprehensive assessment of the measures in terms of reproducibility, validity, and comparisons between different methodologies. This becomes even more crucial with the forthcoming publication of version 2.0 of the EAT-Lancet report in 2025, which is expected to address the main concerns identified in recent years.

## Author contribution

The authors’ responsibilities were as follows – ARM, EOV: conceptualized and designed the study, and drafted the manuscript; ARM, conducted the statistical analyses; FV, MM: provided essential materials (Agribalyse dataset) and critically reviewed the manuscript draft; EOV: supervised the project; ARM; EOV: held primary responsibility for the final content; and all authors: read and approved the final manuscript.

## Data availability

Data from the Third French Individual and National Food Consumption Survey (INCA3) are available on the data.gouv.fr platform. Data on the environmental impacts of foods consumed in France are available on the agribalyse.ademe.fr platform.

## Funding

This study was part of the FEAST (Food systems that support transitions to healthy and sustainable diets) project funded by the European Union's Horizon Europe research and innovation program under grant agreement number 101060536 and by Innovate UK under grant number 10041509. Swiss participant in FEAST was supported by the Swiss State Secretariat for Education, Research and Innovation under contract number 22.00156. More details at https://www.feast2030.eu/.

## Conflict of interest

The authors report no conflicts of interest.
