# Supplementary material for: How Do the Indices based on the EAT-Lancet Recommendations Measure Adherence to Healthy and Sustainable Diets? A Comparison of Measurement Performance in Adults from a French National Survey
Source: Curr Dev Nutr. 2025 Feb 7;9(3):104565. doi: 10.1016/j.cdnut.2025.104565 (PMC11919322; doi:10.1016/j.cdnut.2025.104565)
Supplement: Multimedia component 1 [file mmc1.pdf]

How do the indices based on the EAT-Lancet recommendations measure adherence to healthy and sustainable diets? A comparison of measurement performance in adults from a French national survey.

Miranda AR et al.

SUPPLEMENTARY MATERIAL

Supplementary Table 1. EAT-Lancet reference diet, with possible ranges, for an intake of 2,500 kcal/day 3
Supplementary Figure 1. Flowchart of sample selection process from the French Third Individual and National Study on Food Consumption Survey (INCA3). 4
World Index for Sustainability and Health 5
Supplementary Table 2. World Index for Sustainability and Health 6
Planetary Health Diet Index 7
Supplementary Table 3. Planetary Health Diet Index 8
EAT-Lancet diet index 9
Supplementary Table 4. EAT-Lancet diet index 9
Healthy Reference Diet score 10
Supplementary Table 5. HRD-score, intake recommendations (g/day) and scoring system for men based on 2,500 kcal/day 11
Supplementary Table 6. HRD-score, intake recommendations (g/day) and scoring system for women based on 2,000 kcal/day 12
EAT-Lancet Index 13
Supplementary Table 7. EAT-Lancet Index 13
Sustainable and Healthy Diet Index 14
Supplementary Table 8. Scoring system used to generate the SHDI. 15
Healthy and Sustainable Diet Index 16
Supplementary Table 9. Healthy and Sustainable Diet Index 16
EAT-Lancet Diet Score 17
Supplementary Table 10. EAT-Lancet Diet Score 17
Dietary Index 18
Supplementary Table 11. Dietary Index Score 19
PANDiet scoring system 20
Supplementary Table 12. PANDiet scoring system. 20
Global Diet Quality Score 21
Supplementary Table 13. Global Diet Quality Score 21
Comprehensive Diet Quality Index 23
Supplementary Table 14. Comprehensive Diet Quality Index 23

Dietary Inflammatory Index 24
Supplementary Table 15. Dietary Inflammatory Index 25
Composite Dietary Antioxidant Index 26
Supplementary Figure 2. Distribution plots. 27
Supplementary Table 16. Inter-item and Item-total correlation matrices of WISH 28
Supplementary Table 17. Inter-item and Item-total correlation matrices of PHDI 29
Supplementary Table 18. Inter-item and Item-total correlation matrices of ELD-I 30
Supplementary Table 19. Inter-item and Item-total correlation matrices of HRD 31
Supplementary Table 20. Inter-item and Item-total correlation matrices of ELI 32
Supplementary Table 21. Inter-item and Item-total correlation matrices of SHDI 33
Supplementary Table 22. Inter-item and Item-total correlation matrices of HSDI 34
Supplementary Table 23. Inter-item and Item-total correlation matrices of ELDS 35
Supplementary Table 24. Inter-item and Item-total correlation matrices of DI 36
Supplementary Figure 3. Internal consistency reliability of EAT-Lancet indices. 37
Supplementary Table 25. Correlation between indices and energy intake 38
Supplementary Figure 4. Percentile distribution of the of EAT-Lancet indices. 39
Supplementary Table 26. PANDiet score across quintiles of WISH 40
Supplementary Table 27. PANDiet score across quintiles of PHDI 41
Supplementary Table 28. PANDiet score across quintiles of ELD-I 42
Supplementary Table 29. PANDiet score across quintiles of HRD 43
Supplementary Table 30. PANDiet score across quintiles of ELI 44
Supplementary Table 31. PANDiet score across quintiles of SHDI 45
Supplementary Table 32. PANDiet score across quartiles of HSDI 46
Supplementary Table 33. PANDiet score across quartiles of ELDS 47
Supplementary Table 34. PANDiet score across quartiles of DI 48
Supplementary Table 35. Environmental impact across quintiles of WISH 49
Supplementary Table 36. Environmental impact across quintiles of PHDI 50
Supplementary Table 37. Environmental impact across quintiles of ELD-I 51
Supplementary Table 38. Environmental impact across quintiles of HRD 52
Supplementary Table 39. Environmental impact across quintiles of ELI 53
Supplementary Table 40. Environmental impact across quintiles of SHDI 54
Supplementary Table 41. Environmental impact across quartiles of HSDI 55
Supplementary Table 42. Environmental impact across quartiles of ELDS 56
Supplementary Table 43. Environmental impact across quartiles of DI 57
Supplementary Figure 5. Mean daily intake (g/day) of Eat-Lancet food components for participants in the highest and lowest quantiles of each dietary index. 58

Supplementary Table 1. EAT-Lancet reference diet, with possible ranges, for an intake of 2,500 kcal/day

Food components	Macronutrient intake (possible range), g/day	Caloric intake, kcal/day
**Whole grains**		
Rice, wheat, corn, and other†	232 (total gains 0–60% of energy)	811
**Tubers or starchy vegetables**		
Potatoes and cassava	50 (0–100)	39
**Vegetables**		
All vegetables	300 (200–600)	.
Dark green vegetables	100	23
Red and orange vegetables	100	30
Other vegetables	100	25
**Fruits**		
All fruit	200 (100–300)	126
**Dairy foods**		
Whole milk or derivative equivalents (eg, cheese)	250 (0–500)	153
**Protein sources**		
Beef and lamb	7 (0–14)	15
Pork	7 (0–14)	15
Chicken and other poultry	29 (0–58)	62
Eggs	13 (0–25)	19
Fish	28 (0–100)	40
Legumes		
Dry beans, lentils, and peas	50 (0–100)	172
Soy foods	25 (0–50)	112
Peanuts	25 (0–75)	142
Tree nuts	25	149
**Added fats**		
Palm oil	6.8 (0–6.8)	60
Unsaturated oils	40 (20–80)	354
Dairy fats (included in milk)	0	0
Lard or tallow	5 (0–5)	36
**Added sugars**		
All sweeteners	31 (0–31)	120

Willett W, et al. Food in the Anthropocene: the EAT-Lancet Commission on healthy diets from sustainable food systems. Lancet. 2019;393(10170):447-492.

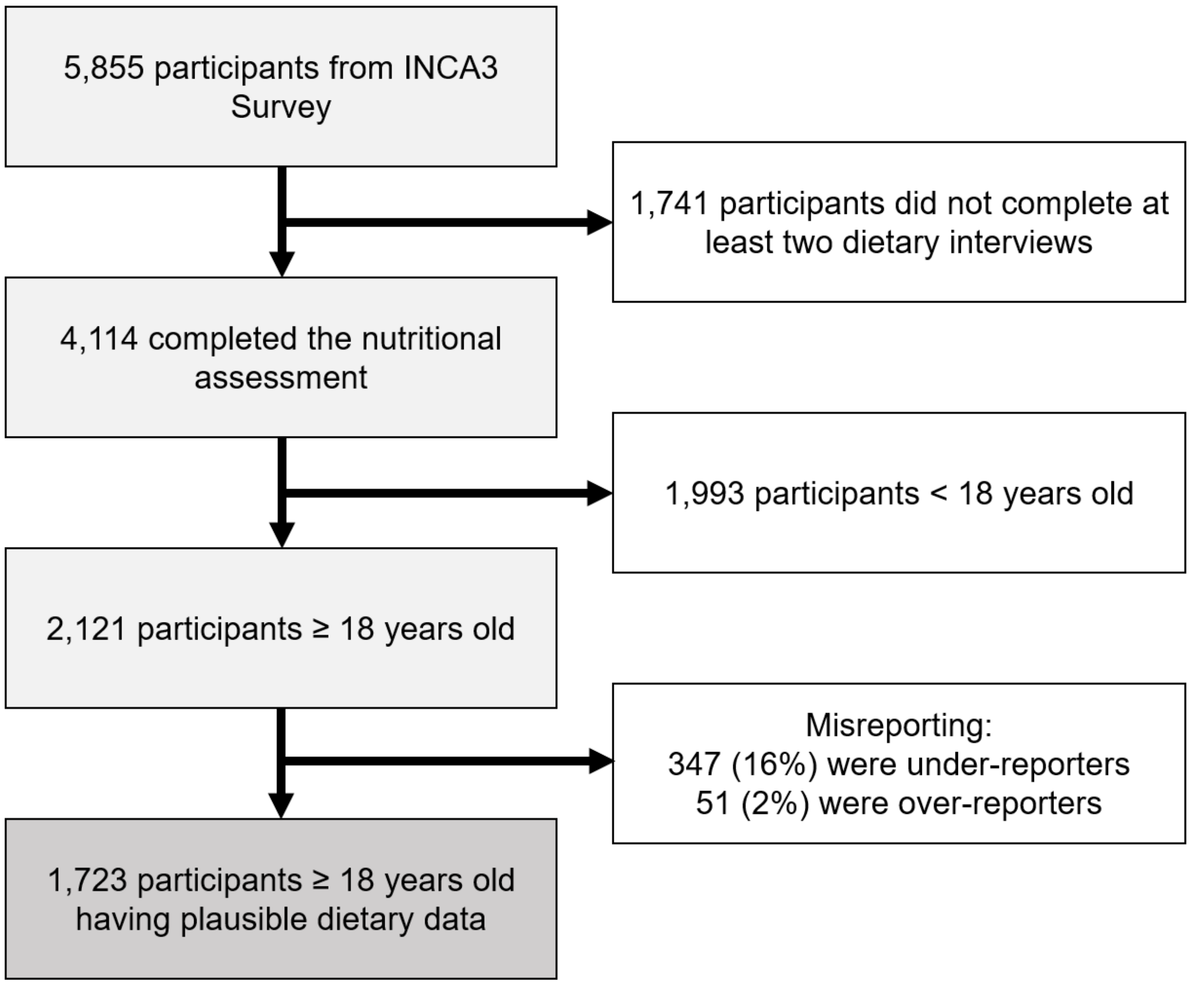

Supplementary Figure 1. Flowchart of sample selection process from the French Third Individual and National Study on Food Consumption Survey (INCA3).

World Index for Sustainability and Health

The World Index for Sustainability and Health (WISH) was developed to assess the healthiness and environmental sustainability of the diet based on EAT-Lancet recommendations. It comprises 13 food components classified as neutral, protective or negative for human and planetary health. Each food component is assessed by a quantitative score, using reference values in grams, which limits the independent individual assessment of total energy intake. The WISH index excludes tubers and starchy vegetables on the argument that they are not in the WHO and Global Burden of Disease study. It was developed using data collected through repeated 24-hour recall from a sample of 396 adults in Vietnam (urban area). The WISH score has been linked to certain health outcomes, such as decreased depression levels and improved cognitive performance.

The following formula is applied to compute the score of an intake between the lower recommended intake and the recommended intake:

$$WISH = 10 * \frac{reported\ intake - lower\ recommended\ intake}{recommended\ intake - lower\ recommended\ intake}$$

On the other hand, the score between the recommended intake and the upper recommended intake is calculated according to the following formula:

$$WISH = 10 * \frac{(upper\ recommended\ intake - recommended\ intake) - (reported\ intake - recommended\ intake)}{upper\ recommended\ intake - recommended\ intake}$$

Saturated oils and added sugars are scored as a bivariate component: 10 points are allocated for those consumptions within the recommended range and zero points are given for those exceeding this recommendation. The total score is calculated by adding the score of each food component, and ranges between 0 up to 130 points. Moreover, the authors propose four sub-scores derived from the WISH index: healthy, less healthy, low environmental impact and high environmental impact.

The following table presents the food components of WISH, proving examples of food items and a more detailed explanation of the scoring system.

Supplementary Table 2. World Index for Sustainability and Health		
**Food components**	**Food items**	**Scoring**
**Whole Grains**	Rice, wheat and corn (bran, germ, and endosperm in their natural proportion) or whole grain products in the form of breakfast cereals, bread, pasta, biscuits, muffins, tortillas, pancakes, and other sources.	≥125 g/d = 10 points.<br><100 g/d = 0 point.<br>100–125 g/d = 0–10 points.
**Vegetables**	Fresh, frozen, cooked, canned, or dried vegetables, and excludes legumes and salted or pickled vegetables, juices, nuts, and seeds, and starchy vegetables such as potatoes or corn.	≥300 g/d = 10 points.<br><200 g/d = 0 point.<br>200–300 g/d = 0–10 points.
**Fruits**	Fresh, frozen, cooked, canned, or dried fruits but excluding fruit juices, salted or pickled fruits and nuts or seeds.	≥200 g/d = 10 points.<br><100 g/d = 0 point.<br>100–200 g/d = 0–10 points.
**Dairy Foods**	Whole or skimmed milk or derivative equivalents (e.g., cheese, yoghurt or curd), excluding butter and cream.	250–500 g/d = 10 points.<br>0–250 g/d = 0–10 points.<br>>500 = 0 point.
**Red Meat**	Beef, pork, lamb, and goat including red meat in its processed form but excluding poultry, fish, eggs.	≤14 g/d = 10 points.<br>>28 g/d = 0 point.<br>14–28 g/d = 0–10 points, inversely.
**Fish**	Fish and shellfish such as mussels and shrimp.	28–100 g/d = 10 points.<br>0–28 g/d = 0–10 points.<br>>100 = 0 point.
**Eggs**	Eggs from chickens and ducks, but excludes fish eggs.	≤13 g/d = 10 points.<br>>25 g/d = 0 point.<br>13–25 g/d = 0–10 points, inversely.
**Chicken and Other Poultry**	Meat from chicken, ducks, geese etc.	≤29 g/d = 10 points.<br>>58 g/d = 0 point.<br>29–58 g/d = 0–10 points, inversely.
**Legumes**	Fresh, frozen, cooked, canned, or dried legumes, beans and lentils, but also includes soy foods and peas.	0-75 g/d = 0–10 points.<br>≥75 g/d = 10 points.
**Nuts**	Tree nuts and ground nuts (including peanuts).	50–75 g/d = 10 points.<br>0-50 g/d = 0–10 points.<br>≥75 g/d = 0 point.
**Unsaturated Oils**	Olive, soybean, rapeseed, sunflower and peanut oil.	≥40 g/d = 10 points.<br><20 g/d = 0 point.<br>20–40 g/d = 0–10 points.<br>> 80 g/d = 0 point.
**Saturated Oils**	Dairy fats, lard or tallows, and palm oil.	≤11.8 g/d= 10 points<br>> 11.8 g/d = 0 point.
**Added Sugars**	Added sugar and sugar sweetened beverages.	≤31 g/d= 10 points<br>> 31 g/d = 0 point.

Trijsburg L, Talsma EF, Crispim SP, Garrett J, Kennedy G, de Vries JHM, Brouwer ID. Method for the Development of WISH, a Globally Applicable Index for Healthy Diets from Sustainable Food Systems. Nutrients. 2020;13(1):93.

Planetary Health Diet Index

The Planetary Health Diet Index (PHDI) stands as one of the most widely recognized indices based on the reference dietary guidelines proposed by the EAT-Lancet Commission. Developed and validated using data from over 15,000 individuals of the ELSA-Brazil study, this index evaluates the caloric density of 16 food components through a continuous scoring system. The sample used for its development consisted in active and retired workers from six Brazilian public universities, aged 35 to 74, from three major regions collected between 2008 and 2010 that were assessed with FFQ. The PHDI covers all the food components proposed by EAT-Lancet, and the suggested ranges and midpoints for each component are calculated based on their energy contribution to a reference diet of 2,500 kcal/day. Using a gradual scoring, the PHDI allows components to receive scores based on the amount of intake. These scores are determined as a caloric intake ratio. For any component of the PHDI, the caloric intake ratio is defined as the sum of calories from all foods ranked in that component divided by the total calories from all foods covered by the PHDI.

As for the scoring, each of the 16 components can award a maximum of 10 or 5 points, resulting in a total score ranging from 0 to 150 points. This approach provides the ability to assess diet quality by considering various aspects, offering a quantitative measurement that encompasses various nutritional components and their intake levels. As a special feature, PHDI includes interchangeability between some food components.

The authors of the PHDI have presented validity and reliability indicators. In terms of internal reliability, it has been considered adequate, and the results of construct validity indicate that the PHDI presents a positive correlation with nutritional indicators, energy independency, and is structurally valid. In addition, the GHGE calculation was carried out to confirm its validity. Additionally, it exhibits an inverse association with various health indicators, such as obesity, blood pressure, total cholesterol, LDL cholesterol, and non-HDL cholesterol, although no significant association with HDL cholesterol, triglycerides or insulin resistance has been found.

The following table presents the food components of the PHDI, proving examples of food items and a more detailed explanation of the scoring.

Supplementary Table 3. Planetary Health Diet Index		
**Food components**	**Foods**	**Scoring**
**Nuts and peanuts**	Processed and raw nuts, pistachios, almonds, peanuts, and coconut pulp and milk	≥11.6% = 10 points.<br>0% = 0 point.<br>0–11.6% = 0–10 points.
**Legumes**	Dry or canned beans, pulses, lentils, chickpeas, peas, soybeans, and soy food products.	≥11.4% = 10 points.<br>0% = 0 point.<br>0–11.4 % = 0–10 points.
**Fruits**	Fresh and processed fruits, including culinary or industrial products such as fruit juices, nectars, and punches.	≥5% = 10 points.<br>0% = 0 point.<br>0–5% = 0–10 points.
**Total vegetables**	Fresh, frozen, cooked, canned, or dried vegetables and excluded legumes and starchy vegetables.	≥3.1% = 10 points.<br>0% = 0 point.<br>0–3.1% = 0–10 points.
**Whole grains**	Whole grains used as staple foods (e.g., brown rice, brown bread, oat flakes, canned grains, etc.), but not processed grains or refined products, such as polished rice and white bread.	≥32.4% = 10 points.<br>0% = 0 point.<br>0–32.4% = 0–10 points.
**Eggs**	Eggs from chickens and other poultry.	0–0.8% = 0–10 points.<br>0.8–1.5% = 0–10 point, inversely.<br>>1.5% = 0 point.
**Fish and seafood**	Fish and seafood.	0–1.6% = 0–10 points.<br>1.6–3.1% = 0–10 point, inversely.<br>>3.1% = 0 point.
**Dairy**	Cow's milk, goat's milk, buffalo products, yogurt, and cheese, but no butter and sour cream.	0–6.1% = 0–10 points.<br>6.1–12.2% = 0–10 point, inversely.<br>>12.2% = 0 point.
**Vegetable oils**	Vegetable oils.	0–16.5% = 0–10 points.<br>16.5–30.7% = 0–10 point, inversely.<br>>30.7% = 0 point.
**Dark green vegetables to total vegetables ratio**	All dark green vegetables were included, such as broccoli and rocket, but not light green vegetables, such as lettuce.	0–29.5% = 0–5 points.<br>≥29.5% = 0–5 point, inversely.
**Red and orange to total vegetables ratio**	Tomatoes, beets, carrots, and pumpkins are examples of foods included in this component.	0–38.5% = 0–5 points.<br>≥38.5% = 0–5 point, inversely.
**Red meats**	Beef, lamb, and pork. Processed beef and pork (for example, sausages, ham, bologna, dried meat) were also accounted.	0% = 10 points.<br>0–2.4% = 0–10 point, inversely.<br>>2.4% = 0 point.
**Chicken and substitutes**	Chickens, poultry, and their substitutes (i.e., eggs and fish and seafood). Processed poultry meat (e.g., smoked brisket and nuggets, smoked breast, and pate).	0% = 10 points.<br>0–5% = 0–10 point, inversely.<br>>5% = 0 point.
**Animal fats**	Dairy fats (Butter and creams, including sour cream and cheese cream), lard, and tallow.	0% = 10 points.<br>0–1.4% = 0–10 point, inversely.<br>>1.4% = 0 point.
**Added sugars**	All sweeteners, including white or brown sugars and honey used as ingredients in processed or culinary products and the added sugars to manufactured foods and beverages.	0% = 10 points.<br>0–4.8% = 0–10 point, inversely.<br>>4.8% = 0 point.

Cacau LT, De Carli E, de Carvalho AM, Lotufo PA, Moreno LA, Bensenor IM, Marchioni DM. Development and Validation of an Index Based on EAT-Lancet Recommendations: The Planetary Health Diet Index. Nutrients. 2021;13(5):1698.

EAT-Lancet diet index

Data from 29,210 French adults (75% women) who participated in the NutriNet-Santé study were used to developed the EAT-Lancet Dietary Index (ELD-I). The ELD-I comprehensively covers 14 food components using cut-off points adapted from those proposed in the EPIC (European Prospective Investigation into Cancer and Nutrition)-Oxford study. The ELD-I is based on a quantitative scoring system that was designed to more accurately capture the variability in food consumption patterns, incorporating individual's total energy intake and standardizing it to a daily intake of 2,500 kcal. The computation results in a continuous variable being either positive or negative. As the score increases, the individual's diet aligns more closely with the EAT-Lancet recommendations. Moreover, the validity of the ELD-I has been explored, with results confirming its ability to measure healthiness and sustainability of diets.

The following table presents the food components of the ELD-I, proving examples of food items and a more detailed explanation of the scoring.

Supplementary Table 4. EAT-Lancet diet index

Food components	Food items	Scoring computation
**Whole grains**	Rice, wheat, corn, and other.	$ELD-I_i = \frac{100\times\left\{\sum_{component\ i=1}^{14}\frac{\alpha_i\times\left(cut-off_i-\frac{consumption_{ij}\times 2500}{Energy\ intake\ _j}\right)}{cut-off_i}\right\}}{14}$<br>Where *i* referred to on the 14 food groups and j is the individual. $\alpha_i = 1$ for component to limit and $\alpha_i = -1$ for component to promote.
**Potatoes and tuber**	Potatoes and cassava.	
**Vegetables**	All non-starchy vegetables.	
**Fruits**	All fruits.	
**Dairy foods**	Whole milk or derivative equivalents (e.g., cheese).	
**Beef, lamb, pork**	Red meats of beef, lamb and pork and processed meats.	
**Chicken and poultry**	Chicken and other poultry (e.g., turkey, duck, and quail).	
**Eggs**	Eggs from chicken, duck, and goose.	
**Fish**	Fish and shellfish (e.g., mussels and shrimps).	
**Legumes**	Dry beans, lentils, peas, soy foods.	
**Nuts**	Almonds, hazelnuts, pistachios and walnuts.	
**Saturated oil**	Dairy fats (e.g., cream and butter), lard or tallow.	
**Unsaturated oils**	Olive, soybean, rapeseed, sunflower, and peanut oil.	
**All sweet**	Added sugars and sugar-sweetened beverages.	

Kesse-Guyot E, Rebouillat P, Brunin J, Langevin B, Allès B, Touvier M, et al. Environmental and nutritional analysis of the EAT-Lancet diet at the individual level: insights from the NutriNet-Santé study. J Clean Prod. 2021;296:126555.

Healthy Reference Diet score

The Healthy Reference Diet (HRD) score was developed using data from 35,496 adults in the Dutch EPIC-NL cohort, which combines the MORGEN cohort (men and women aged 20–64 from Amsterdam, Doetinchem, and Maastricht) and the Prospect cohort (women aged 49–70 in a breast screening program in Utrecht). Participants, recruited between 1993 and 1997, completed a food frequency questionnaire to assess their habitual intake of 178 food items over the previous year.

To design HRD scoring system, dietary recommendations from the EAT-Lancet report were adjusted based on individuals' observed intake and recalculated for a 2,000 kcal/day diet for women, as recommended by Dutch dietary guidelines. This resulted in sex-specific thresholds for 14 food components (Table S5). Participants were given proportional scores from 0 to 10 for each recommendation, with the total score ranging from 0 (no adherence) to 140 (complete adherence). The HRD score categorized food groups into four components: adequacy, moderation, optimum, and ratio, based on their associations with chronic disease, as informed by relevant research. The authors used the energy-adjusted nutrient residual model to account for variations in total energy consumption.

The HRD was validated by linking participant data to national morbidity and mortality registries, and environmental impact data was obtained from life cycle assessments. The development study found that high adherence to the HRD was linked to lower risks of cardiovascular disease, coronary heart disease, and stroke. Additionally, high HRD adherence was associated with reduced greenhouse gas emissions, land use, freshwater eutrophication, marine eutrophication, and terrestrial acidification, as well as higher blue water use.

Colizzi C, Harbers MC, Vellinga RE, Verschuren WMM, Boer JMA, Biesbroek S, et al. Adherence to the EAT-Lancet Healthy Reference Diet in Relation to Risk of Cardiovascular Events and Environmental Impact: Results From the EPIC-NL Cohort. J Am Heart Assoc. 2023;12(8):e026318.

Supplementary Table 5. HRD-score, intake recommendations (g/day) and scoring system for men based on 2,500 kcal/day

Food Group	Component type*	HRD recommendation (g/day)	Minimum score (0 points)	Proportional score	Maximum points (10 points)	Proportional score
**Whole grains**†						
Rice, wheat, corn, and other	A	464 (or 60%E)	0 g/d	0-464 g/d	≥464 g/d	
**Vegetables**						
All vegetables‡	A	300	0 g/d	0-300 g/d	≥300 g/d	
**Fruits**						
All fruit§	A	200	0 g/d	0-200 g/d	≥200 g/d	
**Tubers or starchy vegetables**						
Potatoes and cassava	O	50	0 g/d	0-50 g/d	50-100 g/d	100-150 g/d
**Dairy foods**						
Whole milk or derivative equivalents (e.g., cheese)	O	250	0 g/d	0-250 g/d	250 – 500 g/d	500-750 g/d
**Protein sources**						
Dry beans, lentils, and peas	A	50	0 g/d	0-50 g/d	≥50 g/d	
Soy foods	A	25	0 g/d	0-25 g/d	≥25 g/d	
Beef, lamb and pork	M	14	≥14 g/d	14 -0 g/d	0 g/d	
Chicken and other poultry	O	29	0 g/d	0-29 g/d	29-58 g/d	58-88 g/d
Eggs	O	13	0 g/d	0-13 g/d	13-25 g/d	25-38 g/d
Fish	O	28	0 g/d	0-28 g/d	28-100 g/d	100-128 g/d
Nuts	O	50	0 g/d	0-50 g/d	50-100 g/d	100-150 g/d
**Added sugars**						
All sweeteners	M	31	≥31 g/d	31-0 g/d	0 g/d	
**Added fats**	R					
Palm oil		6.8	No consumption of unsaturated fats OR ratio of unsaturated to saturated fats of ≤0.6 ‖		No consumption of saturated fats OR ratio of unsaturated to saturated fats of ≥13 ‖	
Unsaturated oils		40				
dairy fats included in milk		0				
lard and tallow		5				

A = adequacy component; O = optimum component; M = moderation component; R = ratio component. †Reference diet refers to dry, raw weight. ‡ Including dark green vegetables, red and orange vegetables, other vegetables. § Excluding fruit juice. ‖ Cut-offs and threshold values were derived from the 15th percentile and 85th percentile of the intake distribution of the Dutch reference population.

Supplementary Table 6. HRD-score, intake recommendations (g/day) and scoring system for women based on 2,000 kcal/day

Food Group	Component type*	HRD recommendation (g/day)	Minimum score (0 points)	Proportional score	Maximum points (10 points)	Proportional score
**Whole grains**†						
Rice, wheat, corn, and other	A	372 (or 60%en)	0 g/d	0-372 g/d	≥372 g/d	
**Vegetables**						
All vegetables‡	A	240	0 g/d	0-240 g/d	≥240 g/d	
**Fruits**						
All fruit§	A	160	0 g/d	0-160 g/d	≥160 g/d	
**Tubers or starchy vegetables**						
Potatoes and cassava	O	40	0 g/d	0-40 g/d	40-80 g/d	80-120 g/d
**Dairy foods**						
Whole milk or derivative equivalents (e.g., cheese)	O	200	0 g/d	0-200 g/d	200 – 400 g/d	400-600 g/d
**Protein sources**						
Dry beans, lentils, and peas	A	40	0 g/d	0-40 g/d	≥40 g/d	
Soy foods	A	20	0 g/d	0-20 g/d	≥20 g/d	
Beef, lamb and pork	M	12	≥12 g/d	12-0 g/d	0 g/d	
Chicken and other poultry	O	23	0 g/d	0-23 g/d	23-46 g/d	46-69 g/d
Eggs	O	10	0 g/d	0-10 g/d	10-20 g/d	20-30 g/d
Fish	O	22	0 g/d	0-22 g/d	22-80 g/d	80-102 g/d
Nuts	O	40	0 g/d	0-40 g/d	40-80 g/d	80-120 g/d
**Added sugars**						
All sweeteners	M	25	≥25 g/d	25-0 g/d	0 g/d	
**Added fats**	R					
Palm oil		5	No consumption of unsaturated fats OR ratio of unsaturated to saturated fats of ≤0.5 ‖		No consumption of saturated fats OR ratio of unsaturated to saturated fats of ≥11 ‖	
Unsaturated oils		32				
dairy fats included in milk		0				
lard and tallow		4				

A = adequacy component; O = optimum component; M = moderation component; R = ratio component. †Reference diet refers to dry, raw weight. ‡ Including dark green vegetables, red and orange vegetables, other vegetables. § Excluding fruit juice. ‖ Cut-offs and threshold values were derived from the 15th percentile and 85th percentile of the intake distribution of the Dutch reference population.

EAT-Lancet Index

The EAT-Lancet Index (ELI) was developed using data from 22,421 Swedish adults aged 45 to 73 who participated in the Malmö Diet and Cancer cohort between 1991 and 1996, which included food diaries and food frequency questionnaires. The ELI covers 14 food components categorized as emphasized and restricted foods, with daily gram quantities assessed through a semi-quantitative scoring system: from 0 to 3 points. This index has been related with reduced all-cause mortality, decreased mortality from cancer and cardiovascular diseases. Furthermore, it was observed that higher adherence to this index is associated with a lower risk of type 2 diabetes in Swedish adults, and this relationship is dose-response and independent of genetic susceptibility.

The following table presents the food components of the ELI, proving examples of food items and a more detailed explanation of the scoring.

Supplementary Table 7. EAT-Lancet Index

Food components	Food items	Scoring			
		3 points	2 points	1 point	0 points
**Vegetables**	All vegetables except legumes.	>300	200–300	100–200	<100
**Fruits**	Fruits and berries.	>200	100–200	50–100	<50
**Unsaturated oils**	All plant oils and plant margarines.	>40	20–40	10–20	<10
**Legumes**	Dry beans, lentils, peas, soy. Peas, lentils, beans, tofu, soy containing meat replacement products.	>75	37.5–75	18.75–37.5	<18.75
**Nuts**	Peanuts or tree nuts. All nuts and seeds including peanuts, nut mixes such as almond paste.	>50	25–50	12.5–25	<12.5
**Whole grains**	Whole grains (e.g., cereals, rolled oats, crispbread) and whole grain foods (e.g., pastas, doughs and breads).	>232	116–232	58–116	<58
**Fish**	Fatty fish, lean fish, fish products, shellfish.	>28	14–28	7–14	<7
**Beef and lamb**	Beef, lamb, minced meat with pork and lamb, processed meats with beef and lamb including sausages.	<7	7–14	14–28	>28
**Pork**	Pork, minced meat of pork, processed meats with pork including ham, bacon, and sausages.	<7	7–14	14–28	>28
**Poultry**	Chicken, turkey, duck, goose, and other poultry.	<29	29–58	58–116	>116
**Eggs**	Boiled eggs, fried eggs and eggs in dishes such as omelet and pie.	<13	13–25	25–50	>50
**Dairy**	Whole milk or derivative equivalents. Regular milk, low-fat milk, yoghurt and other fermented milk products, hard cheese, soft cheese, cream, butter, butter-based spreads. All dairy foods were expressed as of milk equivalents. Equivalency factor: whole milk 1.0, Cheese 5.0, cream 2.7 and butter 6.5.	<250	250–500	500–1000	>1000
**Potatoes**	Boiled potatoes, fried potatoes, deep fried potatoes, potatoes included in dishes such as potato salad.	<50	50–100	100–200	>200
**Added sugar**	Sucrose and monosaccharides except sugars in fruits and vegetable.	<31	31–62	62–124	>124

Stubbendorff A, Sonestedt E, Ramne S, Drake I, Hallström E, Ericson U. Development of an EAT-Lancet index and its relation to mortality in a Swedish population. Am J Clin Nutr. 2022;115(3):705-716.

Sustainable and Healthy Diet Index

The Sustainable and Healthy Diet Index (SHDI) was developed using data from the Integrated Household Survey (IHS2015/16) of The Gambia, conducted between May 2015 and April 2016. This comprehensive, nationally representative cross-sectional survey collected year-round dietary data from 12,713 households to account for seasonal variations in food consumption. Dietary information was gathered through a 7-day recall questionnaire, which included 145 food items. The SHDI assesses adherence to the EAT-Lancet diet by combining two scoring methods to reflect the Gambian food system, nutritional needs, and eating patterns. Method 1 scores food group consumption based on micronutrient adequacy, assigning points for intakes within the EAT-Lancet range, but avoids positive scores for non-consumed essential food groups. In food-insecure areas like The Gambia, non-consumption of certain foods is considered a proxy for micronutrient inadequacy, so the lower bound of the intake range is adjusted to the mean. Method 2 reflects the risk of non-communicable diseases and mortality by assigning scores based on consumption levels of food groups, awarding higher scores for beneficial foods (e.g., vegetables, fruits, whole grains) and deducting points for harmful foods (e.g., beef, pork, potatoes, added sugar). The SHDI combines these methods, using 0-3-point system from Method 2 while incorporating micronutrient adequacy from Method 1, and adjusting the intake ranges for certain foods. The final SHDI score, based on 16 food groups, ranges from 0 to 48, with higher scores indicating better adherence to the EAT-Lancet diet (Table S8). The association with nutritional health and environmental indicators was not analyzed in the index development study.

Supplementary Table 8. Scoring system used to generate the SHDI.

EAT-Lancet food group	EAT-Lancet target intake (range in grams d−1)	Intake range with minimum intake values (g d−1)[a]	Score[b]			
			3 points	2 points	1 point	0 points
All vegetables	300 (200–600)	200–600	>300	200–300	100–200	<100
All fruits	200 (100–300)	100–300	>200	100–200	50–100	<50
Unsaturated oils	40 (20–80)	20–80	>40	20–40	10–20	<10
Beans, lentils and peas	75 (0–150)	75–150	>75	37.5–75	18.75–37.5	<18.75
Peanuts and tree nuts	50 (0–100)	50–100	>50	25–50	12.5–25	<12.5
Wholegrains	116 (0–232)	116–232	>116	58–116	29–58	<29
Potatoes and cassava	50 (0–100)	50–100	>50	25–50	12.5–25	<12.5
Fish	28 (0–100)	28–100	>28	14–28	7–14	<7
Palm oil	6.8 (0–6.8)	⩽6.8	<1.7	1.7–3.4	3.4–6.8	>6.8
Added sugar	31 (0–31)	15.5–31	<7.75	7.75–15.5	15.5–31	>31
Refined grains	116 (0–232)	116–232	<116	58–116	116–232	>232
Beef and lamb	7 (0–14)	7–14	7–14	3.5–7	1.75–3.5	<1.75 or >14
Pork	7 (0–14)	7–14	7–14	3.5–7	1.75–3.5	<1.75 or >14
Poultry	29 (0–58)	29–58	29–58	14.5–29	7.25–14.5	<7.25 or >58
Dairy	250 (0–500)	250–500	250–500	125–250	62.5–125	<62.5 or >500
Eggs	13 (0–25)	13–25	13–25	6.5–13	3.25–6.5	<3.25 or >25

A= Based on Method 1 except for refined grains and palm oil where we allowed positive scoring for non-consumption. Our approach treats beef & lamb and pork as two groups and splits grains into wholegrains and refined grains. B = Scores were assigned based on Method 2 with exceptions to: wholegrains, beef and lamb, pork, poultry, dairy, and potatoes and cassava where we used different criteria and avoided awarding points for non-consumption. We awarded points differently for added sugar intake by taking points away for intakes above the upper limit while Method 2 assigned positive scores for intakes up to 200% of the upper limit. Grains (whole and refined), beans, lentils and peas are dry, raw and includes soy foods consistent with EAT-Lancet recommendations. EAT-Lancet recommendations for grains are combined with target 232 g (0–464 g). These were split in this report to reflect local availability and consumption patterns.

Ali Z, Scheelbeek PFD, Felix J, Jallow B, Palazzo A, Segnon AC, Havlík P, Prentice AM, Green R. Adherence to EAT-Lancet dietary recommendations for health and sustainability in the Gambia. Environ Res Lett. 2022;17(10):104043.

Healthy and Sustainable Diet Index

The Healthy and Sustainable Diet Index (HSDI), introduced by Shamah-Levy et al. in 2020, is based on national representative FFQ-derived data from Mexico in 2018-2019 (n = 11,506). The HSDI assesses 13 food components with a binary scoring system assigning one point for each component meeting recommended energy percentages. The authors found a negative relationship between HSDI and obesity in men, although no association was shown in women.

The following table presents the food components of the HSDI, proving examples of food items and a more detailed explanation of the scoring.

Supplementary Table 9. Healthy and Sustainable Diet Index

Food components	Food items	Scoring	
		1 point	0 points
**Whole grains**	E.g., whole wheat bread, corn dough.	≥32.44	<32.44
**Tubers or starchy vegetables**	Potato and sweet potato	≤1.56	>1.56
**Vegetables**	All vegetables (e.g., turnip, tomato, carrot, zucchini, broccoli, cauliflower, green beans, cabbage, lettuce, cucumber, onion, pepper and mushrooms, etc.).	≥3.12	<3.12
**Fruits**	All fruits (e.g., orange, apple, pear, banana, melon, watermelon, mango, pineapple, grapefruit, strawberry, grape, peach, plum, etc.).	≥5.02	<5.02
**Milk and dairy**	Milk, cheese and yoghurt of all types.	≤6.12	>6.12
**Beef and pork**	Beef or pork, all types of cuts and viscera.	≤0.64	>0.64
**Chicken and other poultry**	Any type of poultry and viscera.	≤2.48	>2.48
**Eggs**	Egg.	≤1.00	>1.00
**Fish and seafood**	Fresh fish, dried fish, tuna, sardine, any seafood.	≤1.60	>1.60
**Legumes, soybeans and tree nuts**	Beans, lentils, soymilk, chickpeas, peanuts.	≥23.0	<23.0
**Unsaturated fats**	Oil from corn, sunflower, sesame, olive.	≥14.16	<4.16
**Saturated fats**	Cream, butter, milk fat, lard, bacon.	≤3.84	>3.84
**Added sugars**	Sweeteners added to beverages, soft drinks, natural or industrialized juices and nectars, candies, sugars in desserts, cookies or cakes, chocolates, etc.	≤5.00	>5.00

Shamah-Levy T, Gaona-Pineda EB, Mundo-Rosas V, Méndez Gómez-Humarán I, Rodríguez-Ramírez S. Asociación de un índice de dieta saludable y sostenible con sobrepeso y obesidad en adultos mexicanos [Association of a healthy and sustainable dietary index and overweight and obesity in Mexican adults]. Salud Publica Mex. 2020;62(6):745-753.

EAT-Lancet Diet Score

The EAT-Lancet Diet Score (ELDS) was the first measure derived from the EAT-Lancet report. The recommended amount of energy intake is 2,500 kcal per day, similar to the EAT- Lancet reference diet. For each food group, an intake threshold was established. The ELDS was developed using data from Food Frequency Questionnaire of 46,069 participants enrolled in the European Prospective Investigation into Cancer and Nutrition (EPIC)-Oxford study between 1993 and 2001. The ELDS assesses 14 food components using a binary scoring system: one point if the recommendation is met, otherwise zero point is allocated. The ELDS has been linked to ischemic heart disease and diabetes, although it does not show a clear relationship with mortality or stroke.

The following table presents the food components of the ELDS, proving examples of food items and a more detailed explanation of the scoring.

Supplementary Table 10. EAT-Lancet Diet Score

Food components	Food items	Scoring	
		1 point	0 points
**Whole grains**	Rice, wheat, corn, and other.	≤464	>464
**Tubers and starchy vegetables**	Potatoes and cassava.	≤100	>100
**Vegetables**	All non-starchy vegetables.	≥200	<200
**Fruits**	All fruit.	≥100	<100
**Dairy foods**	Whole milk or derivative equivalents (e.g., cheese).	≤500	>500
**Beef, lamb, pork**	Red meats of beef, lamb and pork and processed meats.	≤28	>28
**Chicken, other poultry**	Chicken and other poultry (e.g., turkey, duck, and quail).	≤58	>58
**Eggs**	Eggs from chicken, duck, and goose.	≤25	>25
**Fish**	Fish and shellfish (e.g., mussels and shrimps).	≤100	>100
**Dry beans, lentils, peas**	Dry beans, lentils, peas.	≥100	<100
**Peanuts or tree nuts**	Almonds, hazelnuts, pistachios and walnuts.	≥25	<25
**Added fats**	Dairy fats (e.g., cream and butter), lard or tallow.	0.8	0.8
**Added sugar**	Added sugars and sugar-sweetened beverages.	≤31	>31
**Soy foods**	Soy foods (e.g., soy milk, tofu)	≤50	>50

Knuppel A, Papier K, Key TJ, Travis RC. EAT-Lancet score and major health outcomes: the EPIC-Oxford study. The Lancet. 2019;394(10194):213-4.

Dietary Index

The Dietary Index (DI) was developed by comparing dietary data from 2–5 sets of 3-day weighed dietary records of participants (n = 298; ≥15 years) in the DONALD (Dortmund Nutritional and Anthropometric Longitudinal Designed) study with the Eat-Lancet reference diet. The records included detailed information on food and drink intake, recipes, and convenience foods, categorized into 18 food components based on the Eat-Lancet diet. Adjustments were made for whole grains, peanuts, and tree nuts, with all grains considered and a minimum intake of whole grains emphasized.

Each food component was standardized to 2,500 kcal/day (by multiplying the food component by 2,500 kcal and dividing the product by the individual energy intake), aligning with the Eat-Lancet reference diet. Participants received 1 point for food component intakes meeting recommendations and 0 points otherwise, resulting in DI scores ranging from 0 (low adherence) to 18 (high adherence).

The DI was assessed for associations with nutritional characteristics, ecological sustainability, and risk markers (anthropometric and cardiometabolic) in young adults (≥18 years). Higher DI scores were linked to lower intakes of protein, added sugars, and cholesterol but higher fiber intake. Additionally, higher DI scores were associated with reduced greenhouse gas emissions and land use, as well as inverse relationships with anthropometric markers in young adulthood. No associations with cardiometabolic risk markers were found.

Montejano Vallejo R, Schulz CA, van de Locht K, Oluwagbemigun K, Alexy U, Nöthlings U. Associations of Adherence to a Dietary Index Based on the EAT-Lancet Reference Diet with Nutritional, Anthropometric, and Ecological Sustainability Parameters: Results from the German DONALD Cohort Study. J Nutr. 2022 Jul 6;152(7):1763-1772.

Supplementary Table 11. Dietary Index Score		
**Food components**	**Criteria for scoring 1 point[1]**	**Food items included in each food component**
**Whole grains & all grains[2]**	≤ 464 g/d and whole grain fiber[3]	Flour, mixed flour, ready to eat cereals, muesli, bread, grain, cooked grain, sweet bread, dough, pasta.
**Tubers or starchy vegetables**	≤ 100 g/d	Potatoes and potatoes products.
**Vegetables**	≥ 200 - ≤ 600 g/d	Vegetables and mushrooms fresh, frozen, canned, and dried.
**Fruits**	≥ 100 - ≤ 300 g/d	Fruit fresh, frozen, canned, and dried.
**Dairy foods**	≤ 500 g/d	Dairy products unfermented and fermented, fresh cheese, semi hard cheese, soft cheese, processed cheese, diary powder.
**Protein sources**		
**Beef and lamb**	≤ 14 g/d	Beef, lamb, veal, and other meats (goat, horse), offal, sausages, meat dishes.
**Pork**	≤ 14 g/d	Pork, offal, sausages, meat dishes.
**Chicken & other poultry**	≤ 58 g/d	Poultry, offal, sausages.
**Eggs**	≤ 25 g/d	Eggs.
**Fish[4]**	≤ 100 g/d	Fish fresh or frozen, processed fish.
**Dry beans, lentils&peas[2]**	≤ 100 g/d	Pulses and pulses flour.
**Soy foods**	≤ 50 g/d	Soy beans, soy flour, vegan milk substitute, vegetarian/vegan spread, meat or dish, egg substitute.
**All nuts[5]**	≥ 25 g/d	Nuts and seeds, nut spreads.
**Added fats**		
**Palm oil**	≤ 6.8 g/d	Palm oil and palm fat.
**Unsaturated oils**	≥ 20 - ≤ 80 g/d	Vegetable oils and margarines.
**Lard or tallow**	≤ 5 g/d	Animal fat or oils.
**Butter**	0 g/d	Milk fat (butter).
**All sweeteners**	≤ 31 g/d	Sugar and sweeteners, sweet parfait, candy, chocolate bar, ice cream and water ice.

[1] Scoring is based on the EAT-Lancet reference diet, for an intake of 2,500 kcal/d, with minor adjustments.
[2] Whole grain and beans, lentils and peas as dry, raw weight.
[3] Considering a minimum intake of ≥5g/d of fiber from all grains and ≥0g/d from whole grain from included food items.
[4] Includes fish and shellfish.
[5] Includes tree- and peanuts.
Abbreviation: d=day; DI=Dietary Index; g=grams.

PANDiet scoring system

The PANDiet is a measure of individual diet quality expressed as a 100-point score that determines the probability that overall nutrient intake is adequate [1]. The PANDiet is calculated as the mean of two sub-scores: The Adequacy Subscore (i.e., the probability of that intake to be adequate), and the Moderation Subscore (i.e., the probability of not exceeding intake of nutrients that should be limited). The total PANDiet score ranges from 0 to 100, with a higher score indicating a better nutritional quality of the diet. The following table presents the PANDiet scoring system.

Supplementary Table 12. PANDiet scoring system.

Adequacy subscore			
**Nutrient**	**RV (/day)**	**CV**	**Source**
**Protein**	0.66 or 0.8 g/Kg bw	12.5%	[2]
**LA**	3.08% EIEA	15%	[2]
**ALA**	0.769%	15%	[2]
**DHA**	0.192 g	15%	[2]
**EPA+DHA**	0.385 g	15%	[2]
**Fiber**	23 g	15%	[2]
**Vitamin A**	580 or 490 µg	15%	[2]
**Thiamin**	0.3 mg/1000 kcal	20%	[2]
**Riboflavin**	1.3 mg	15%	[2]
**Niacin**	5.44 mg NE/1000 kcal	10%	[2]
**Pantothenic acid**	3.33 or 2.78 mg	40%	[1]
**Vitamin B-6**	1.5 or 1.3 mg	10%	[2]
**Folate**	250 µg	15%	[2]
**Vitamin B-12**	3.33 µg	10%	[2]
**Vitamin C**	90 mg	10%	[2]
**Vitamin D**	10 µg	25%	[2]
**Vitamin E**	5.26 or 4.74 mg	40%	[2]
**Iodine**	107 µg	20%	[2]
**Magnesium**	224 or 176 mg	35%	[2]
**Phosphorus**	Calcium (mmol)/1.65	7.5%+CV calcium	[2]
**Potassium**	2692 mg	15%	[2]
**Selenium**	54 µg	15%	[2]
**Zinc**	Phytate (mg/d) / Zinc (mg) M / F: 300 / 9.4 / 7.5; 600 / 11.7 / 9.3; 900 / 14.0 / 11.0; 1200 / 16.3 / 12.7	10%	[3]
**Copper**	0.86 or 0.68 mg	40%	[2]
**Manganese**	1.89 or 1.56 mg	40%	[2]
**Calcium**	860 (≤ 24 yo.) or 750 (>24 yo.)	15% or 13%	[2]
**Iron**	6 or 7 mg	20%	[4]

Moderation subscore			
**Nutrient**	**RV (/day)**	**CV**	**Source**
**Protein**	2.2 g/kg bw	12.5%	[2]
**Carbohydrates**	60.5% EIEA	5%	[2]
**Total fat**	44% EIEA	5%	[2]
**SFA**	12% EIEA	15%	[2]
**Sugars**	100 g	15%	[2]
**Sodium**	3312 or 2618 mg	40%	[2]

Tolerable Upper Intake Limits		Source
**Vitamin A (retinol only)**	3000 µg	[2]
**Niacin**	900 mg	[2]
**Vitamin B6**	25 mg	[2]
**Folate**	1170 µg	[2]
**Vitamin D**	100 µg	[2]
**Vitamin E**	300 mg	[2]
**Calcium**	2500 mg	[2]
**Copper**	10 mg	[2]
**Iodine**	600 µg	[2]
**Dissociable magnesium**	250 mg	[2]
**Selenium**	300 µg	[2]
**Zinc**	25 mg	[2]

Computation [1]

$$F\left(\frac{\bar{y}-r}{\sqrt{SD_r^2+SD_y^2/n}}\right)$$

F is the "normal" function in Stata
$\bar{y}$ is the mean intake
SD^2_y is the day-to-day variability of intake
n is the number of days of dietary data
r is the nutrient reference value
SD^2_r is the interindividual variability

Note. DHA and EPA+DHA are weighted by a factor of 1/2 as DHA is present twice. Niacin equivalents are calculated as the sum of dietary niacin and 1/60 dietary tryptophan. ALA, alpha-linolenic acid; bw, body weight; CV, coefficient of variation; DHA, docosahexaenoic acid; EIEA, energy intake excluding alcohol; EPA, eicosapentaenoic acid; LA, linoleic acid; NE, niacin equivalent; SFA, saturated fatty acids.

[1] Verger EO, Mariotti F, Holmes BA, Paineau D, Huneau JF. Evaluation of a Diet Quality Index Based on the Probability of Adequate Nutrient Intake (PANDiet) Using National French and US Dietary Surveys. PLoS ONE. 2012;7(8).
[2] Salomé M, Mariotti F, Nicaud MC, Dussiot A, Kesse-Guyot E, Maillard MN, Huneau JF, Fouillet H. Eropean Journal of Nutrition. 2022;61(4):1991-2002.
[3] EFSA (2014). Draft Scientific Opinion on Dietary Reference Values for Zinc. EFSA Journal 2014. Available online: https://www.efsa.europa.eu/sites/default/files/consultation/140514%2C0.pdf. Accessed 30 Nov 2023.
[4] ANSES (2021). Les références nutritionnelles en vitamines et minéraux. In: Anses—Agence nationale de sécurité sanitaire de l'alimentation, de l'environnement et du travail. Available online: https://www.anses.fr/fr/content/les-r%C3%A9f%C3%A9rences-nutritionnelles-en-vitamines-et-min%C3%A9raux. Accessed 30 Nov 2023.

Global Diet Quality Score

The Global Diet Quality Score (GDQS) is a diet quality assessment tool focusing on 25 food groups considered critical for nutrient intake and/or risk of non-communicable diseases. The score is composed of 16 food groups categorized as healthy (scored positively to encourage higher intake), seven groups classified as unhealthy (scored negatively to penalize higher levels of intake), and two food groups that are considered unhealthy only if consumed in excessive amounts (score 0 if intake is low or excessive). The total score ranges from 0 to 49. Also, two additional GDQS-related sub-metrics are calculated: the GDQS+ (only for food groups considered healthy), which ranges from 0 to 32; and the GDQS- (only for food groups considered unhealthy or harmful in excessive amounts), which ranges from 0 to 17. The following table presents the GDQS scoring system.

Supplementary Table 13. Global Diet Quality Score

**Food group**	**Food items**	**Categories of consumption**				**Point values**			
		**1**	**2**	**3**	**4**	**1**	**2**	**3**	**4**
GDQS+									
Healthy									
Citrus fruits	Fruits in the genus Citrus	<24	24–69	>69		0	1	2	
Deep orange fruits	Fruits containing ≥20 retinol equivalents/100 g	<25	25–123	>123		0	1	2	
Other fruits	Fruits not belonging in the other fruit categories	<27	27–107	>107		0	1	2	
Dark green leafy vegetables	Leafy vegetables containing >120 retinol equivalents/100 g	<13	13–37	>37		0	2	4	
Cruciferous vegetables	Vegetables in the family Brassicaceae	<13	13–36	>36		0	0.25	0.5	
Deep orange vegetables	Non-tuberous vegetables containing ≥120 retinol equivalents/100 g	<9	9–45	>45		0	0.25	0.5	
Other vegetables	Vegetables not belonging in the other vegetable categories	<23	23–114	>114		0	0.25	0.5	
Legumes	Legumes and foods derived from legumes.	<9	9–42	>42		0	2	4	
Deep orange tubers	Tuberous vegetables containing ≥120 retinol equivalents/100 g	<12	12–63	>63		0	0.25	0.5	
Nuts and seeds	Nuts, seeds, and products derived	<7	7–13	>13		0	2	4	
Whole grains	Whole grains and whole-grain products.	<8	8–13	>13		0	1	2	
Liquid oils	All types of oils that are liquid at room temperature.	<2	2–7.5	>7.5		0	1	2	
Fish and shellfish	Fish (processed or unprocessed) and seafood (e.g., shellfish).	<14	14–71	>71		0	1	2	
Poultry and game meat	Unprocessed poultry and game.	<16	16–44	>44		0	1	2	
Low fat dairy	Reduced or naturally low-fat dairy products (≤2% milk fat).	<33	33–132	>132		0	1	2	
Eggs	All types of eggs. Does not include mayonnaise	<6	6–32	>32		0	1	2	

Food group	Food items	Categories of consumption				Point values			
		**1**	**2**	**3**	**4**	**1**	**2**	**3**	**4**
GDQS-									
Unhealthy in excessive amounts									
High fat dairy	High fat milk and dairy products (>2% milk fat), except butter, ice cream and whipped cream.	<35	35–142	>142–734	>734	0	1	2	0
Red meat	Unprocessed red meat belonging to domesticated animals (i.e., not game), including organs.	<9	9–46	>46		0	1	0	
Unhealthy									
Processed meat	Processed red meat, poultry, or game, including organs.	<9	9–30	>30		2	1	0	
Refined grains and baked goods	Refined grains and refined grain products.	<7	7–33	>33		2	1	0	
Sweets and ice cream	Sugar-sweetened foods that are not beverages (e.g., sugar, sweeteners, whipped cream).	<13	13–37	>37		2	1	0	
Sugar-sweetened beverages	Sweetened drinks that do not contain any fruit juice (e.g., sodas, energy drinks, sports drinks).	<57	57–180	>180		2	1	0	
Juice	Unsweetened or sweetened drinks that are at least partly composed of fruit juice.	<36	36–144	>144		2	1	0	
White roots and tubers	Tuberous vegetables with <120 retinol equivalents/100 g.	<27	27–107	>107		2	1	0	
Purchased deep fried foods	Deep fried foods fried in an amount of fat or oil sufficient to cover the food.	<9	9–45	>45		2	1	0	

Bromage S, Batis C, Bhupathiraju SN, Fawzi WW, Fung TT, Li Y, Deitchler M, Angulo E, Birk N, Castellanos-Gutiérrez A, He Y, Fang Y, Matsuzaki M, Zhang Y, Moursi M, Gicevic S, Holmes MD, Isanaka S, Kinra S, Sachs SE, Stampfer MJ, Stern D, Willett WC. Development and Validation of a Novel Food-Based Global Diet Quality Score (GDQS). J Nutr. 2021;151(12 Suppl 2):75S-92S.

Comprehensive Diet Quality Index

The Comprehensive Diet Quality Index (cDQI) is a recently developed measure that ranges from 0 to 85 and is obtained by adding two sub-indexes: the plant-based diet quality index (pDQI) and the animal-based diet quality index (aDQI). The main purpose of this metric is to distinguish between the consumption of plant and animal products that are considered healthy and those that are not. The cDQI aims to provide a comprehensive assessment of diet quality, considering both plant and animal components. The two sub-indices are calculated using thresholds based on literature evidence or crude consumption quintiles. The following table presents the cDQI scoring system.

Supplementary Table 14. Comprehensive Diet Quality Index

		Max score (5)	Min Score (0)
**Plant-based Diet Quality Index (pDQI)**			
Healthful			
	Wholegrain products	≥ 45g per 1000/kcal	No consumption
	Vegetables (excluding potatoes)	≥ 125g per 1000/kcal	No consumption
	Fruit	≥ 125g per 1000/kcal	No consumption
	Nuts, seeds, legumes	≥ 14,175g per 1000/kcal	No consumption
	Vegetable oils	≥ 14.86g per 1000/kcal	< 4.13g per 1000/kcal
	Coffee, tea	≥477.35g per 1000/kcal	< 91.72g per 1000/kcal
Unhealthy			
	Fruit juices	No consumption	≥79.38g per 1000/kcal
	Refined grains	<54g per 1000/kcal	≥129g per 1000/kcal
	Potatoes	No consumption	≥ 35g per 1000/kcal
	Sugar-sweetened beverages	No consumption	≥ 226.8g per 1000/kcal
	Sweets and desserts	< 14.98g per 1000/kcal	≥ 40.32g per 1000/kcal
Range pDQI		**0 to 55**	
**Animal-based Diet Quality Index (aDQI)**			
Healthful			
	Fish, seafood	≥ 14,175g per 1000/kcal	No consumption
	Dairy	≥ 312g per 1000/kcal	No consumption
	Poultry	≥ 17.09g per 1000/kcal	< 3.12g per 1000/kcal
Unhealthy			
	Processed meat	No consumption	≥ 28,35g per 1000/kcal
	Red meat	No consumption	≥ 45,36g per 1000/kcal
	Eggs	< 1.78g per 1000/kcal	≥ 8.62g per 1000/kcal
Range aDQI		**0 to 30**	
**cDQI Total Range**		**0 to 85**	

Brunin J, Allès B, Péneau S, Reuzé A, Pointereau P, Touvier M, et al. Do individual sustainable food purchase motives translate into an individual shift towards a more sustainable diet? A longitudinal analysis in the NutriNet-Santé cohort. Clean Responsib Consum. (2022) 5:100062.

Dietary Inflammatory Index

The Dietary Inflammatory Index (DII) is a population-based index derived from the literature for the purpose of measuring the overall impact of diet on inflammatory potential. It is based on the individual inflammatory effects of up to 45 dietary parameters. Unlike single compound or single food approaches, a strategic advantage of the DII is that it allows the study of the dietary matrix and the complex interactions of nutrients and compounds present in foods, as well as overall dietary patterns.

The specific steps for the calculation were as follows:

1) dietary intake data were compared with the global standard dietary intake database, and a Z-score was calculated for each nutrient based on the mean and standard deviation of that nutrient intake:

$$Z\ score = \frac{individual\ reported\ intake - global\ daily\ mean\ intake}{global\ standard\ deviation}$$

2) the Z-scores were converted into centered proportions,
3) the centered proportion for each dietary parameter was multiplied by the specific inflammatory effect score to obtain a DII score,
4) DII scores were summed for all dietary parameters to obtain the total DII score.

Since its release, the DII index has undergone rigorous validations in both cross-sectional and longitudinal studies. These validations have yielded consistent results indicating that higher DII scores are linked to proinflammatory dietary patterns. In addition, a significant association has been observed between elevated DII scores and elevated plasma concentrations of proinflammatory cytokines over time and health outcomes.

The following table presents the DII scoring system for the 34 parameters used in the current study.

Supplementary Table 15. Dietary Inflammatory Index

Food parameter	Overall inflammatory effect score	Global daily mean intake (units/d)	SD
Alcohol (g)	−0.278	13.98	3.72
Vitamin B12 (μg)	0.106	5.15	2.70
Vitamin B6 (mg)	−0.365	1.47	0.74
β-Carotene (μg)	−0.584	3718	1720
Carbohydrate (g)	0.097	272.2	40.0
Energy (kcal)	0.180	2056	338
Total fat (g)	0.298	71.4	19.4
Fiber (g)	−0.663	18.8	4.9
Folic acid (μg)	−0.190	273.0	70.7
Garlic (g)	−0.412	4.35	2.90
Ginger (g)	−0.453	59.0	63.2
Fe (mg)	0.032	13.35	3.71
Mg (mg)	−0.484	310.1	139.4
MUFA (g)	−0.009	27.0	6.1
Niacin (mg)	−0.246	25.90	11.77
n-3 Fatty acids (g)	−0.436	1.06	1.06
n-6 Fatty acids (g)	−0.159	10.80	7.50
Onion (g)	−0.301	35.9	18.4
Protein (g)	0.021	79.4	13.9
PUFA (g)	−0.337	13.88	3.76
Riboflavin (mg)	−0.068	1.70	0.79
Saffron (g)	−0.140	0.37	1.78
Saturated fat (g)	0.373	28.6	8.0
Se (μg)	−0.191	67.0	25.1
Thiamin (mg)	−0.098	1.70	0.66
Turmeric (mg)	−0.785	533.6	754.3
Vitamin A (RE)	−0.401	983.9	518.6
Vitamin C (mg)	−0.424	118.2	43.46
Vitamin D (μg)	−0.446	6.26	2.21
Vitamin E (mg)	−0.419	8.73	1.49
Zn (mg)	−0.313	9.84	2.19
Pepper (g)	−0.131	10.00	7.07
Thyme/oregano (mg)	−0.102	0.33	0.99
Rosemary (mg)	−0.013	1.00	15.00

Shivappa N, Steck SE, Hurley TG, Hussey JR, Hébert JR. Designing and developing a literature-derived, population-based dietary inflammatory index. Public Health Nutr. 2014;17(8):1689-96.

Composite Dietary Antioxidant Index

The Composite Dietary Antioxidant Index (CDAI) is a comprehensive measure that assesses an individual's antioxidant profile by combining several dietary antioxidants, such as vitamins A, C and E, manganese, selenium and zinc. Its development was based on the cumulative effect of anti-inflammatory indicators related to various health problems mediated by oxidative processes.

Widely used to evaluate the antioxidant capacity of dietary components, the CDAI is calculated from the levels of vitamins A, C and E, as well as manganese, selenium and zinc. The formula used to calculate the CDAI has been previously validated and is expressed as follows:

$$CDAI = \sum_{i=1}^{n=6} \frac{Total\ nutrient\ intake - mean}{SD}$$

Here, the total nutrient intake reflects the daily intake of antioxidant for each individual; the mean represents the mean of the antioxidant in the whole sample, while the standard deviation (SD) indicates the variability of the antioxidant in the whole sample.

Wang L, Yi Z. Association of the Composite dietary antioxidant index with all-cause and cardiovascular mortality: A prospective cohort study. Front Cardiovasc Med. 2022;9:993930.

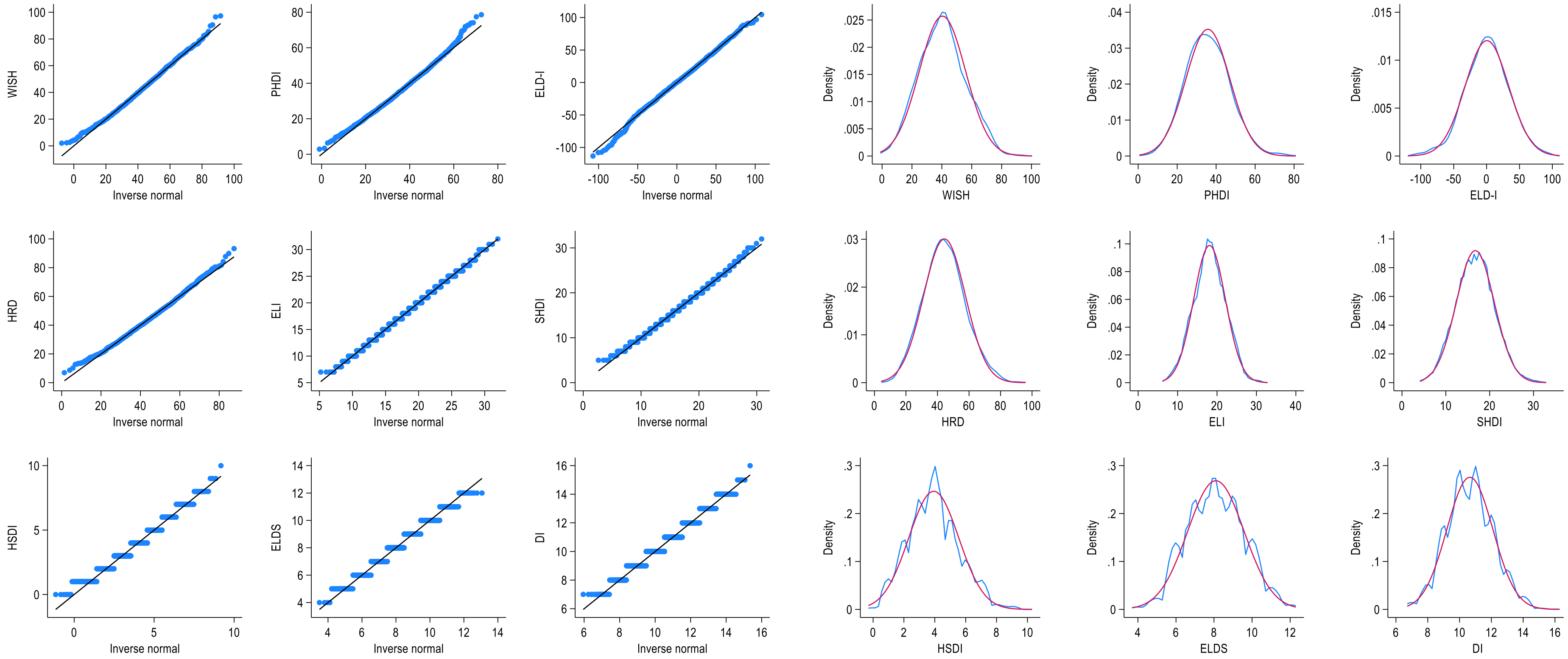

Supplementary Figure 2. Distribution plots. QQ-plots (left panel) and Kernel density estimates (right panel) of normal densities.

Supplementary Table 16. Inter-item and Item-total correlation matrices of WISH

Food components	1	2	3	4	5	6	7	8	9	10	11	12	13	14
1. Whole grains	1	0.0818	0.3581	0.0014	0.0583	0.6530	0.1643	0.3422	0.0386	0.2030	0.0171	0.4462	0.6574	0.0000
2. Fruits	0.04	1.00	0.1659	0.0131	0.3477	0.0039	0.8658	0.0029	0.0526	0.0592	0.4150	0.0001	0.0000	0.0000
3. Dairy foods	0.02	0.03	1	0.5167	0.0158	0.5356	0.0029	0.0212	0.2383	0.0012	0.2458	0.0141	0.0039	0.0000
4. Nuts	0.08	0.06	0.02	1	0.7977	0.5229	0.0469	0.1525	0.0000	0.0000	0.8216	0.0106	0.0008	0.0000
5. Unsaturated oils	0.05	0.02	-0.06	0.01	1	0.0000	0.0760	0.5966	0.3201	0.0024	0.0830	0.0606	0.0476	0.0000
6. Saturated oils	-0.01	0.07	0.01	0.02	0.10	1	0.0000	0.9292	0.0035	0.1365	0.0067	0.0000	0.0003	0.0000
7. Eggs	-0.03	0.00	-0.07	-0.05	0.04	0.13	1	0.0608	0.3842	0.9739	0.0000	0.0000	0.9804	0.0000
8. Chicken and other poultry	-0.02	0.07	0.06	0.03	-0.01	0.00	-0.05	1	0.0000	0.5026	0.6145	0.3269	0.3428	0.0000
9. Red meat	0.05	0.05	0.03	0.15	-0.02	0.07	-0.02	-0.16	1	0.0057	0.0008	0.1584	0.0084	0.0000
10. Fish	0.03	0.05	0.08	0.14	-0.07	0.04	0.00	0.02	0.07	1	0.5568	0.0495	0.1199	0.0000
11. Legumes	0.06	-0.02	0.03	-0.01	-0.04	-0.07	-0.10	0.01	-0.08	0.01	1	0.0009	0.0952	0.0001
12. Added sugars	0.02	0.10	-0.06	-0.06	0.05	0.22	0.13	0.02	0.03	0.05	-0.08	1	0.0035	0.0000
13. Vegetables	0.01	0.32	0.07	0.08	0.05	0.09	0.00	0.02	0.06	0.04	0.04	0.07	1	0.0000
14. WISH total score	0.13	0.49	0.25	0.21	0.15	0.45	0.32	0.28	0.23	0.38	0.10	0.46	0.50	1

Note. Pearson correlation coefficients are presented in the lower hemimatrix and p-values in the upper hemimatrix.

Supplementary Table 17. Inter-item and Item-total correlation matrices of PHDI

Food components	1	2	3	4	5	6	7	8	9	10	11	12	13	14	15	16	17
1. Nuts and peanuts	1	0.3615	0.1406	0.0056	0.0485	0.9254	0.0048	0.8601	0.0261	0.3357	0.0000	0.3099	0.9768	0.4560	0.1164	0.0414	0.0000
2. Legumes	-0.02	1	0.1227	0.333	0.6061	0.6075	0.0909	0.0000	0.4562	0.7133	0.0407	0.6528	0.0097	0.0094	0.2480	0.4666	0.0001
3. Fruits	0.04	-0.04	1	0.0000	0.0146	0.3298	0.1798	0.8030	0.6500	0.0289	0.9164	0.0222	0.0529	0.0000	0.0000	0.0000	0.0000
4. Vegetables	0.07	-0.02	0.20	1	0.5190	0.9709	0.6802	0.1185	0.1556	0.6184	0.5198	0.0947	0.2761	0.0024	0.0000	0.0000	0.0000
5. Whole cereals	0.05	0.01	0.06	0.02	1	0.1390	0.2186	0.0135	0.9390	0.0009	0.0924	0.2256	0.5275	0.0063	0.0853	0.8937	0.0000
6. Eggs	0.00	-0.01	-0.02	0.00	0.04	1	0.9100	0.6957	0.5961	0.7777	0.0048	0.3979	0.0006	0.0001	0.9610	0.0049	0.0000
7. Fish and seafood	0.07	0.04	-0.03	-0.01	0.03	0.00	1	0.0840	0.0323	0.0987	0.0473	0.0436	0.8617	0.0617	0.0387	0.3588	0.0000
8. Tubers and potatoes	0.00	0.13	-0.01	-0.04	0.06	0.01	0.04	1	0.6357	0.3182	0.0949	0.4500	0.1117	0.0435	0.9336	0.0065	0.0000
9. Dairy	0.05	-0.02	-0.01	0.03	0.00	-0.01	-0.05	-0.01	1	0.0000	0.5167	0.5375	0.0260	0.2156	0.0640	0.0001	0.0000
10. Vegetable oils	0.02	-0.01	-0.05	-0.01	-0.08	-0.01	0.04	0.02	0.14	1	0.9178	0.8021	0.0000	0.4762	0.0071	0.1809	0.0000
11. Read meat	0.11	-0.05	0.00	0.02	0.04	-0.07	0.05	-0.04	-0.02	0.00	1	0.1815	0.2534	0.6370	0.2636	0.1135	0.0000
12. Chicken and substitutes	-0.01	-0.02	0.01	0.01	-0.07	-0.02	0.15	0.05	0.04	-0.01	-0.04	1	0.5156	0.2033	0.1195	0.0851	0.0000
13. Animal fats	0.00	-0.06	0.05	0.03	-0.02	-0.08	0.00	-0.04	-0.05	0.11	0.03	0.06	1	0.0000	0.0012	0.4424	0.0000
14. Added sugars	-0.02	-0.06	0.13	0.07	0.07	-0.10	-0.05	-0.05	-0.03	-0.02	0.01	-0.02	0.24	1	0.0001	0.0682	0.0000
15. DGV/total ratio	0.04	0.03	0.18	0.26	0.04	0.00	0.05	0.00	-0.04	0.06	0.03	-0.02	0.08	0.09	1	0.0000	0.0000
16. ReV/total ratio	0.05	-0.02	0.19	0.28	0.00	-0.07	0.02	0.07	0.09	0.03	0.04	0.04	-0.02	0.04	0.37	1	0.0000
17. PHDI total score	0.31	0.09	0.45	0.40	0.16	0.25	0.31	0.19	0.24	0.29	0.19	0.36	0.26	0.25	0.39	0.39	1

Note. Pearson correlation coefficients are presented in the lower hemimatrix and p-values in the upper hemimatrix.

Supplementary Table 18. Inter-item and Item-total correlation matrices of ELD-I

**Food components**	**1**	**2**	**3**	**4**	**5**	**6**	**7**	**8**	**9**	**10**	**11**	**12**	**13**	**14**	**15**
1. Whole grains	1	0.0076	0.0022	0.7599	0.0126	0.0001	0.4525	0.9719	0.7839	0.1071	0.1346	0.0000	0.0067	0.0218	0.0039
2. Potatoes and tuber	-0.06	1	0.0000	0.5783	0.2539	0.3237	0.1697	0.0000	0.2202	0.4633	0.0005	0.5976	0.0106	0.0001	0.0000
3. Beef, lamb, pork	-0.07	0.17	1	0.0000	0.0158	0.0000	0.0521	0.0236	0.0000	0.0000	0.3693	0.0093	0.0000	0.0006	0.0000
4. Chicken and poultry	-0.01	-0.01	-0.24	1	0.0065	0.2236	0.1018	0.2311	0.4604	0.1805	0.0216	0.6033	0.0544	0.5399	0.9611
5. Eggs	0.06	-0.03	-0.06	-0.07	1	0.5770	0.9339	0.1711	0.0001	0.1126	0.0224	0.9693	0.4950	0.1672	0.0000
6. Fish	0.09	-0.02	-0.15	0.03	-0.01	1	0.4230	0.9769	0.0161	0.0000	0.0017	0.0000	0.1051	0.0729	0.0001
7. Legumes	0.02	-0.03	0.05	-0.04	0.00	0.02	1	0.0986	0.0773	0.2153	0.8974	0.2922	0.5831	0.8579	0.0005
8. Unsaturated oils	0.00	0.24	0.05	-0.03	0.03	0.00	-0.04	1	0.0000	0.0000	0.0083	0.0182	0.5577	0.0019	0.3204
9. Saturated oils	0.01	-0.03	-0.13	0.02	0.10	-0.06	0.04	-0.16	1	0.0000	0.0000	0.0000	0.0148	0.0000	0.0000
10. Added sugars	-0.04	-0.02	-0.15	-0.03	0.04	-0.12	-0.03	-0.10	0.12	1	0.0000	0.0000	0.7890	0.0108	0.0000
11. Vegetables	-0.04	0.08	0.02	-0.06	-0.06	-0.08	0.00	-0.06	0.13	0.17	1	0.0000	0.0480	0.0000	0.0000
12. Fruits	-0.12	-0.01	0.06	-0.01	0.00	-0.12	0.03	-0.06	0.17	0.17	0.34	1	0.0364	0.0070	0.0000
13. Nuts	-0.07	0.06	0.16	0.05	-0.02	-0.04	0.01	-0.01	0.06	-0.01	0.05	0.05	1	0.5351	0.0000
14. Dairy foods	0.06	-0.10	-0.08	-0.01	-0.03	0.04	0.00	-0.07	-0.13	-0.06	-0.12	-0.07	0.02	1	0.0000
15. ELDI total score	-0.07	0.27	0.45	0.00	0.28	-0.09	0.08	-0.02	0.46	0.33	0.49	0.58	0.24	-0.11	1

Note. Pearson correlation coefficients are presented in the lower hemimatrix and p-values in the upper hemimatrix.

Supplementary Table 19. Inter-item and Item-total correlation matrices of HRD

Food components	1	2	3	4	5	6	7	8	9	10	11	12	13	14	15
1. Whole grains	1	0.300	0.000	0.707	0.101	0.001	0.000	0.178	0.686	0.033	0.190	0.000	0.255	0.740	0.000
2. Vegetables	0.03	1	0.000	0.000	0.341	0.035	0.068	0.079	0.771	0.072	0.012	0.000	0.815	0.728	0.000
3. Fruits	0.11	0.33	1	0.000	0.640	0.354	0.034	0.981	0.544	0.856	0.078	0.019	0.000	0.008	0.000
4. Tubers or starchy vegetables	0.01	-0.09	-0.09	1	0.353	0.212	0.031	0.000	0.344	0.039	0.039	0.697	0.023	0.001	0.000
5. Dairy foods	-0.04	-0.02	-0.01	-0.02	1	0.932	0.932	0.001	0.161	0.034	0.253	0.376	0.009	0.000	0.000
6. Dry beans, lentils, and peas	0.08	0.05	-0.02	0.03	0.00	1	0.706	0.045	0.024	0.104	0.641	0.621	0.000	0.012	0.000
7. Soy foods	0.11	0.04	0.05	-0.05	0.00	0.01	1	0.004	0.137	0.334	0.135	0.021	0.020	0.219	0.000
8. Beef, lamb and pork	0.03	0.04	0.00	-0.10	-0.08	-0.05	0.07	1	0.005	0.259	0.051	0.000	0.450	0.046	0.000
9. Chicken and other poultry	-0.01	-0.01	0.01	0.02	0.03	0.05	0.04	-0.07	1	0.087	0.379	0.907	0.096	0.763	0.000
10. Eggs	-0.05	-0.04	0.00	0.05	-0.05	-0.04	-0.02	-0.03	0.04	1	0.303	0.011	0.000	0.001	0.000
11. Fish	0.03	0.06	0.04	0.05	0.03	0.01	0.04	0.05	-0.02	-0.02	1	0.000	0.319	0.370	0.000
12. Nuts	0.09	0.11	0.06	0.01	-0.02	0.01	0.06	0.11	0.00	-0.06	0.13	1	0.115	0.724	0.000
13. Added sugars	0.03	0.01	0.11	-0.05	-0.06	-0.09	0.06	0.02	0.04	0.09	0.02	-0.04	1	0.000	0.000
14. Added fats	-0.01	0.01	0.06	-0.08	-0.11	-0.06	0.03	0.05	0.01	0.08	-0.02	-0.01	0.23	1	0.000
15. HRD total score	0.14	0.37	0.42	0.28	0.22	0.23	0.22	0.12	0.37	0.33	0.43	0.24	0.28	0.20	1

Note. Pearson correlation coefficients are presented in the lower hemimatrix and p-values in the upper hemimatrix.

Supplementary Table 20. Inter-item and Item-total correlation matrices of ELI

**Food components**	**1**	**2**	**3**	**4**	**5**	**6**	**7**	**8**	**9**	**10**	**11**	**12**	**13**	**14**	**15**
1. Vegetables	1	0.0000	0.0978	0.0530	0.0017	0.6018	0.0551	0.0072	0.0157	0.2384	0.6921	0.0000	0.0581	0.0001	0.0000
2. Fruits	0.34	1	0.1818	0.8000	0.0100	0.0000	0.0100	0.9900	0.1300	0.0200	0.7300	0.0000	0.0600	0.0000	0.0000
3. Unsaturated oils	0.04	0.03	1	0.7597	0.0124	0.1152	0.1199	0.0001	0.1946	0.3910	0.1208	0.3230	0.0000	0.7137	0.0000
4. Legumes	0.05	-0.01	0.01	1	0.7742	0.0010	0.1231	0.0062	0.0485	0.8431	0.0022	0.0083	0.7134	0.0140	0.0000
5. Nuts	0.08	0.06	0.06	0.01	1	0.0117	0.0000	0.0000	0.0363	0.9987	0.0018	0.6073	0.4267	0.0007	0.0000
6. Whole grains	0.01	0.09	0.04	0.08	0.06	1	0.0187	0.0304	0.6571	0.6165	0.2615	0.0255	0.2065	0.5681	0.0000
7. Fish	0.05	0.06	-0.04	0.04	0.1	0.06	1	0.0000	0.0000	0.3231	0.9068	0.0442	0.2104	0.0052	0.0000
8. Beef and lamb	0.06	0.00	-0.09	-0.07	0.12	0.05	0.11	1	0.3824	0.0000	0.9774	0.0349	0.0000	0.7478	0.0000
9. Pork	0.06	0.04	0.03	-0.05	0.05	0.01	0.11	-0.02	1	0.0011	0.4074	0.0059	0.8425	0.0129	0.0000
10. Poultry	0.03	0.06	0.02	0.00	0.00	-0.01	0.02	-0.14	-0.08	1	0.0727	0.0027	0.3663	0.1829	0.0000
11. Eggs	-0.01	0.01	-0.04	-0.07	-0.08	-0.03	0.00	0.00	-0.02	-0.04	1	0.3634	0.6756	0.0000	0.0000
12. Dairy	-0.10	-0.12	0.02	-0.06	-0.01	-0.05	-0.05	-0.05	0.07	-0.07	0.02	1	0.1400	0.0007	0.0000
13. Potatoes	0.05	-0.04	-0.19	0.01	-0.02	0.03	0.03	0.11	0.00	-0.02	-0.01	-0.04	1	0.0650	0.0000
14. Added sugar	0.09	0.08	-0.01	-0.06	-0.08	-0.01	0.07	0.01	0.06	0.03	0.17	0.08	0.04	1	0.0000
15. ELI total score	0.48	0.44	0.14	0.13	0.22	0.17	0.47	0.34	0.37	0.15	0.27	0.10	0.24	0.37	1

Note. Pearson correlation coefficients are presented in the lower hemimatrix and p-values in the upper hemimatrix.

Supplementary Table 21. Inter-item and Item-total correlation matrices of SHDI

Food components	1	2	3	4	5	6	7	8	9	10	11	12	13	14	15	16
1. All vegetables	1	0.0000	0.0978	0.0530	0.0017	0.9249	0.0000	0.0551	0.7092	0.0011	0.2146	0.0121	0.8449	0.0001	0.0166	0.0000
2. All fruits	0.34	1	0.1818	0.8007	0.0084	0.0001	0.1307	0.0112	0.0012	0.0060	0.0107	0.0000	0.9428	0.0291	0.3884	0.0000
3. Unsaturated oils	0.04	0.03	1	0.7597	0.0124	0.2817	0.0000	0.1199	0.3487	0.3850	0.0517	0.0023	0.5422	0.4557	0.0000	0.0000
4. Beans, lentils and peas	0.05	-0.01	0.01	1	0.7742	0.0010	0.5125	0.1231	0.0004	0.8490	0.2177	0.1697	0.0650	0.5248	0.6362	0.0000
5. Peanuts and tree nuts	0.08	0.06	0.06	0.01	1	0.0004	0.6925	0.0000	0.0573	0.0206	0.2864	0.2080	0.7663	0.6794	0.3666	0.0000
6. Whole grains	0.00	0.10	0.03	0.08	0.08	1	0.9927	0.0064	0.3101	0.0000	0.3640	0.9102	0.7246	0.8663	0.2613	0.0000
7. Potatoes and cassava	-0.11	-0.04	0.12	0.02	0.01	0.00	1	0.8946	0.0047	0.6490	0.1211	0.0032	0.3460	0.0487	0.3018	0.0000
8. Fish	0.05	0.06	-0.04	0.04	0.10	0.07	0.00	1	0.2394	0.2152	0.0783	0.0001	0.8669	0.0009	0.5533	0.0000
9. Added sugar	0.01	0.08	0.02	-0.09	-0.05	0.02	-0.07	0.03	1	0.0000	0.7978	0.2191	0.7214	0.0003	0.0007	0.0000
10. Refined grains	0.08	0.07	-0.02	0.00	0.06	0.12	0.01	0.03	0.22	1	0.3369	0.2403	0.1764	0.0208	0.8480	0.0000
11. Beef and lamb	-0.03	-0.06	-0.05	-0.03	0.03	0.02	-0.04	0.04	-0.01	0.02	1	0.7975	0.0943	0.8748	0.1304	0.0000
12. Pork	0.06	0.11	-0.07	-0.03	0.03	0.00	-0.07	0.09	-0.03	0.03	0.01	1	0.6773	0.8087	0.8393	0.0000
13. Poultry	0.00	0.00	-0.01	0.04	-0.01	0.01	-0.02	0.00	0.01	-0.03	0.04	0.01	1	0.0734	0.0576	0.0000
14. Dairy	0.09	0.05	-0.02	0.02	-0.01	0.00	-0.05	0.08	-0.09	-0.06	0.00	0.01	0.04	1	0.0956	0.0000
15. Eggs	-0.06	-0.02	-0.11	0.01	-0.02	0.03	-0.02	0.01	-0.08	0.00	0.04	0.00	0.05	0.04	1	0.0000
16. SHDI total score	0.40	0.46	0.17	0.20	0.21	0.27	0.23	0.43	0.22	0.32	0.13	0.25	0.32	0.29	0.26	1

Note. Pearson correlation coefficients are presented in the lower hemimatrix and p-values in the upper hemimatrix.

**Supplementary Table 22. Inter-item and Item-total correlation matrices of HSDI**														
**Food components**	**1**	**2**	**3**	**4**	**5**	**6**	**7**	**8**	**9**	**10**	**11**	**12**	**13**	**14**
1. Whole grain foods	1	0.0076	0.1589	0.3641	0.0092	0.0653	0.3110	0.3046	1.0000	1.0000	0.4796	1.0000	0.7189	0.0001
2. Tubers or starchy vegetables	0.45	1	0.0000	0.0145	0.0240	0.0044	0.6916	0.0075	0.9604	0.5222	0.6180	0.1018	0.6438	0.0001
3. Vegetables	0.29	0.21	1	0.0212	0.0000	0.3826	0.3210	0.9211	0.0010	0.5229	0.0000	0.0234	0.0003	0.0001
4. Milk and dairy products	0.12	-0.12	-0.11	1	0.8399	0.4643	0.2575	0.8421	0.0958	0.0245	0.6387	0.1746	0.2146	0.0001
5. Fruits	0.44	0.09	0.44	0.01	1	0.5152	0.8824	0.0547	0.0000	0.5175	0.0000	0.0814	0.0004	0.0001
6. Beef and pork	0.33	0.18	0.06	0.05	0.04	1	0.3364	0.0868	0.0411	0.1051	0.4507	0.0101	1.0000	0.0001
7. Chicken and other poultry	0.18	-0.02	-0.04	-0.05	0.01	-0.06	1	0.0003	0.4949	1.0000	0.0445	0.8052	0.0202	0.0001
8. Eggs	-0.19	-0.10	0.00	-0.01	0.07	-0.11	-0.14	1	0.0576	0.5040	0.0000	0.7121	0.0002	0.0001
9. Fish and seafood	0.06	0.00	-0.13	0.08	-0.20	-0.13	-0.03	-0.07	1	1.0000	0.0859	0.6211	0.0229	0.0001
10. Legumes, soy and tree nuts	0.62	-0.12	0.12	0.48	-0.13	0.48	-0.04	-0.17	-0.04	1	0.1784	1.0000	1.0000	0.0770
11. Saturated fats	-0.16	0.02	0.16	0.02	0.17	0.05	-0.08	0.24	-0.07	0.26	1	0.0000	0.0000	0.0001
12. Unsaturated oils	0.08	0.12	0.17	0.11	0.12	0.24	-0.02	0.03	-0.04	0.33	0.37	1	0.0067	0.0001
13. Added sugars	-0.11	-0.02	0.15	0.06	0.14	-0.01	-0.09	0.14	-0.09	0.11	0.24	0.19	1	0.0001
14. HSDI total score*	0.10	0.35	0.46	0.22	0.46	0.16	0.23	0.33	0.20	0.04	0.47	0.24	0.41	1

Note. Tetrachoric correlation and point-biserial correlation (*) coefficients are presented in the lower hemimatrix and p-values in the upper hemimatrix.

Supplementary Table 23. Inter-item and Item-total correlation matrices of ELDS

Food components	1	2	3	4	5	6	7	8	9	10	11	12	13	14	15
1. Whole grains	1	0.0394	0.0413	0.0000	0.0284	0.0139	0.7927	0.2983	0.0566	0.4556	0.0003	0.0289	0.9585	0.2009	0.0001
2. Tubers and starchy vegetables	0.09	1	0.0004	0.8518	0.6249	0.0173	0.8773	0.5824	0.0937	0.5565	1.0000	0.5475	0.1790	0.8514	0.0001
3. Vegetables	0.08	0.15	1	0.0000	0.4233	0.1887	0.7528	0.6180	0.8558	0.8126	0.0144	0.1766	0.1483	0.3990	0.0001
4. Fruits	0.17	-0.01	0.36	1	0.4670	0.1127	0.2384	0.9173	0.0042	0.4577	0.0652	0.2003	0.0059	0.5228	0.0001
5. Dairy foods	-0.13	-0.04	-0.05	-0.05	1	0.4781	0.5196	0.9189	0.0902	1.0000	0.2583	0.7989	0.4139	0.2116	0.0001
6. Beef, lamb, pork	0.12	0.14	0.06	0.08	-0.05	1	0.0000	0.3230	0.0041	0.4973	0.2536	0.0045	0.6724	0.0088	0.0001
7. Chicken, other poultry	-0.01	-0.01	0.02	0.05	0.04	-0.24	1	0.5629	0.0767	0.3497	0.5989	1.0000	0.8471	0.0040	0.0001
8. Eggs	-0.04	0.03	-0.02	-0.01	0.01	-0.05	-0.03	1	0.4031	0.1483	0.8902	0.4531	0.0037	0.5309	0.0001
9. Fish	-0.11	0.10	-0.01	-0.17	0.13	-0.19	-0.13	-0.05	1	0.6431	1.0000	1.0000	0.7099	0.6692	0.0001
10. Dry beans, lentils, peas	-0.10	0.09	-0.05	0.09	-0.02	0.19	0.12	0.16	0.08	1	0.4559	0.5278	0.8091	0.2507	0.0001
11. Soy foods	-0.26	0.01	-0.19	-0.16	-0.23	-0.09	0.05	-0.02	-0.02	0.09	1	0.2887	0.2703	1.0000	0.1409
12. Peanuts or tree nuts	0.15	0.06	0.10	0.10	-0.03	0.22	0.02	-0.05	0.02	-0.06	-0.13	1	0.6174	0.0787	0.0001
13. Added sugars	0.00	0.06	0.06	0.11	0.06	0.02	0.01	0.11	-0.02	0.05	-0.08	0.04	1	0.4819	0.0001
14. Added fats	-0.07	-0.01	-0.04	-0.03	-0.09	-0.18	0.18	0.04	0.04	0.24	0.02	-0.22	-0.04	1	0.0001
15. ELDS total score	0.36	0.35	0.46	0.45	0.13	0.24	0.25	0.33	0.12	0.11	0.04	0.18	0.40	0.19	1

Note. Tetrachoric correlation and point-biserial correlation (*) coefficients are presented in the lower hemimatrix and p-values in the upper hemimatrix.

Supplementary Table 24. Inter-item and Item-total correlation matrices of DI

Food components	1	2	3	4	5	6	7	8	9	10	11	12	13	14	15	16	17	18
1. Vegetables	1	0.1173	0.0938	1.0000	1.0000	0.6502	0.6216	0.3576	0.2020	0.8514	0.7029	0.5268	0.5903	0.1779	0.5734	0.5181	0.9036	0.0001
2. Fruits	0.06	1	0.9127	0.3772	0.3422	0.7737	0.5407	0.5890	0.1505	1.0000	0.5210	0.3919	0.5119	0.6193	0.3605	0.0910	0.4609	0.0001
3. Tubers or starchy vegetables	0.07	0.01	1	0.0729	0.0009	0.6568	0.7124	0.0391	0.3406	1.0000	0.8867	0.0590	0.1026	0.0332	0.8991	0.5337	0.0003	0.0001
4. Dairy foods	0.00	-0.04	-0.10	1	0.0119	0.1049	0.0693	0.5600	0.5220	1.0000	0.0191	0.6343	0.0281	0.7121	0.3081	0.0019	0.0272	0.0001
5. Beef and lamb	0.00	-0.04	0.14	-0.13	1	0.7597	0.0009	0.0214	0.0004	0.6809	0.5830	0.0221	0.0811	0.6916	0.4635	0.5503	0.0879	0.0001
6. Pork	-0.02	0.01	-0.02	-0.08	-0.02	1	0.0024	0.1719	0.0027	0.5072	0.2932	0.0126	0.4863	0.0077	1.0000	0.3839	0.0228	0.0001
7. Chicken and other poultry	0.02	0.03	-0.02	0.09	-0.14	-0.14	1	0.0020	0.0763	0.8285	0.2457	0.4716	0.0142	0.7279	0.2478	0.1416	0.4929	0.0001
8. Eggs	-0.04	-0.02	-0.08	-0.03	-0.09	-0.06	-0.13	1	0.0228	0.2511	1.0000	0.7506	0.0201	0.4992	0.9096	0.0412	0.2252	0.0001
9. Fish	-0.06	0.07	0.05	0.04	-0.17	-0.15	-0.10	-0.11	1	1.0000	0.1912	0.0293	0.0043	0.0868	0.2432	0.0197	0.5921	0.0001
10. Dry beans, lentils, peas	-0.02	-0.01	-0.03	-0.04	-0.05	-0.06	0.03	0.12	-0.05	1	0.2792	1.0000	1.0000	0.3361	0.6408	0.7171	0.6270	0.0002
11. Soy foods	0.03	-0.05	0.01	-0.29	-0.04	-0.08	0.09	0.00	0.10	0.13	1	0.0832	0.1533	0.0219	1.0000	0.1342	0.5167	0.0013
12. All nuts	0.04	-0.06	0.13	0.05	0.15	0.16	-0.05	0.03	-0.16	0.08	-0.18	1	0.1938	0.0029	0.6213	0.6832	0.0302	0.0001
13. All sweeteners	-0.02	-0.03	-0.07	-0.11	-0.07	-0.03	-0.11	0.09	-0.14	-0.01	-0.11	0.09	1	0.001	0.1005	0.0000	0.0143	0.0001
14. Unsaturated oils	0.06	0.02	-0.10	0.03	0.02	0.13	-0.02	-0.03	-0.09	0.14	-0.18	0.20	0.15	1	0.3176	0.0000	0.3544	0.0001
15. Lard or tallow	-0.04	-0.06	0.01	-0.10	0.06	0.01	0.08	0.01	-0.12	-1.00	0.00	0.09	0.12	0.08	1	1.0000	0.7723	0.0001
16. Butter	-0.04	-0.10	0.04	-0.21	-0.04	0.05	-0.09	0.12	-0.16	0.13	-0.14	0.04	0.25	0.34	0.00	1	0.0344	0.0001
17. Whole grains and all grains	-0.01	0.06	-0.30	-0.25	-0.12	-0.16	0.05	-0.09	0.04	-1.00	-0.18	1.00	-0.17	0.09	-0.11	-0.18	1	0.0352
18. DI total score	0.36	0.34	0.29	0.14	0.25	0.24	0.22	0.27	0.11	0.09	0.08	0.23	0.28	0.35	0.16	0.21	0.05	1

Note. Tetrachoric correlation and point-biserial correlation (*) coefficients are presented in the lower hemimatrix and p-values in the upper hemimatrix.

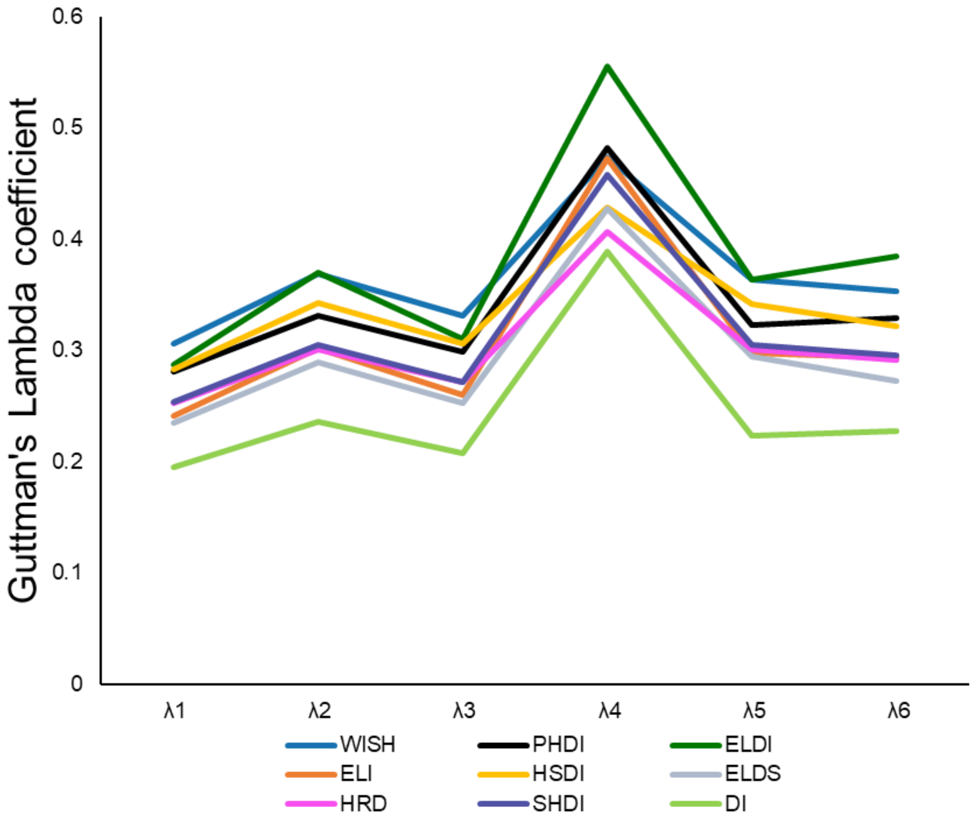

Supplementary Figure 3. Internal consistency reliability of EAT-Lancet indices.

Supplementary Table 25. Correlation between indices and energy intake

	WISH	PHDI	ELDI	HRD	ELI	SHDI	HSDI	ELDS	DI	EI
**WISH**	1	< 0.0001	< 0.0001	< 0.0001	< 0.0001	< 0.0001	< 0.0001	< 0.0001	< 0.0001	< 0.0001
**PHDI**	0.5282	1	< 0.0001	< 0.0001	< 0.0001	< 0.0001	< 0.0001	< 0.0001	< 0.0001	0.0003
**ELDI**	0.6247	0.4492	1	< 0.0001	< 0.0001	< 0.0001	< 0.0001	< 0.0001	< 0.0001	< 0.0001
**HRD**	0.5985	0.4031	0.4736	1	< 0.0001	< 0.0001	< 0.0001	< 0.0001	< 0.0001	0.0002
**ELI**	0.7513	0.5238	0.6295		1	< 0.0001	< 0.0001	< 0.0001	< 0.0001	< 0.0001
**SHDI**	0.5922	0.3449	0.3526	0.703	0.5862	1	0.1741	< 0.0001	< 0.0001	0.4835
**HSDI**	0.5681	0.5173	0.5407	0.1501	0.4195	0.0328	1	< 0.0001	< 0.0001	< 0.0001
**ELDS**	0.6845	0.4553	0.5829	0.5216	0.6370	0.4556	0.4948	1	< 0.0001	< 0.0001
**DI**	0.4828	0.4781	0.4186	0.3454	0.5174	0.2303	0.4417	0.5230	1	0.0007
**Energy intake (kcal/d)**	-0.2542	-0.0881	-0.1066	-0.1056	-0.2789	-0.0169	-0.2274	-0.3055	-0.0813	1

Note. Pearson correlation coefficients are presented in the lower hemimatrix and p-values in the upper hemimatrix.

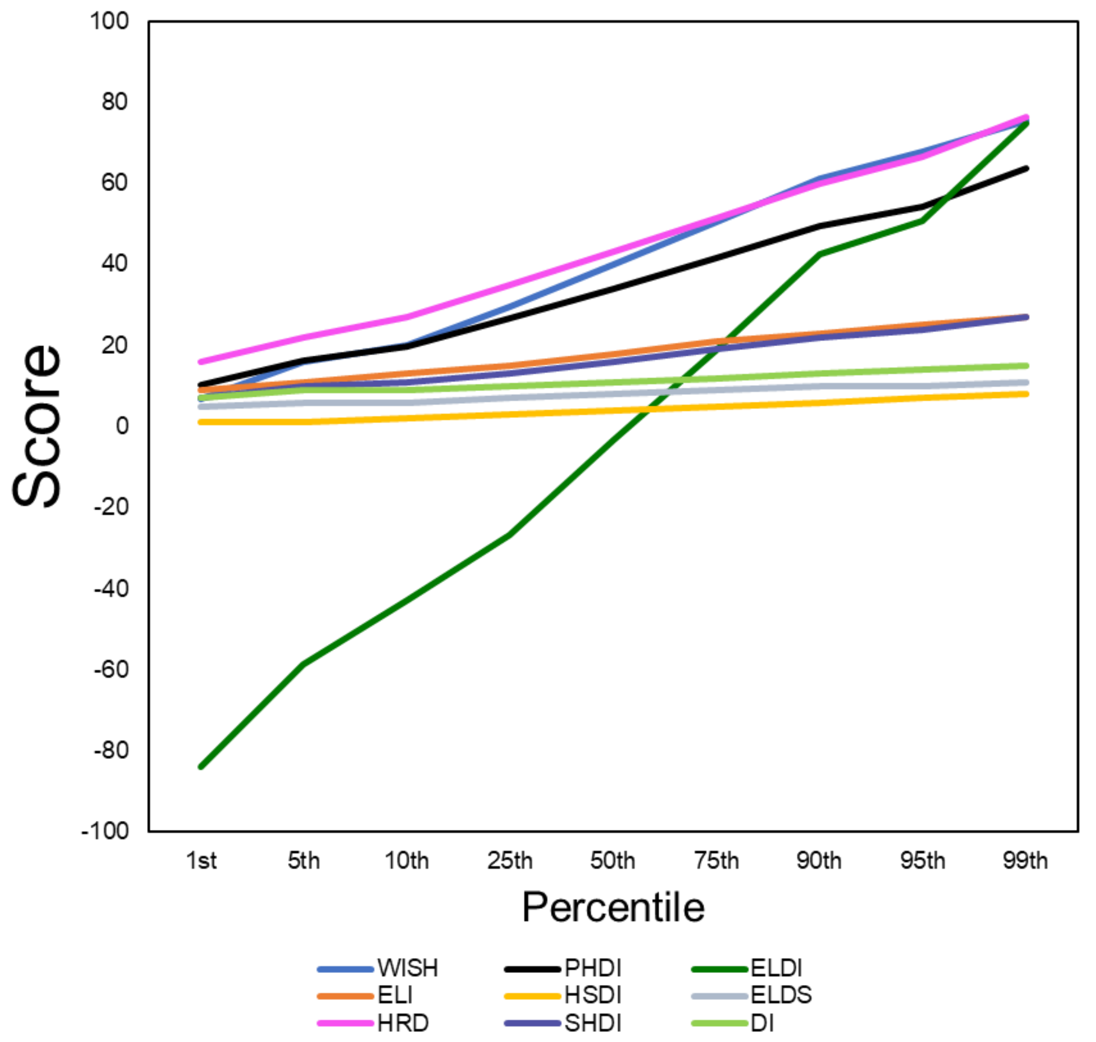

Supplementary Figure 4. Percentile distribution of the of EAT-Lancet indices.

Supplementary Table 26. PANDiet score across quintiles of WISH								
	Q1	Q2	Q3	Q4	Q5	p	η2 (95%CI)	Trend
**PANDiet score**	62.32 (0.27)	63.53 (0.26)	65.53 (0.30)	65.89 (0.27)	67.73 (0.28)	< 0.0001	0.121 (0.092 ─ 0.148)	< 00001
**AS**	61.39 (0.64)	61.94 (0.63)	65.00 (0.70)	64.23 (0.62)	65.04 (0.64)	< 0.0001	0.017 (0.006 ─ 0.029)	< 00001
**Protein**	0.91 (0.01)	0.90 (0.01)	0.89 (0.01)	0.86 (0.01)	0.87 (0.01)	0.0012	0.010 (0.002 ─ 0.020)	0.3343
**LA**	0.45 (0.01)	0.40 (0.02)	0.44 (0.02)	0.44 (0.02)	0.44 (0.02)	0.2165		
**ALA**	0.13 (0.01)	0.10 (0.01)	0.08 (0.01)	0.12 (0.01)	0.14 (0.01)	0.0001	0.014 (0.003 ─ 0.024)	0.0007
**DHA**	0.12 (0.02)	0.20 (0.02)	0.25 (0.02)	0.25 (0.02)	0.33 (0.02)	< 0.0001	0.048 (0.028 ─ 0.067)	< 00001
**EPA+DHA**	0.10 (0.01)	0.17 (0.01)	0.22 (0.02)	0.22 (0.02)	0.29 (0.02)	< 0.0001	0.043 (0.025 ─ 0.062)	< 00001
**Fiber**	0.27 (0.01)	0.33 (0.02)	0.39 (0.02)	0.38 (0.02)	0.43 (0.02)	< 0.0001	0.032 (0.016 ─ 0.048)	< 00001
**Vitamin A**	0.42 (0.02)	0.48 (0.02)	0.50 (0.02)	0.51 (0.02)	0.48 (0.02)	0.0016	0.010 (0.002 ─ 0.019)	0.0380
**Thiamin**	0.92 (0.01)	0.93 (0.01)	0.94 (0.01)	0.95 (0.00)	0.96 (0.00)	< 0.0001	0.024 (0.011 ─ 0.039)	< 00001
**Riboflavin**	0.73 (0.01)	0.75 (0.01)	0.80 (0.01)	0.76 (0.01)	0.74 (0.02)	0.016	0.007 (0.000 ─ 0.015)	0.1720
**Niacin**	1.00 (0.00)	1.00 (0.00)	1.00 (0.00)	1.00 (0.00)	1.00 (0.00)	0.4173		
**Pantothenic acid**	0.88 (0.01)	0.87 (0.01)	0.88 (0.01)	0.88 (0.01)	0.87 (0.01)	0.5537		
**Vitamin B-6**	0.64 (0.02)	0.66 (0.02)	0.73 (0.02)	0.71 (0.02)	0.71 (0.02)	< 0.0001	0.015 (0.004 ─ 0.026)	0.0002
**Folate**	0.61 (0.02)	0.61 (0.02)	0.69 (0.02)	0.70 (0.02)	0.75 (0.01)	< 0.0001	0.029 (0.014 ─ 0.045)	< 00001
**Vitamin B-12**	0.61 (0.02)	0.66 (0.02)	0.72 (0.02)	0.64 (0.02)	0.66 (0.02)	0.0001	0.013 (0.003 ─ 0.024)	0.1378
**Vitamin C**	0.31 (0.02)	0.37 (0.02)	0.45 (0.02)	0.50 (0.02)	0.57 (0.02)	< 0.0001	0.061 (0.040 ─ 0.082)	< 00001
**Vitamin D**	0.02 (0.00)	0.03 (0.00)	0.03 (0.00)	0.04 (0.01)	0.05 (0.01)	0.0004	0.012 (0.003 ─ 0.022)	< 00001
**Vitamin E**	0.85 (0.01)	0.81 (0.01)	0.85 (0.01)	0.83 (0.01)	0.86 (0.01)	0.0054	0.009 (0.001 ─ 0.017)	< 00001
**Iodine**	0.67 (0.02)	0.68 (0.02)	0.74 (0.02)	0.71 (0.01)	0.76 (0.01)	< 0.0001	0.015 (0.004 ─ 0.026)	< 00001
**Magnesium**	0.86 (0.01)	0.87 (0.01)	0.89 (0.01)	0.88 (0.01)	0.88 (0.01)	0.0047	0.009 (0.001 ─ 0.017)	0.0011
**Phosphorus**	1.00 (0.00)	1.00 (0.00)	1.00 (0.00)	1.00 (0.00)	1.00 (0.00)	0.5079		
**Potassium**	0.61 (0.02)	0.61 (0.02)	0.69 (0.02)	0.68 (0.02)	0.68 (0.02)	< 0.0001	0.015 (0.004 ─ 0.026)	0.7435
**Selenium**	0.95 (0.01)	0.92 (0.01)	0.94 (0.01)	0.95 (0.00)	0.95 (0.01)	0.014	0.007 (0.000 ─ 0.015)	< 00001
**Zinc**	0.45 (0.02)	0.41 (0.02)	0.45 (0.02)	0.36 (0.02)	0.29 (0.02)	< 0.0001	0.035 (0.018 ─ 0.052)	0.0103
**Copper**	0.87 (0.01)	0.86 (0.01)	0.88 (0.01)	0.88 (0.01)	0.87 (0.01)	0.3206		
**Manganese**	0.85 (0.01)	0.84 (0.01)	0.85 (0.01)	0.86 (0.01)	0.85 (0.01)	0.4562		
**Calcium**	0.63 (0.02)	0.62 (0.02)	0.67 (0.02)	0.64 (0.02)	0.68 (0.02)	0.0493	0.006 (0.000 ─ 0.012)	0.0666
**Iron**	0.82 (0.01)	0.83 (0.01)	0.82 (0.01)	0.82 (0.01)	0.78 (0.01)	0.0454	0.006 (0.000 ─ 0.013)	0.5863
**MS**	63.24 (0.50)	65.11 (0.50)	66.06 (0.57)	67.55 (0.53)	70.41 (0.52)	< 0.0001	0.056 (0.036 ─ 0.077)	< 00001
**Protein**	0.94 (0.01)	0.93 (0.01)	0.95 (0.01)	0.96 (0.01)	0.97 (0.00)	0.0002	0.013 (0.003 ─ 0.023)	0.5299
**Carbohydrates**	0.88 (0.01)	0.86 (0.01)	0.89 (0.01)	0.88 (0.01)	0.88 (0.01)	0.1959		
**Total fat**	0.84 (0.01)	0.87 (0.01)	0.86 (0.01)	0.85 (0.01)	0.85 (0.01)	0.4772		
**SFA**	0.29 (0.01)	0.30 (0.01)	0.31 (0.01)	0.34 (0.02)	0.41 (0.02)	< 0.0001	0.025 (0.011 ─ 0.039)	< 00001
**Sugars**	0.43 (0.02)	0.51 (0.02)	0.56 (0.02)	0.58 (0.02)	0.66 (0.02)	< 0.0001	0.050 (0.031 ─ 0.070)	< 00001
**Sodium**	0.42 (0.01)	0.43 (0.01)	0.40 (0.01)	0.43 (0.01)	0.45 (0.01)	0.1861		

Note. Data presented as mean (standard error). Q = quintile.

Supplementary Table 27. PANDiet score across quintiles of PHDI								
	**Q1**	**Q2**	**Q3**	**Q4**	**Q5**	**p**	**η2 (95%CI)**	**Trend**
**PANDiet score**	62.59 (0.28)	65.11 (0.26)	65.24 (0.28)	65.33 (0.31)	66.54 (0.29)	< 00001	0.059 (0.038 ─ 0.081)	< 00001
**AS**	60.52 (0.72)	63.74 (0.57)	63.56 (0.63)	64.08 (0.65)	66.18 (0.65)	< 00001	0.022 (0.010 ─ 0.037)	< 00001
**Protein**	0.90 (0.01)	0.90 (0.01)	0.88 (0.01)	0.90 (0.01)	0.87 (0.01)	0.0915		
**LA**	0.39 (0.01)	0.41 (0.02)	0.42 (0.02)	0.45 (0.02)	0.52 (0.02)	< 00001	0.021 (0.008 ─ 0.034)	< 00001
**ALA**	0.11 (0.01)	0.12 (0.01)	0.10 (0.01)	0.13 (0.01)	0.12 (0.01)	0.4103		
**DHA**	0.20 (0.02)	0.25 (0.02)	0.23 (0.02)	0.22 (0.02)	0.20 (0.02)	0.1484		
**EPA+DHA**	0.18 (0.02)	0.22 (0.02)	0.20 (0.02)	0.20 (0.02)	0.18 (0.02)	0.3398		
**Fiber**	0.27 (0.01)	0.31 (0.01)	0.36 (0.02)	0.39 (0.02)	0.48 (0.02)	< 00001	0.053 (0.032 ─ 0.073)	< 00001
**Vitamin A**	0.40 (0.02)	0.50 (0.02)	0.49 (0.02)	0.51 (0.02)	0.49 (0.02)	< 00001	0.015 (0.005 ─ 0.027)	0.0027
**Thiamin**	0.92 (0.01)	0.93 (0.01)	0.95 (0.00)	0.95 (0.00)	0.95 (0.00)	< 00001	0.022 (0.009 ─ 0.036)	0.0005
**Riboflavin**	0.74 (0.02)	0.79 (0.01)	0.75 (0.01)	0.74 (0.02)	0.77 (0.02)	0.0765		
**Niacin**	1.00 (0.00)	1.00 (0.00)	1.00 (0.00)	1.00 (0.00)	1.00 (0.00)	0.5041		
**Pantothenic acid**	0.87 (0.01)	0.88 (0.01)	0.87 (0.01)	0.89 (0.01)	0.88 (0.01)	0.4201		
**Vitamin B-6**	0.63 (0.02)	0.71 (0.01)	0.70 (0.02)	0.68 (0.02)	0.74 (0.02)	< 00001	0.016 (0.005 ─ 0.028)	< 00001
**Folate**	0.57 (0.02)	0.67 (0.02)	0.67 (0.02)	0.71 (0.02)	0.74 (0.02)	< 00001	0.036 (0.019 ─ 0.053)	< 00001
**Vitamin B-12**	0.66 (0.02)	0.69 (0.02)	0.64 (0.02)	0.62 (0.02)	0.68 (0.02)	0.0409	0.006 (0.000 ─ 0.013)	0.6497
**Vitamin C**	0.30 (0.02)	0.41 (0.02)	0.44 (0.02)	0.49 (0.02)	0.56 (0.02)	< 00001	0.051 (0.031 ─ 0.071)	< 00001
**Vitamin D**	0.03 (0.00)	0.03 (0.00)	0.04 (0.01)	0.04 (0.01)	0.03 (0.00)	0.1868		
**Vitamin E**	0.79 (0.01)	0.82 (0.01)	0.85 (0.01)	0.87 (0.01)	0.89 (0.01)	< 00001	0.033 (0.017 ─ 0.050)	< 00001
**Iodine**	0.70 (0.02)	0.71 (0.02)	0.71 (0.01)	0.73 (0.01)	0.70 (0.02)	0.6559		
**Magnesium**	0.84 (0.01)	0.89 (0.01)	0.88 (0.01)	0.88 (0.01)	0.90 (0.01)	< 00001	0.024 (0.010 ─ 0.038)	< 00001
**Phosphorus**	1.00 (0.00)	1.00 (0.00)	1.00 (0.00)	1.00 (0.00)	1.00 (0.00)	0.6008		
**Potassium**	0.58 (0.02)	0.68 (0.01)	0.66 (0.02)	0.63 (0.02)	0.72 (0.02)	< 00001	0.023 (0.010 ─ 0.037)	< 00001
**Selenium**	0.93 (0.01)	0.93 (0.01)	0.94 (0.01)	0.96 (0.00)	0.96 (0.01)	0.0006	0.011 (0.002 ─ 0.021)	0.0456
**Zinc**	0.43 (0.02)	0.43 (0.02)	0.37 (0.02)	0.35 (0.02)	0.40 (0.02)	0.0015	0.010 (0.002 ─ 0.020)	< 00001
**Copper**	0.85 (0.01)	0.86 (0.01)	0.88 (0.01)	0.88 (0.01)	0.91 (0.01)	< 00001	0.021 (0.008 ─ 0.034)	< 00001
**Manganese**	0.80 (0.01)	0.83 (0.01)	0.87 (0.01)	0.87 (0.01)	0.89 (0.01)	< 00001	0.033 (0.017 ─ 0.049)	< 00001
**Calcium**	0.64 (0.02)	0.65 (0.02)	0.63 (0.02)	0.65 (0.02)	0.66 (0.02)	0.7134		
**Iron**	0.80 (0.01)	0.81 (0.01)	0.82 (0.01)	0.80 (0.01)	0.85 (0.01)	0.1066		
**MS**	64.67 (0.57)	66.48 (0.54)	66.92 (0.50)	66.58 (0.53)	66.89 (0.56)	0.0104	0.008 (0.001 ─ 0.016)	0.0366
**Protein**	0.92 (0.01)	0.95 (0.01)	0.96 (0.01)	0.97 (0.00)	0.96 (0.01)	< 00001	0.023 (0.010 ─ 0.038)	0.6091
**Carbohydrates**	0.87 (0.01)	0.88 (0.01)	0.87 (0.01)	0.88 (0.01)	0.90 (0.01)	0.1697		
**Total fat**	0.87 (0.01)	0.86 (0.01)	0.87 (0.01)	0.84 (0.01)	0.83 (0.01)	0.0051	0.009 (0.001 ─ 0.017)	0.0096
**SFA**	0.30 (0.01)	0.32 (0.01)	0.34 (0.01)	0.33 (0.01)	0.34 (0.02)	0.2523		
**Sugars**	0.48 (0.02)	0.55 (0.02)	0.55 (0.02)	0.57 (0.02)	0.57 (0.02)	0.0041	0.009 (0.001 ─ 0.018)	0.0318
**Sodium**	0.44 (0.01)	0.44 (0.01)	0.42 (0.01)	0.41 (0.01)	0.42 (0.02)	0.4908		

Note. Data presented as mean (standard error). Q = quintile.

Supplementary Table 28. PANDiet score across quintiles of ELD-I								
	Q1	Q2	Q3	Q4	Q5	P	η2 (95%CI)	Trend
**PANDiet score**	62.97 (0.31)	64.28 (0.28)	65.26 (0.28)	65.97 (0.25)	66.36 (0.29)	< 00001	0.053 (0.033 ─ 0.073)	< 00001
**AS**	60.24 (0.72)	63.31 (0.64)	64.64 (0.56)	65.69 (0.56)	64.20 (0.70)	< 00001	0.026 (0.011 ─ 0.040)	< 00001
**Protein**	0.88 (0.01)	0.91 (0.01)	0.92 (0.01)	0.89 (0.01)	0.84 (0.01)	< 00001	0.017 (0.006 ─ 0.029)	0.3432
**LA**	0.44 (0.01)	0.50 (0.02)	0.42 (0.02)	0.39 (0.02)	0.40 (0.02)	< 00001	0.017 (0.006 ─ 0.030)	0.1223
**ALA**	0.11 (0.01)	0.14 (0.01)	0.10 (0.01)	0.11 (0.01)	0.11 (0.01)	0.1301		
**DHA**	0.22 (0.02)	0.17 (0.02)	0.21 (0.02)	0.27 (0.02)	0.25 (0.02)	0.0011	0.011 (0.002 ─ 0.020)	0.0001
**EPA+DHA**	0.19 (0.02)	0.15 (0.01)	0.19 (0.02)	0.23 (0.02)	0.22 (0.02)	0.0030	0.009 (0.001 ─ 0.018)	< 00001
**Fiber**	0.24 (0.01)	0.30 (0.02)	0.37 (0.02)	0.42 (0.02)	0.49 (0.02)	< 00001	0.079 (0.055 ─ 0.103)	< 00001
**Vitamin A**	0.42 (0.02)	0.44 (0.02)	0.49 (0.02)	0.52 (0.02)	0.54 (0.02)	< 00001	0.020 (0.008 ─ 0.034)	< 00001
**Thiamin**	0.92 (0.01)	0.94 (0.01)	0.94 (0.00)	0.94 (0.00)	0.95 (0.00)	< 00001	0.019 (0.007 ─ 0.032)	< 00001
**Riboflavin**	0.72 (0.02)	0.75 (0.01)	0.79 (0.01)	0.81 (0.01)	0.72 (0.02)	< 00001	0.018 (0.006 ─ 0.030)	0.2640
**Niacin**	1.00 (0.00)	1.00 (0.00)	1.00 (0.00)	1.00 (0.00)	0.99 (0.00)	0.0220	0.007 (0.000 ─ 0.014)	0.0073
**Pantothenic acid**	0.85 (0.01)	0.88 (0.01)	0.90 (0.01)	0.90 (0.01)	0.86 (0.01)	< 00001	0.018 (0.006 ─ 0.031)	0.0859
**Vitamin B-6**	0.65 (0.02)	0.67 (0.02)	0.71 (0.01)	0.74 (0.01)	0.71 (0.02)	0.0004	0.012 (0.003 ─ 0.022)	< 00001
**Folate**	0.53 (0.02)	0.64 (0.02)	0.72 (0.01)	0.74 (0.01)	0.75 (0.01)	< 00001	0.073 (0.049 ─ 0.095)	< 00001
**Vitamin B-12**	0.69 (0.02)	0.66 (0.02)	0.69 (0.02)	0.67 (0.02)	0.57 (0.02)	< 00001	0.019 (0.007 ─ 0.032)	0.0002
**Vitamin C**	0.25 (0.02)	0.38 (0.02)	0.43 (0.02)	0.52 (0.02)	0.64 (0.02)	< 00001	0.127 (0.098 ─ 0.155)	< 00001
**Vitamin D**	0.03 (0.00)	0.03 (0.00)	0.04 (0.01)	0.04 (0.01)	0.03 (0.00)	0.0223	0.007 (0.000 ─ 0.014)	0.6472
**Vitamin E**	0.80 (0.01)	0.86 (0.01)	0.86 (0.01)	0.85 (0.01)	0.85 (0.01)	< 00001	0.020 (0.007 ─ 0.032)	< 00001
**Iodine**	0.65 (0.02)	0.69 (0.02)	0.73 (0.01)	0.76 (0.01)	0.73 (0.02)	< 00001	0.020 (0.007 ─ 0.033)	< 00001
**Magnesium**	0.85 (0.01)	0.87 (0.01)	0.89 (0.01)	0.89 (0.01)	0.88 (0.01)	0.0001	0.014 (0.004 ─ 0.024)	< 00001
**Phosphorus**	1.00 (0.00)	1.00 (0.00)	1.00 (0.00)	1.00 (0.00)	1.00 (0.00)	0.6737		
**Potassium**	0.59 (0.02)	0.65 (0.02)	0.66 (0.02)	0.70 (0.01)	0.67 (0.02)	< 00001	0.015 (0.004 ─ 0.027)	< 00001
**Selenium**	0.93 (0.01)	0.95 (0.01)	0.94 (0.01)	0.94 (0.01)	0.96 (0.01)	0.0727		
**Zinc**	0.53 (0.02)	0.45 (0.02)	0.38 (0.02)	0.34 (0.02)	0.24 (0.01)	< 00001	0.090 (0.064 ─ 0.114)	< 00001
**Copper**	0.84 (0.01)	0.88 (0.01)	0.89 (0.01)	0.89 (0.01)	0.88 (0.01)	< 00001	0.023 (0.009 ─ 0.036)	< 00001
**Manganese**	0.77 (0.01)	0.85 (0.01)	0.88 (0.01)	0.89 (0.01)	0.87 (0.01)	< 00001	0.064 (0.042 ─ 0.085)	< 00001
**Calcium**	0.57 (0.02)	0.64 (0.02)	0.68 (0.02)	0.69 (0.02)	0.67 (0.02)	< 00001	0.017 (0.006 ─ 0.029)	0.0001
**Iron**	0.80 (0.01)	0.85 (0.01)	0.85 (0.01)	0.83 (0.01)	0.76 (0.01)	< 00001	0.021 (0.008 ─ 0.034)	0.9655
**MS**	65.69 (0.57)	65.25 (0.55)	65.87 (0.55)	66.25 (0.50)	68.52 (0.51)	0.0004	0.012 (0.003 ─ 0.022)	0.0007
**Protein**	0.91 (0.01)	0.95 (0.01)	0.96 (0.01)	0.95 (0.01)	0.98 (0.00)	< 00001	0.038 (0.020 ─ 0.055)	0.0085
**Carbohydrates**	0.89 (0.01)	0.91 (0.01)	0.87 (0.01)	0.88 (0.01)	0.84 (0.01)	< 00001	0.024 (0.010 ─ 0.038)	0.0001
**Total fat**	0.81 (0.01)	0.85 (0.01)	0.87 (0.01)	0.88 (0.01)	0.89 (0.01)	< 00001	0.031 (0.016 ─ 0.047)	< 00001
**SFA**	0.29 (0.01)	0.29 (0.01)	0.29 (0.01)	0.35 (0.01)	0.43 (0.02)	< 00001	0.042 (0.024 ─ 0.060)	< 00001
**Sugars**	0.55 (0.02)	0.51 (0.02)	0.55 (0.02)	0.53 (0.02)	0.56 (0.02)	0.2676		
**Sodium**	0.49 (0.01)	0.41 (0.01)	0.41 (0.01)	0.39 (0.01)	0.42 (0.02)	< 00001	0.018 (0.006 ─ 0.030)	< 00001

Note. Data presented as mean (standard error). Q = quintile.

Supplementary Table 29. PANDiet score across quintiles of HRD

	Q1	Q2	Q3	Q4	Q5	P	η2 (95%CI)	Trend
**PANDiet score**	62.87 (0.29)	63.82 (0.29)	64.64 (0.28)	65.87 (0.27)	67.75 (0.26)	< 00001	0.091 (0.065 – 0.115)	< 00001
**AS**	60.73 (0.68)	62.27 (0.66)	64.20 (0.69)	65.06 (0.62)	65.63 (0.55)	< 00001	0.022 (0.001 – 0.036)	< 00001
**Protein**	0.87 (0.01)	0.89 (0.01)	0.88 (0.01)	0.90 (0.01)	0.91 (0.01)	0.0894		
**LA**	0.39 (0.01)	0.44 (0.02)	0.39 (0.02)	0.49 (0.02)	0.47 (0.02)	< 00001	0.018 (0.007 – 0.031)	< 00001
**ALA**	0.10 (0.01)	0.11 (0.01)	0.09 (0.01)	0.14 (0.01)	0.15 (0.01)	0.0002	0.013 (0.003 – 0.023)	< 00001
**DHA**	0.11 (0.01)	0.18 (0.02)	0.27 (0.02)	0.25 (0.02)	0.33 (0.02)	< 00001	0.059 (0.038 – 0.080)	< 00001
**EPA+DHA**	0.10 (0.01)	0.15 (0.02)	0.23 (0.02)	0.22 (0.02)	0.30 (0.02)	< 00001	0.052 (0.032 – 0.072)	< 00001
**Fiber**	0.29 (0.02)	0.32 (0.02)	0.37 (0.02)	0.38 (0.02)	0.44 (0.02)	< 00001	0.026 (0.012 – 0.041)	< 00001
**Vitamin A**	0.44 (0.02)	0.47 (0.02)	0.52 (0.02)	0.49 (0.02)	0.46 (0.02)	0.0079	0.008 (0.001 – 0.016)	0.586
**Thiamin**	0.92 (0.01)	0.93 (0.01)	0.95 (0.00)	0.94 (0.01)	0.96 (0.00)	< 00001	0.022 (0.009 – 0.036)	< 00001
**Riboflavin**	0.75 (0.01)	0.75 (0.01)	0.77 (0.01)	0.79 (0.01)	0.73 (0.02)	0.0924		
**Niacin**	1.00 (0.00)	1.00 (0.00)	1.00 (0.00)	1.00 (0.00)	1.00 (0.00)	0.8018		
**Pantothenic acid**	0.87 (0.01)	0.87 (0.01)	0.87 (0.01)	0.90 (0.01)	0.89 (0.01)	0.0291	0.006 (0.000 – 0.014)	0.2248
**Vitamin B-6**	0.63 (0.02)	0.66 (0.02)	0.69 (0.02)	0.72 (0.01)	0.77 (0.01)	< 00001	0.024 (0.011 – 0.039)	< 00001
**Folate**	0.60 (0.02)	0.64 (0.02)	0.66 (0.02)	0.72 (0.02)	0.74 (0.01)	< 00001	0.028 (0.013 – 0.043)	< 00001
**Vitamin B-12**	0.63 (0.02)	0.63 (0.02)	0.67 (0.02)	0.70 (0.02)	0.66 (0.02)	0.0141	0.007 (0.000 – 0.015)	0.7405
**Vitamin C**	0.34 (0.02)	0.38 (0.02)	0.50 (0.02)	0.41 (0.02)	0.55 (0.02)	< 00001	0.042 (0.024 – 0.059)	< 00001
**Vitamin D**	0.02 (0.00)	0.04 (0.00)	0.03 (0.00)	0.04 (0.01)	0.04 (0.01)	0.0588		
**Vitamin E**	0.79 (0.01)	0.85 (0.01)	0.85 (0.01)	0.86 (0.01)	0.87 (0.01)	< 00001	0.025 (0.011 – 0.039)	< 00001
**Iodine**	0.68 (0.02)	0.68 (0.02)	0.73 (0.01)	0.74 (0.01)	0.73 (0.01)	0.0008	0.011 (0.002 – 0.021)	0.0558
**Magnesium**	0.85 (0.01)	0.87 (0.01)	0.89 (0.01)	0.88 (0.01)	0.89 (0.01)	0.0006	0.011 (0.002 – 0.021)	< 00001
**Phosphorus**	1.00 (0.00)	1.00 (0.00)	1.00 (0.00)	1.00 (0.00)	1.00 (0.00)	0.9339		
**Potassium**	0.62 (0.02)	0.61 (0.02)	0.67 (0.02)	0.68 (0.02)	0.68 (0.02)	0.0022	0.010 (0.001 – 0.018)	< 00001
**Selenium**	0.93 (0.01)	0.93 (0.01)	0.93 (0.01)	0.95 (0.01)	0.97 (0.00)	0.0004	0.012 (0.003 – 0.022)	< 00001
**Zinc**	0.45 (0.02)	0.44 (0.02)	0.43 (0.02)	0.36 (0.02)	0.28 (0.02)	< 00001	0.032 (0.016 – 0.049)	< 00001
**Copper**	0.86 (0.01)	0.87 (0.01)	0.88 (0.01)	0.88 (0.01)	0.89 (0.01)	0.0243	0.007 (0.000 – 0.14)	< 00001
**Manganese**	0.81 (0.01)	0.85 (0.01)	0.84 (0.01)	0.86 (0.01)	0.88 (0.01)	0.0001	0.013 (0.003 – 0.024)	< 00001
**Calcium**	0.63 (0.02)	0.63 (0.02)	0.67 (0.02)	0.66 (0.02)	0.63 (0.02)	0.3277		
**Iron**	0.82 (0.01)	0.80 (0.01)	0.81 (0.01)	0.83 (0.01)	0.83 (0.01)	0.2751		
**MS**	65.01 (0.53)	65.38 (0.52)	65.07 (0.53)	66.67 (0.55)	69.88 (0.54)	< 00001	0.031 (0.015 – 0.045)	< 00001
**Protein**	0.94 (0.01)	0.95 (0.01)	0.94 (0.01)	0.94 (0.01)	0.96 (0.01)	0.1139		
**Carbohydrates**	0.86 (0.01)	0.87 (0.01)	0.88 (0.01)	0.89 (0.01)	0.89 (0.01)	0.0062	0.008 (0.008 – 0.017)	0.0007
**Total fat**	0.85 (0.01)	0.85 (0.01)	0.85 (0.01)	0.85 (0.01)	0.87 (0.01)	0.7005		
**SFA**	0.31 (0.01)	0.29 (0.01)	0.31 (0.01)	0.33 (0.01)	0.41 (0.02)	< 00001	0.022 (0.009 – 0.036)	< 00001
**Sugars**	0.48 (0.02)	0.55 (0.02)	0.50 (0.02)	0.56 (0.02)	0.64 (0.02)	< 00001	0.023 (0.009 – 0.036)	< 00001
**Sodium**	0.46 (0.01)	0.41 (0.01)	0.42 (0.01)	0.41 (0.01)	0.43 (0.01)	0.0364	0.006 (0.000 – 0.013)	0.402

Note. Data presented as mean (standard error). Q = quintile.

Supplementary Table 30. PANDiet score across quintiles of ELI								
	**Q1**	**Q2**	**Q3**	**Q4**	**Q5**	**P**	**η2 (95%CI)**	**Trend**
**PANDiet score**	62.92 (0.25)	64.05 (0.24)	65.96 (0.29)	65.75 (0.34)	67.51 (0.32)	< 00001	0.083 (0.058 ─ 0.107)	< 00001
**AS**	62.40 (0.59)	62.08 (0.61)	65.87 (0.65)	64.48 (0.70)	63.76 (0.69)	0.0001	0.014 (0.004 ─ 0.025)	0.0020
**Protein**	0.93 (0.01)	0.85 (0.01)	0.91 (0.01)	0.89 (0.01)	0.86 (0.01)	< 00001	0.031 (0.015 ─ 0.046)	0.0002
**LA**	0.43 (0.01)	0.41 (0.01)	0.44 (0.02)	0.40 (0.02)	0.50 (0.02)	0.0026	0.009 (0.001 ─ 0.019)	0.0076
**ALA**	0.12 (0.01)	0.09 (0.01)	0.13 (0.01)	0.10 (0.01)	0.15 (0.01)	0.0009	0.011 (0.002 ─ 0.021)	< 00001
**DHA**	0.07 (0.01)	0.21 (0.01)	0.24 (0.02)	0.30 (0.02)	0.42 (0.02)	< 00001	0.116 (0.088 ─ 0.143)	< 00001
**EPA+DHA**	0.06 (0.01)	0.18 (0.01)	0.22 (0.02)	0.26 (0.02)	0.38 (0.02)	< 00001	0.108 (0.080 ─ 0.134)	< 00001
**Fiber**	0.30 (0.02)	0.31 (0.01)	0.41 (0.02)	0.41 (0.02)	0.42 (0.02)	< 00001	0.031 (0.015 ─ 0.047)	< 00001
**Vitamin A**	0.45 (0.02)	0.47 (0.02)	0.49 (0.02)	0.52 (0.02)	0.49 (0.02)	0.1306		
**Thiamin**	0.93 (0.00)	0.92 (0.01)	0.95 (0.00)	0.93 (0.01)	0.96 (0.00)	< 00001	0.021 (0.008 ─ 0.035)	0.0001
**Riboflavin**	0.76 (0.01)	0.76 (0.01)	0.80 (0.02)	0.74 (0.02)	0.70 (0.02)	0.0003	0.012 (0.003 ─ 0.023)	< 00001
**Niacin**	1.00 (0.00)	1.00 (0.00)	1.00 (0.00)	1.00 (0.00)	1.00 (0.00)	0.3479		
**Pantothenic acid**	0.89 (0.01)	0.87 (0.01)	0.90 (0.01)	0.88 (0.01)	0.85 (0.01)	0.0002	0.012 (0.003 ─ 0.023)	0.0003
**Vitamin B-6**	0.67 (0.02)	0.68 (0.01)	0.74 (0.02)	0.71 (0.02)	0.66 (0.02)	0.0040	0.009 (0.001 ─ 0.018)	0.7934
**Folate**	0.62 (0.02)	0.61 (0.02)	0.72 (0.02)	0.75 (0.02)	0.72 (0.02)	< 00001	0.036 (0.019 ─ 0.052)	< 00001
**Vitamin B-12**	0.65 (0.02)	0.66 (0.01)	0.69 (0.02)	0.64 (0.02)	0.64 (0.02)	0.1816		
**Vitamin C**	0.31 (0.02)	0.39 (0.02)	0.50 (0.02)	0.57 (0.02)	0.52 (0.02)	< 00001	0.062 (0.041 ─ 0.084)	< 00001
**Vitamin D**	0.02 (0.00)	0.03 (0.00)	0.03 (0.00)	0.06 (0.01)	0.04 (0.01)	< 00001	0.023 (0.010 ─ 0.037)	< 00001
**Vitamin E**	0.82 (0.01)	0.82 (0.01)	0.86 (0.01)	0.85 (0.01)	0.89 (0.01)	< 00001	0.022 (0.009 ─ 0.035)	< 00001
**Iodine**	0.68 (0.01)	0.69 (0.01)	0.75 (0.02)	0.74 (0.02)	0.72 (0.02)	0.0063	0.008 (0.001 ─ 0.017)	0.0426
**Magnesium**	0.87 (0.01)	0.87 (0.01)	0.89 (0.01)	0.87 (0.01)	0.88 (0.01)	0.0852		
**Phosphorus**	1.00 (0.00)	1.00 (0.00)	1.00 (0.00)	1.00 (0.00)	1.00 (0.00)	0.6414		
**Potassium**	0.64 (0.02)	0.63 (0.01)	0.70 (0.02)	0.67 (0.02)	0.64 (0.02)	0.0052	0.009 (0.001 ─ 0.017)	0.5545
**Selenium**	0.95 (0.01)	0.93 (0.01)	0.95 (0.01)	0.94 (0.01)	0.94 (0.01)	0.0535		
**Zinc**	0.51 (0.02)	0.44 (0.02)	0.42 (0.02)	0.31 (0.02)	0.17 (0.01)	< 00001	0.111 (0.084 ─ 0.138)	< 00001
**Copper**	0.87 (0.01)	0.87 (0.01)	0.89 (0.01)	0.87 (0.01)	0.87 (0.01)	0.2748		
**Manganese**	0.83 (0.01)	0.84 (0.01)	0.87 (0.01)	0.84 (0.01)	0.87 (0.01)	0.0095	0.008 (0.001 ─ 0.016)	< 00001
**Calcium**	0.67 (0.02)	0.63 (0.02)	0.68 (0.02)	0.66 (0.02)	0.58 (0.02)	0.0026	0.009 (0.001 ─ 0.019)	< 00001
**Iron**	0.85 (0.01)	0.81 (0.01)	0.85 (0.01)	0.78 (0.01)	0.76 (0.01)	< 00001	0.021 (0.008 ─ 0.034)	0.0001
**MS**	63.43 (0.49)	66.02 (0.46)	66.05 (0.52)	67.01 (0.69)	71.25 (0.53)	< 00001	0.056 (0.035 ─ 0.077)	< 00001
**Protein**	0.94 (0.01)	0.94 (0.01)	0.96 (0.01)	0.94 (0.01)	0.98 (0.00)	< 00001	0.017 (0.006 ─ 0.029)	0.0016
**Carbohydrates**	0.87 (0.01)	0.87 (0.01)	0.91 (0.01)	0.85 (0.01)	0.90 (0.01)	< 00001	0.017 (0.006 ─ 0.029)	0.0198
**Total fat**	0.85 (0.01)	0.86 (0.01)	0.85 (0.01)	0.87 (0.01)	0.84 (0.19)	0.6511		
**SFA**	0.28 (0.01)	0.31 (0.01)	0.31 (0.01)	0.40 (0.02)	0.40 (0.02)	< 00001	0.032 (0.016 ─ 0.048)	< 00001
**Sugars**	0.45 (0.02)	0.56 (0.02)	0.54 (0.02)	0.56 (0.02)	0.67 (0.02)	< 00001	0.037 (0.020 ─ 0.054)	< 00001
**Sodium**	0.41 (0.01)	0.43 (0.01)	0.39 (0.02)	0.41 (0.02)	0.49 (0.02)	0.0002	0.013 (0.003 ─ 0.023)	0.0282

Note. Data presented as mean (standard error). Q = quintile.

Supplementary Table 31. PANDiet score across quintiles of SHDI

	Q1	Q2	Q3	Q4	Q5	P	η2 (95%CI)	Trend
**PANDiet score**	62.04 (0.26)	64.69 (0.24)	64.97 (0.28)	66.43 (0.30)	68.24 (0.29)	< 00001	0.141 (0.111 ─ 0.170)	< 00001
**AS**	57.42 (0.64)	63.75 (0.57)	64.78 (0.65)	66.36 (0.65)	68.91 (0.51)	< 00001	0.110 (0.079 ─ 0.133)	< 00001
**Protein**	0.87 (0.01)	0.91 (0.01)	0.87 (0.01)	0.90 (0.01)	0.90 (0.01)	0.0022	0.010 (0.001 ─ 0.019)	0.0343
**LA**	0.39 (0.01)	0.43 (0.01)	0.43 (0.02)	0.48 (0.02)	0.47 (0.02)	0.0005	0.012 (0.002 ─ 0.022)	< 00001
**ALA**	0.10 (0.01)	0.10 (0.01)	0.10 (0.01)	0.15 (0.01)	0.16 (0.01)	< 00001	0.015 (0.004 ─ 0.026)	< 00001
**DHA**	0.08 (0.01)	0.18 (0.01)	0.30 (0.02)	0.27 (0.02)	0.39 (0.02)	< 00001	0.114 (0.086 ─ 0.140)	< 00001
**EPA+DHA**	0.07 (0.01)	0.16 (0.01)	0.26 (0.02)	0.24 (0.02)	0.35 (0.02)	< 00001	0.103 (0.076 ─ 0.129)	< 00001
**Fiber**	0.23 (0.01)	0.35 (0.01)	0.38 (0.02)	0.41 (0.02)	0.50 (0.02)	< 00001	0.078 (0.054 ─ 0.102)	< 00001
**Vitamin A**	0.36 (0.02)	0.48 (0.02)	0.52 (0.02)	0.56 (0.02)	0.53 (0.02)	< 00001	0.051 (0.031 ─ 0.071)	< 00001
**Thiamin**	0.93 (0.00)	0.93 (0.00)	0.93 (0.01)	0.95 (0.00)	0.96 (0.00)	< 00001	0.021 (0.007 ─ 0.033)	< 00001
**Riboflavin**	0.67 (0.02)	0.78 (0.01)	0.77 (0.01)	0.77 (0.02)	0.83 (0.01)	< 00001	0.040 (0.023 ─ 0.058)	< 00001
**Niacin**	0.99 (0.00)	1.00 (0.00)	1.00 (0.00)	1.00 (0.00)	1.00 (0.00)	0.0453	0.006 (0.000 ─ 0.013)	0.9587
**Pantothenic acid**	0.83 (0.01)	0.89 (0.01)	0.88 (0.01)	0.90 (0.01)	0.92 (0.01)	< 00001	0.044 (0.025 ─ 0.062)	< 00001
**Vitamin B-6**	0.56 (0.02)	0.71 (0.01)	0.70 (0.02)	0.76 (0.02)	0.81 (0.01)	< 00001	0.089 (0.064 ─ 0.113)	< 00001
**Folate**	0.53 (0.02)	0.67 (0.01)	0.71 (0.02)	0.74 (0.02)	0.77 (0.01)	< 00001	0.079 (0.055 ─ 0.102)	< 00001
**Vitamin B-12**	0.57 (0.02)	0.66 (0.01)	0.70 (0.02)	0.69 (0.02)	0.73 (0.02)	< 00001	0.036 (0.019 ─ 0.053)	< 00001
**Vitamin C**	0.31 (0.02)	0.41 (0.02)	0.45 (0.02)	0.52 (0.02)	0.57 (0.02)	< 00001	0.061 (0.039 ─ 0.081)	< 00001
**Vitamin D**	0.02 (0.00)	0.03 (0.00)	0.04 (0.01)	0.05 (0.01)	0.05 (0.01)	< 00001	0.023 (0.010 ─ 0.038)	< 00001
**Vitamin E**	0.79 (0.01)	0.84 (0.01)	0.86 (0.01)	0.86 (0.01)	0.89 (0.01)	< 00001	0.038 (0.021 ─ 0.055)	< 00001
**Iodine**	0.61 (0.02)	0.71 (0.01)	0.74 (0.02)	0.76 (0.02)	0.79 (0.01)	< 00001	0.057 (0.036 ─ 0.078)	< 00001
**Magnesium**	0.83 (0.01)	0.89 (0.01)	0.88 (0.01)	0.89 (0.01)	0.91 (0.01)	< 00001	0.039 (0.022 ─ 0.057)	< 00001
**Phosphorus**	1.00 (0.00)	1.00 (0.00)	1.00 (0.00)	1.00 (0.00)	1.00 (0.00)	0.9649		
**Potassium**	0.52 (0.02)	0.66 (0.01)	0.68 (0.02)	0.69 (0.02)	0.77 (0.01)	< 00001	0.075 (0.051 ─ 0.098)	< 00001
**Selenium**	0.93 (0.01)	0.93 (0.01)	0.94 (0.01)	0.95 (0.01)	0.97 (0.01)	0.0024	0.010 (0.001 ─ 0.019)	< 00001
**Zinc**	0.41 (0.02)	0.44 (0.02)	0.40 (0.02)	0.37 (0.02)	0.33 (0.02)	0.0002	0.013 (0.003 ─ 0.024)	< 00001
**Copper**	0.85 (0.01)	0.88 (0.01)	0.88 (0.01)	0.89 (0.01)	0.90 (0.01)	< 00001	0.016 (0.005 ─ 0.028)	< 00001
**Manganese**	0.82 (0.01)	0.85 (0.01)	0.85 (0.01)	0.85 (0.01)	0.88 (0.01)	0.0008	0.011 (0.002 ─ 0.021)	< 00001
**Calcium**	0.54 (0.02)	0.66 (0.02)	0.66 (0.02)	0.69 (0.02)	0.74 (0.02)	< 00001	0.040 (0.023 ─ 0.058)	< 00001
**Iron**	0.78 (0.01)	0.82 (0.01)	0.83 (0.01)	0.83 (0.01)	0.85 (0.01)	0.0004	0.012 (0.003 ─ 0.022)	0.0007
**MS**	66.66 (0.49)	65.64 (0.46)	65.16 (0.59)	66.50 (0.67)	67.56 (0.57)	0.0286	0.006 (0.000 ─ 0.014)	0.2856
**Protein**	0.96 (0.00)	0.95 (0.01)	0.92 (0.01)	0.95 (0.01)	0.95 (0.01)	< 00001	0.020 (0.008 ─ 0.033)	0.0254
**Carbohydrates**	0.84 (0.01)	0.89 (0.01)	0.88 (0.01)	0.89 (0.01)	0.92 (0.01)	< 00001	0.027 (0.013 ─ 0.043)	< 00001
**Total fat**	0.87 (0.01)	0.86 (0.01)	0.83 (0.01)	0.84 (0.01)	0.85 (0.01)	0.0129	0.007 (0.000 ─ 0.015)	0.0172
**SFA**	0.33 (0.01)	0.31 (0.01)	0.32 (0.01)	0.34 (0.02)	0.34 (0.02)	0.3234		
**Sugars**	0.52 (0.02)	0.55 (0.02)	0.54 (0.02)	0.55 (0.02)	0.56 (0.02)	0.4034		
**Sodium**	0.47 (0.01)	0.38 (0.01)	0.42 (0.02)	0.42 (0.02)	0.43 (0.01)	< 00001	0.015 (0.004 ─ 0.026)	0.3728

Note. Data presented as mean (standard error). Q = quintile.

Supplementary Table 32. PANDiet score across quartiles of HSDI

	Q1	Q2	Q3	Q4	P	η2 (95%CI)	Trend
**PANDiet score**	63.82 (0.21)	65.03 (0.25)	65.79 (0.31)	65.95 (0.32)	< 00001	0.026 (0.012 ─ 0.042)	< 00001
**AS**	63.27 (0.47)	63.86 (0.57)	63.51 (0.65)	63.00 (0.75)	0.7841		
**Protein**	0.92 (0.01)	0.89 (0.01)	0.88 (0.01)	0.83 (0.01)	< 00001	0.026 (0.012 ─ 0.042)	< 00001
**LA**	0.45 (0.01)	0.42 (0.01)	0.42 (0.02)	0.43 (0.02)	0.1980		
**ALA**	0.12 (0.01)	0.12 (0.01)	0.10 (0.01)	0.12 (0.01)	0.3095		
**DHA**	0.25 (0.01)	0.25 (0.02)	0.16 (0.02)	0.16 (0.02)	< 00001	0.018 (0.007 ─ 0.031)	< 00001
**EPA+DHA**	0.22 (0.01)	0.22 (0.02)	0.14 (0.02)	0.15 (0.02)	< 00001	0.014 (0.004 ─ 0.026)	< 00001
**Fiber**	0.30 (0.01)	0.37 (0.01)	0.40 (0.02)	0.43 (0.02)	< 00001	0.027 (0.013 ─ 0.042)	< 00001
**Vitamin A**	0.47 (0.01)	0.48 (0.02)	0.48 (0.02)	0.47 (0.02)	0.9027		
**Thiamin**	0.92 (0.00)	0.94 (0.00)	0.95 (0.00)	0.95 (0.00)	0.0001	0.013 (0.004 ─ 0.024)	0.0001
**Riboflavin**	0.77 (0.01)	0.76 (0.01)	0.77 (0.01)	0.72 (0.02)	0.0848		
**Niacin**	1.00 (0.00)	1.00 (0.00)	1.00 (0.00)	0.99 (0.00)	0.0013	0.009 (0.002 ─ 0.019)	0.0001
**Pantothenic acid**	0.89 (0.00)	0.88 (0.01)	0.87 (0.01)	0.85 (0.01)	0.0007	0.010 (0.002 ─ 0.020)	0.0002
**Vitamin B-6**	0.68 (0.01)	0.70 (0.01)	0.71 (0.02)	0.67 (0.02)	0.3808		
**Folate**	0.63 (0.01)	0.69 (0.01)	0.68 (0.02)	0.71 (0.02)	0.0002	0.012 (0.003 ─ 0.022)	0.0049
**Vitamin B-12**	0.68 (0.01)	0.67 (0.01)	0.66 (0.02)	0.58 (0.02)	0.0001	0.013 (0.004 ─ 0.024)	< 00001
**Vitamin C**	0.32 (0.01)	0.48 (0.02)	0.47 (0.02)	0.58 (0.02)	< 00001	0.071 (0.048 ─ 0.094)	< 00001
**Vitamin D**	0.04 (0.00)	0.03 (0.00)	0.04 (0.01)	0.02 (0.00)	0.1821		
**Vitamin E**	0.84 (0.01)	0.84 (0.01)	0.84 (0.01)	0.84 (0.01)	0.9552		
**Iodine**	0.72 (0.01)	0.71 (0.01)	0.71 (0.02)	0.67 (0.02)	0.0334	0.005 (0.000 ─ 0.012)	0.0209
**Magnesium**	0.87 (0.01)	0.87 (0.01)	0.89 (0.01)	0.87 (0.01)	0.4355		
**Phosphorus**	1.00 (0.00)	1.00 (0.00)	1.00 (0.00)	1.00 (0.00)	0.5941		
**Potassium**	0.64 (0.01)	0.65 (0.01)	0.67 (0.02)	0.66 (0.02)	0.4193		
**Selenium**	0.95 (0.00)	0.94 (0.01)	0.94 (0.01)	0.94 (0.01)	0.4628		
**Zinc**	0.41 (0.01)	0.41 (0.02)	0.36 (0.02)	0.38 (0.02)	0.1243		
**Copper**	0.87 (0.01)	0.87 (0.01)	0.88 (0.01)	0.88 (0.01)	0.2273		
**Manganese**	0.85 (0.01)	0.83 (0.01)	0.86 (0.01)	0.87 (0.01)	0.0255	0.005 (0.000 ─ 0.013)	0.0328
**Calcium**	0.67 (0.01)	0.66 (0.02)	0.61 (0.02)	0.60 (0.02)	0.0026	0.008 (0.001 ─ 0.017)	0.0010
**Iron**	0.83 (0.01)	0.81 (0.01)	0.83 (0.01)	0.79 (0.01)	0.0433	0.005 (0.000 ─ 0.012)	0.0295
**MS**	64.38 (0.39)	66.20 (0.51)	68.06 (0.53)	68.90 (0.51)	< 00001	0.031 (0.016 ─ 0.047)	< 00001
**Protein**	0.94 (0.01)	0.94 (0.01)	0.97 (0.00)	0.97 (0.00)	< 00001	0.017 (0.006 ─ 0.029)	0.3136
**Carbohydrates**	0.89 (0.01)	0.87 (0.01)	0.88 (0.01)	0.86 (0.01)	0.0080	0.007 (0.001 ─ 0.015)	0.3111
**Total fat**	0.84 (0.01)	0.87 (0.01)	0.86 (0.01)	0.85 (0.01)	0.0152	0.006 (0.000 ─ 0.014)	0.2586
**SFA**	0.28 (0.01)	0.34 (0.01)	0.36 (0.01)	0.37 (0.02)	< 00001	0.021 (0.009 ─ 0.035)	< 00001
**Sugars**	0.50 (0.01)	0.51 (0.02)	0.59 (0.02)	0.66 (0.02)	< 00001	0.031 (0.016 ─ 0.048)	< 00001
**Sodium**	0.42 (0.01)	0.45 (0.01)	0.42 (0.01)	0.42 (0.02)	0.1895		

Note. Data presented as mean (standard error). Q = quartile.

**Supplementary Table 33. PANDiet score across quartiles of ELDS**							
	**Q1**	**Q2**	**Q3**	**Q4**	**P**	**η2 (95%CI)**	**Trend**
**PANDiet score**	63.88 (0.24)	65.30 (0.26)	64.57 (0.28)	66.32 (0.26)	< 00001	0.027 (0.013 ─ 0.042)	< 00001
**AS**	63.47 (0.53)	64.37 (0.59)	61.91 (0.66)	63.79 (0.54)	0.0352	0.005 (0.000 ─ 0.012)	0.8075
**Protein**	0.90 (0.01)	0.92 (0.01)	0.86 (0.01)	0.86 (0.01)	< 00001	0.016 (0.006 ─ 0.029)	0.0003
**LA**	0.48 (0.01)	0.41 (0.01)	0.41 (0.02)	0.39 (0.02)	< 00001	0.016 (0.006 ─ 0.028)	0.0201
**ALA**	0.12 (0.01)	0.10 (0.01)	0.12 (0.01)	0.12 (0.01)	0.3060		
**DHA**	0.22 (0.01)	0.23 (0.02)	0.20 (0.01)	0.24 (0.02)	0.2644		
**EPA+DHA**	0.19 (0.01)	0.21 (0.01)	0.17 (0.01)	0.22 (0.02)	0.1921		
**Fiber**	0.32 (0.01)	0.35 (0.02)	0.37 (0.02)	0.41 (0.02)	0.0003	0.011 (0.003 ─ 0.021)	< 00001
**Vitamin A**	0.43 (0.01)	0.51 (0.02)	0.49 (0.02)	0.51 (0.02)	0.0002	0.012 (0.003 ─ 0.022)	0.0044
**Thiamin**	0.92 (0.00)	0.94 (0.00)	0.94 (0.00)	0.95 (0.00)	< 00001	0.019 (0.007 ─ 0.032)	< 00001
**Riboflavin**	0.76 (0.01)	0.78 (0.01)	0.74 (0.01)	0.74 (0.01)	0.0593		
**Niacin**	1.00 (0.00)	1.00 (0.00)	0.99 (0.00)	1.00 (0.00)	0.0183	0.006 (0.000 ─ 0.014)	0.0002
**Pantothenic acid**	0.89 (0.01)	0.88 (0.01)	0.85 (0.01)	0.88 (0.01)	0.0001	0.013 (0.004 ─ 0.024)	0.0021
**Vitamin B-6**	0.70 (0.01)	0.70 (0.01)	0.65 (0.02)	0.70 (0.01)	0.0641		
**Folate**	0.63 (0.01)	0.67 (0.02)	0.65 (0.02)	0.75 (0.01)	< 00001	0.018 (0.007 ─ 0.031)	0.0002
**Vitamin B-12**	0.68 (0.01)	0.71 (0.01)	0.60 (0.02)	0.60 (0.02)	< 00001	0.021 (0.009 ─ 0.035)	< 00001
**Vitamin C**	0.35 (0.02)	0.44 (0.02)	0.45 (0.02)	0.56 (0.02)	< 00001	0.040 (0.022 ─ 0.058)	< 00001
**Vitamin D**	0.04 (0.00)	0.03 (0.00)	0.03 (0.00)	0.03 (0.00)	0.7361		
**Vitamin E**	0.85 (0.01)	0.85 (0.01)	0.81 (0.01)	0.84 (0.01)	0.0049	0.007 (0.001 ─ 0.016)	0.8071
**Iodine**	0.71 (0.01)	0.72 (0.01)	0.68 (0.01)	0.72 (0.01)	0.1101		
**Magnesium**	0.87 (0.01)	0.88 (0.01)	0.86 (0.01)	0.88 (0.01)	0.1107		
**Phosphorus**	1.00 (0.00)	1.00 (0.00)	1.00 (0.00)	1.00 (0.00)	0.6774		
**Potassium**	0.66 (0.01)	0.66 (0.01)	0.61 (0.02)	0.66 (0.01)	0.0220	0.006 (0.000 ─ 0.013)	0.4565
**Selenium**	0.94 (0.01)	0.94 (0.01)	0.94 (0.01)	0.95 (0.01)	0.3633		
**Zinc**	0.46 (0.01)	0.44 (0.02)	0.36 (0.02)	0.26 (0.02)	< 00001	0.048 (0.030 ─ 0.068)	< 00001
**Copper**	0.88 (0.01)	0.88 (0.01)	0.86 (0.01)	0.88 (0.01)	0.3073		
**Manganese**	0.84 (0.01)	0.85 (0.01)	0.85 (0.01)	0.86 (0.01)	0.1162		
**Calcium**	0.65 (0.01)	0.66 (0.02)	0.62 (0.02)	0.64 (0.02)	0.3766		
**Iron**	0.84 (0.01)	0.83 (0.01)	0.78 (0.01)	0.80 (0.01)	0.0002	0.011 (0.003 ─ 0.022)	< 00001
**MS**	64.29 (0.46)	66.24 (0.48)	67.23 (0.47)	68.85 (0.47)	< 00001	0.028 (0.014 ─ 0.044)	< 00001
**Protein**	0.92 (0.01)	0.96 (0.00)	0.96 (0.00)	0.98 (0.00)	< 00001	0.041 (0.024 ─ 0.059)	< 00001
**Carbohydrates**	0.88 (0.01)	0.88 (0.01)	0.87 (0.01)	0.88 (0.01)	0.5520		
**Total fat**	0.85 (0.01)	0.86 (0.01)	0.86 (0.01)	0.85 (0.01)	0.6586		
**SFA**	0.32 (0.01)	0.32 (0.01)	0.33 (0.01)	0.33 (0.01)	0.9018		
**Sugars**	0.46 (0.02)	0.55 (0.02)	0.59 (0.02)	0.63 (0.02)	< 00001	0.036 (0.019 ─ 0.053)	< 00001
**Sodium**	0.43 (0.01)	0.40 (0.01)	0.42 (0.01)	0.46 (0.01)	0.0119	0.006 (0.000 ─ 0.015)	0.4962

Note. Data presented as mean (standard error). Q = quartile.

Supplementary Table 34. PANDiet score across quartiles of DI

	Q1	Q2	Q3	Q4	P	η2 (95%CI)	Trend
**PANDiet score**	64.36 (0.19)	65.37 (0.27)	64.89 (0.32)	65.62 (0.41)	0.0026	0.008 (0.001 ─ 0.017)	0.2313
**AS**	62.85 (0.43)	64.37 (0.54)	63.83 (0.67)	63.13 (1.05)	0.1688		
**Protein**	0.90 (0.01)	0.89 (0.01)	0.87 (0.01)	0.84 (0.02)	0.0010	0.010 (0.002 ─ 0.019)	0.0049
**LA**	0.43 (0.01)	0.40 (0.01)	0.43 (0.02)	0.56 (0.02)	< 00001	0.021(0.009 ─ 0.035)	0.0007
**ALA**	0.12 (0.01)	0.10 (0.01)	0.11 (0.01)	0.16 (0.02)	0.0119	0.006 (0.000 ─ 0.015)	0.0063
**DHA**	0.23 (0.01)	0.21 (0.01)	0.20 (0.02)	0.23 (0.02)	0.5341		
**EPA+DHA**	0.20 (0.01)	0.19 (0.01)	0.17 (0.02)	0.20 (0.02)	0.4717		
**Fiber**	0.31 (0.01)	0.39 (0.02)	0.39 (0.02)	0.43 (0.02)	< 00001	0.019 (0.007 ─ 0.032)	< 00001
**Vitamin A**	0.45 (0.01)	0.53 (0.02)	0.48 (0.02)	0.45 (0.02)	0.0014	0.009 (0.001 ─ 0.019)	0.4872
**Thiamin**	0.94 (0.00)	0.95 (0.00)	0.93 (0.01)	0.94 (0.01)	0.0253	0.005 (0.000 ─ 0.013)	0.0881
**Riboflavin**	0.76 (0.01)	0.77 (0.01)	0.76 (0.01)	0.70 (0.03)	0.0140	0.006 (0.000 ─ 0.014)	0.0922
**Niacin**	1.00 (0.00)	1.00 (0.00)	1.00 (0.00)	1.00 (0.00)	0.2225		
**Pantothenic acid**	0.88 (0.00)	0.88 (0.01)	0.87 (0.01)	0.83 (0.01)	0.0006	0.010 (0.019 ─ 0.020)	0.0067
**Vitamin B-6**	0.69 (0.01)	0.71 (0.01)	0.69 (0.02)	0.62 (0.02)	0.0196	0.006 (0.000 ─ 0.014)	0.0988
**Folate**	0.62 (0.01)	0.70 (0.01)	0.70 (0.02)	0.73 (0.02)	< 00001	0.002 (0.008 ─ 0.034)	0.0001
**Vitamin B-12**	0.67 (0.01)	0.68 (0.01)	0.65 (0.02)	0.56 (0.03)	0.0002	0.011 (0.003 ─ 0.022)	0.0052
**Vitamin C**	0.37 (0.01)	0.47 (0.02)	0.50 (0.02)	0.50 (0.03)	< 00001	0.023 (0.009 ─ 0.037)	< 00001
**Vitamin D**	0.04 (0.00)	0.03 (0.00)	0.03 (0.00)	0.03 (0.01)	0.6394		
**Vitamin E**	0.82 (0.01)	0.85 (0.01)	0.86 (0.01)	0.88 (0.01)	0.0003	0.011 (0.002 ─ 0.021)	< 00001
**Iodine**	0.71 (0.01)	0.72 (0.01)	0.71 (0.02)	0.69 (0.02)	0.6663		
**Magnesium**	0.87 (0.01)	0.89 (0.01)	0.88 (0.01)	0.88 (0.01)	0.1292		
**Phosphorus**	1.00 (0.00)	1.00 (0.00)	1.00 (0.00)	1.00 (0.00)	0.6323		
**Potassium**	0.63 (0.01)	0.67 (0.01)	0.68 (0.02)	0.64 (0.02)	0.0646		
**Selenium**	0.94 (0.00)	0.96 (0.00)	0.92 (0.01)	0.94 (0.01)	0.0074	0.007 (0.001 ─ 0.015)	0.1220
**Zinc**	0.44 (0.01)	0.39 (0.02)	0.38 (0.02)	0.23 (0.02)	< 00001	0.033 (0.018 ─ 0.051)	< 00001
**Copper**	0.87 (0.00)	0.88 (0.01)	0.88 (0.01)	0.88 (0.01)	0.1535		
**Manganese**	0.83 (0.01)	0.86 (0.01)	0.86 (0.01)	0.89 (0.01)	< 00001	0.014 (0.004 ─ 0.026)	< 00001
**Calcium**	0.64 (0.01)	0.64 (0.02)	0.65 (0.02)	0.66 (0.03)	0.8932		
**Iron**	0.82 (0.01)	0.83 (0.01)	0.81 (0.01)	0.79 (0.02)	0.2135		
**MS**	65.87 (0.36)	66.36 (0.46)	65.95 (0.57)	68.11 (0.74)	0.0635		
**Protein**	0.94 (0.00)	0.96 (0.00)	0.95 (0.01)	0.97 (0.01)	< 00001	0.013 (0.004 ─ 0.025)	0.6574
**Carbohydrates**	0.88 (0.01)	0.88 (0.01)	0.86 (0.01)	0.87 (0.01)	0.1112		
**Total fat**	0.86 (0.01)	0.86 (0.01)	0.83 (0.01)	0.84 (0.01)	0.1458		
**SFA**	0.32 (0.01)	0.30 (0.01)	0.35 (0.02)	0.41 (0.02)	< 00001	0.016 (0.005 ─ 0.029)	0.0032
**Sugars**	0.53 (0.01)	0.55 (0.02)	0.56 (0.02)	0.55 (0.03)	0.4897		
**Sodium**	0.43 (0.01)	0.43 (0.01)	0.40 (0.01)	0.45 (0.02)	0.2818		

Note. Data presented as mean (standard error). Q = quartile.

**Supplementary Table 35. Environmental impact across quintiles of WISH**								
	**Q1**	**Q2**	**Q3**	**Q4**	**Q5**	**P**	**η2 (95%CI)**	**Trend**
**Product Environmental Footprint**	0.75 (0.01)	0.72 (0.02)	0.77 (0.02)	0.71 (0.01)	0.68 (0.01)	< 00001	0.015 (0.005 — 0.027)	0.0098
**Greenhouse gas emission**	5.93 (0.11)	5.59 (0.13)	6.02 (0.15)	5.36 (0.10)	5.01 (0.10)	< 00001	0.026 (0.012 — 0.041)	< 0.0001
**Ozone depletion**	0.73 (0.02)	0.69 (0.02)	0.70 (0.02)	0.67 (0.01)	0.65 (0.01)	0.0165	0.007 (0.000 — 0.015)	0.0063
**Ionizing radiation**	1.24 (0.02)	1.17 (0.02)	1.23 (0.02)	1.15 (0.02)	1.11 (0.02)	< 00001	0.018 (0.006 — 0.031)	0.0001
**Photochemical ozone formation**	17.33 (0.54)	17.26 (0.58)	19.42 (0.58)	18.72 (0.54)	17.81 (0.49)	0.0148	0.007 (0.000 — 0.015)	< 0.0001
**Particulate matter emissions**	0.58 (0.01)	0.55 (0.01)	0.60 (0.01)	0.54 (0.01)	0.50 (0.01)	< 00001	0.020 (0.008 — 0.033)	0.0014
**Acidification**	0.08 (0.00)	0.07 (0.00)	0.08 (0.00)	0.07 (0.00)	0.07 (0.00)	< 00001	0.022 (0.009 — 0.035)	0.0003
**Terrestrial eutrophication**	25.74 (0.48)	24.86 (0.57)	27.31 (0.66)	25.17 (0.51)	23.79 (0.47)	0.0002	0.013 (0.003 — 0.023)	0.4082
**Freshwater eutrophication**	0.33 (0.01)	0.31 (0.01)	0.33 (0.01)	0.29 (0.01)	0.27 (0.01)	< 00001	0.026 (0.012 — 0.041)	< 0.0001
**Marine eutrophication**	0.83 (0.01)	0.79 (0.02)	0.81 (0.01)	0.78 (0.02)	0.76 (0.02)	0.0082	0.008 (0.001 — 0.016)	< 0.0001
**Land use**	80.79 (1.46)	74.52 (1.52)	77.04 (1.41)	73.27 (1.19)	70.60 (1.42)	< 00001	0.018 (0.006 — 0.030)	< 0.0001
**Freshwater ecotoxicity**	348.88 (8.18)	332.04 (9.58)	355.03 (9.86)	315.68 (7.31)	296.69 (8.34)	< 00001	0.017 (0.005 — 0.029)	< 0.0001
**Water use**	7.20 (0.21)	7.76 (0.25)	7.99 (0.22)	8.25 (0.21)	9.13 (0.27)	< 00001	0.021 (0.008 — 0.034)	< 0.0001
**Fossils resource**	57.82 (0.96)	55.09 (0.92)	58.86 (0.96)	55.84 (0.77)	54.08 (0.82)	0.0007	0.011 (0.002 — 0.021)	0.2178
**Metals and minerals resource**	31.67 (0.65)	30.35 (0.70)	31.67 (0.68)	29.79 (0.52)	28.94 (0.54)	0.0067	0.008 (0.001 — 0.017)	0.0012
**Ratio PANDiet/PEF**	0.90 (0.01)	0.99 (0.02)	0.97 (0.02)	1.00 (0.02)	1.11 (0.02)	< 00001	0.040 (0.023 — 0.058)	< 0.0001

Note. Data presented as mean (standard error). Q = quintile.

Supplementary Table 36. Environmental impact across quintiles of PHDI								
	**Q1**	**Q2**	**Q3**	**Q4**	**Q5**	**p**	**η2 (95%CI)**	**Trend**
**Product Environmental Footprint**	0.75 (0.02)	0.74 (0.01)	0.71 (0.01)	0.70 (0.01)	0.75 (0.01)	0.0361	0.006 (0.000 — 0.013)	0.4293
**Greenhouse gas emission**	5.95 (0.14)	5.62 (0.12)	5.40 (0.10)	5.30 (0.11)	5.77 (0.13)	0.0006	0.011 (0.002 — 0.021)	0.0408
**Ozone depletion**	0.72 (0.02)	0.72 (0.02)	0.71 (0.02)	0.63 (0.01)	0.66 (0.02)	0.0002	0.013 (0.003 — 0.023)	0.0007
**Ionizing radiation**	1.23 (0.02)	1.18 (0.02)	1.16 (0.02)	1.13 (0.02)	1.20 (0.02)	0.0012	0.011 (0.002 — 0.020)	0.0365
**Photochemical ozone formation**	17.80 (0.60)	19.53 (0.58)	18.39 (0.59)	17.16 (0.47)	17.36 (0.43)	0.0158	0.007 (0.000 — 0.015)	0.8221
**Particulate matter emissions**	0.58 (0.01)	0.57 (0.01)	0.54 (0.01)	0.52 (0.01)	0.57 (0.01)	0.0061	0.008 (0.001 — 0.017)	0.0409
**Acidification**	0.08 (0.00)	0.08 (0.00)	0.07 (0.00)	0.07 (0.00)	0.08 (0.00)	0.0045	0.009 (0.001 — 0.018)	0.0233
**Terrestrial eutrophication**	26.61 (0.69)	25.44 (0.49)	25.30 (0.52)	24.05 (0.46)	25.68 (0.52)	0.0220	0.007 (0.000 — 0.014)	0.2968
**Freshwater eutrophication**	0.32 (0.01)	0.31 (0.01)	0.29 (0.01)	0.29 (0.01)	0.31 (0.01)	0.0016	0.010 (0.002 — 0.019)	0.0159
**Marine eutrophication**	0.83 (0.02)	0.79 (0.01)	0.78 (0.01)	0.76 (0.01)	0.82 (0.02)	0.0108	0.008 (0.000 — 0.016)	0.0626
**Land use**	76.51 (1.65)	75.26 (1.32)	75.43 (1.28)	73.09 (1.36)	77.90 (1.44)	0.2290		
**Freshwater ecotoxicity**	345.14 (9.50)	322.32 (8.82)	320.73 (7.74)	318.64 (8.31)	354.03 (9.41)	0.0119	0.008 (0.000 — 0.016)	0.9026
**Water use**	6.77 (0.22)	8.19 (0.25)	8.18 (0.25)	8.54 (0.23)	8.71 (0.21)	< 0.0001	0.026 (0.012 — 0.041)	< 0.0001
**Fossils resource**	57.63 (1.11)	57.26 (0.81)	55.98 (0.83)	54.13 (0.80)	57.18 (0.85)	0.0429	0.006 (0.000 — 0.013)	0.2954
**Metals and minerals resource**	31.06 (0.69)	31.20 (0.64)	30.60 (0.64)	28.96 (0.56)	30.99 (0.59)	0.0863		
**Ratio PANDiet/PEF**	0.97 (0.02)	0.97 (0.02)	1.00 (0.02)	1.03 (0.02)	1.00 (0.02)	0.1236		

Note. Data presented as mean (standard error). Q = quintile.

Supplementary Table 37. Environmental impact across quintiles of ELD-I								
	**Q1**	**Q2**	**Q3**	**Q4**	**Q5**	**p**	**η2 (95%CI)**	**Trend**
**Product Environmental Footprint**	0.84 (0.02)	0.74 (0.01)	0.72 (0.01)	0.70 (0.01)	0.62 (0.01)	< 00001	0.078 (0.054 — 0.101)	< 00001
**Greenhouse gas emission**	6.82 (0.16)	5.69 (0.10)	5.52 (0.10)	5.15 (0.09)	4.51 (0.08)	< 00001	0.118 (0.089 — 0.144)	< 00001
**Ozone depletion**	0.75 (0.02)	0.70 (0.02)	0.67 (0.02)	0.70 (0.02)	0.62 (0.02)	< 00001	0.016 (0.005 — 0.028)	< 00001
**Ionizing radiation**	1.24 (0.02)	1.24 (0.02)	1.17 (0.02)	1.17 (0.02)	1.07 (0.02)	< 00001	0.028 (0.013 — 0.043)	< 00001
**Photochemical ozone formation**	20.12 (0.61)	17.57 (0.50)	18.14 (0.60)	18.49 (0.48)	15.53 (0.48)	< 00001	0.021 (0.009 — 0.035)	0.0045
**Particulate matter emissions**	0.68 (0.02)	0.56 (0.01)	0.55 (0.01)	0.51 (0.01)	0.44 (0.01)	< 00001	0.114 (0.086 — 0.140)	< 00001
**Acidification**	0.09 (0.00)	0.08 (0.00)	0.07 (0.00)	0.07 (0.00)	0.06 (0.00)	< 00001	0.128 (0.099 — 0.156)	< 00001
**Terrestrial eutrophication**	29.13 (0.65)	26.21 (0.60)	25.10 (0.46)	23.84 (0.41)	21.67 (0.45)	< 00001	0.061 (0.040 — 0.083)	< 00001
**Freshwater eutrophication**	0.39 (0.01)	0.31 (0.01)	0.30 (0.01)	0.27 (0.01)	0.23 (0.00)	< 00001	0.140 (0.110 — 0.169)	< 00001
**Marine eutrophication**	0.86 (0.02)	0.82 (0.02)	0.79 (0.01)	0.78 (0.01)	0.72 (0.01)	< 00001	0.028 (0.013 — 0.043)	< 00001
**Land use**	79.86 (1.57)	77.62 (1.46)	75.35 (1.34)	74.06 (1.28)	69.39 (1.33)	< 00001	0.018 (0.006 — 0.030)	< 00001
**Freshwater ecotoxicity**	412.73 (11.74)	334.72 (7.14)	325.55 (7.27)	301.57 (7.68)	256.90 (5.49)	< 00001	0.102 (0.075 — 0.127)	< 00001
**Water use**	6.89 (0.23)	7.67 (0.22)	7.86 (0.20)	9.20 (0.28)	8.86 (0.22)	< 00001	0.037 (0.020 — 0.054)	< 00001
**Fossils resource**	59.49 (1.02)	57.47 (0.94)	56.05 (0.84)	56.70 (0.84)	51.55 (0.72)	< 00001	0.024 (0.010 — 0.038)	< 00001
**Metals and minerals resource**	32.74 (0.70)	31.22 (0.70)	29.80 (0.56)	31.10 (0.59)	27.31 (0.50)	< 00001	0.024 (0.010 — 0.038)	< 00001
**Ratio PANDiet/PEF**	0.86 (0.02)	0.95 (0.02)	0.99 (0.02)	1.03 (0.02)	1.16 (0.02)	< 00001	0.088 (0.062 — 0.112)	< 00001

Note. Data presented as mean (standard error). Q = quintile.

Supplementary Table 38. Environmental impact across quintiles of HRD

	Q1	Q2	Q3	Q4	Q5	p	η2 (95%CI)	Trend
**Product Environmental Footprint**	0.75 (0.01)	0.71 (0.01)	0.76 (0.02)	0.73 (0.01)	0.69 (0.01)	0.0073	0.008 (0.001-0.017)	0.1185
**Greenhouse gas emission**	5.92 (0.12)	5.53 (0.12)	5.91 (0.14)	5.50 (0.11)	5.15 (0.11)	< 00001	0.015 (0.004-0.026)	0.0001
**Ozone depletion**	0.72 (0.02)	0.67 (0.02)	0.73 (0.02)	0.69 (0.02)	0.65 (0.02)	0.0025	0.010 (0.001-0.019)	0.2817
**Ionizing radiation**	1.23 (0.02)	1.17 (0.02)	1.19 (0.02)	1.19 (0.02)	1.12 (0.02)	0.0044	0.009 (0.001-0.018)	0.0121
**Photochemical ozone formation**	16.60 (0.52)	17.16 (0.49)	19.32 (0.67)	19.41 (0.56)	18.32 (0.47)	0.0002	0.013 (0.003-0.023)	< 00001
**Particulate matter emissions**	0.58 (0.01)	0.54 (0.01)	0.59 (0.01)	0.55 (0.01)	0.52 (0.01)	0.001	0.011 (0.002-0.020)	0.0334
**Acidification**	0.08 (0.00)	0.07 (0.00)	0.08 (0.00)	0.07 (0.00)	0.07 (0.00)	0.0002	0.013 (0.003-0.023)	0.0087
**Terrestrial eutrophication**	26.68 (0.66)	24.38 (0.47)	26.41 (0.58)	25.42 (0.50)	24.33 (0.50)	0.0027	0.009 (0.001-0.019)	0.7963
**Freshwater eutrophication**	0.33 (0.01)	0.30 (0.01)	0.32 (0.01)	0.30 (0.01)	0.28 (0.01)	< 00001	0.016 (0.005-0.028)	0.0001
**Marine eutrophication**	0.82 (0.02)	0.78 (0.01)	0.80 (0.01)	0.82 (0.02)	0.77 (0.02)	0.0674		
**Land use**	79.20 (1.65)	74.95 (1.30)	75.99 (1.35)	76.18 (1.34)	70.95 (1.41)	0.0028	0.009 (0.001-0.018)	0.0356
**Freshwater ecotoxicity**	352.81 (8.92)	324.18 (8.03)	355.36 (9.78)	314.04 (8.26)	307.89 (8.78)	0.0001	0.014 (0.004-0.025)	0.0001
**Water use**	7.19 (0.24)	7.82 (0.22)	7.88 (0.21)	8.57 (0.25)	8.80 (0.26)	< 00001	0.017 (0.005-0.028)	< 00001
**Fossils resource**	57.11 (1.01)	55.36 (0.90)	57.35 (0.87)	57.67 (0.87)	54.95 (0.82)	0.1197		
**Metals and minerals resource**	31.35 (0.65)	29.96 (0.64)	31.61 (0.66)	30.60 (0.59)	29.31 (0.59)	0.0679		
**Ratio PANDiet/PEF**	0.94 (0.02)	0.99 (0.02)	0.96 (0.02)	0.99 (0.02)	1.09 (0.02)	< 00001	0.022 (0.009-0.035)	< 00001

Note. Data presented as mean (standard error). Q = quintile.

Supplementary Table 39. Environmental impact across quintiles of ELI

	Q1	Q2	Q3	Q4	Q5	p	η2 (95%CI)	Trend
**Product Environmental Footprint**	0.78 (0.01)	0.75 (0.01)	0.74 (0.01)	0.70 (0.01)	0.62 (0.01)	< 0.0001	0.037 (0.020 ─ 0.054)	< 0.0001
**Greenhouse gas emission**	6.21 (0.11)	5.84 (0.12)	5.69 (0.12)	5.15 (0.12)	4.45 (0.09)	< 0.0001	0.064 (0.042 ─ 0.085)	< 0.0001
**Ozone depletion**	0.73 (0.02)	0.70 (0.01)	0.67 (0.02)	0.71 (0.02)	0.62 (0.02)	0.0004	0.012 (0.003 ─ 0.022)	0.0010
**Ionizing radiation**	1.28 (0.02)	1.18 (0.02)	1.22 (0.02)	1.11 (0.02)	1.03 (0.02)	< 0.0001	0.054 (0.034 ─ 0.075)	< 0.0001
**Photochemical ozone formation**	16.34 (0.49)	18.31 (0.49)	18.51 (0.57)	19.48 (0.64)	19.04 (0.58)	0.0004	0.012 (0.002 ─ 0.022)	< 0.0001
**Particulate matter emissions**	0.60 (0.01)	0.58 (0.01)	0.56 (0.01)	0.52 (0.01)	0.46 (0.01)	< 0.0001	0.039 (0.022 ─ 0.057)	< 0.0001
**Acidification**	0.08 (0.00)	0.08 (0.00)	0.08 (0.00)	0.07 (0.00)	0.06 (0.00)	< 0.0001	0.045 (0.026 ─ 0.063)	< 0.0001
**Terrestrial eutrophication**	26.50 (0.46)	26.54 (0.58)	25.58 (0.56)	24.01 (0.52)	22.65 (0.46)	< 0.0001	0.019 (0.007 ─ 0.032)	< 0.0001
**Freshwater eutrophication**	0.34 (0.01)	0.32 (0.01)	0.31 (0.01)	0.28 (0.01)	0.23 (0.01)	< 0.0001	0.065 (0.043 ─ 0.087)	< 0.0001
**Marine eutrophication**	0.85 (0.01)	0.80 (0.01)	0.81 (0.02)	0.76 (0.02)	0.71 (0.02)	< 0.0001	0.025 (0.011 ─ 0.040)	< 0.0001
**Land use**	82.10 (1.40)	75.79 (1.23)	77.16 (1.50)	71.54 (1.51)	65.39 (1.26)	< 0.0001	0.040 (0.023 ─ 0.058)	< 0.0001
**Freshwater ecotoxicity**	373.42 (7.38)	346.84 (8.76)	342.20 (9.55)	295.19 (8.55)	247.55 (6.47)	< 0.0001	0.064 (0.042 ─ 0.085)	< 0.0001
**Water use**	7.21 (0.21)	7.59 (0.20)	8.51 (0.27)	9.44 (0.29)	8.28 (0.23)	< 0.0001	0.028 (0.013 ─ 0.043)	< 0.0001
**Fossils resource**	58.55 (0.92)	56.72 (0.80)	58.03 (0.93)	55.19 (0.92)	51.57 (0.78)	< 0.0001	0.019 (0.007 ─ 0.031)	< 0.0001
**Metals and minerals resource**	31.91 (0.59)	30.88 (0.54)	30.98 (0.75)	30.32 (0.68)	27.41 (0.56)	0.0001	0.014 (0.004 ─ 0.025)	< 0.0001
**Ratio PANDiet/PEF**	0.88 (0.01)	0.97 (0.02)	0.99 (0.02)	1.03 (0.02)	1.18 (0.02)	< 0.0001	0.076 (0.053 ─ 0.100)	< 0.0001

Note. Data presented as mean (standard error). Q = quintile.

Supplementary Table 40. Environmental impact across quintiles of SHDI

	Q1	Q2	Q3	Q4	Q5	p	η2 (95%CI)	Trend
**Product Environmental Footprint**	0.70 (0.01)	0.75 (0.01)	0.75 (0.02)	0.71 (0.01)	0.75 (0.01)	0.0047	0.009 (0.001 ─ 0.017)	0.0337
**Greenhouse gas emission**	5.43 (0.11)	5.86 (0.12)	5.78 (0.15)	5.36 (0.12)	5.60 (0.11)	0.0106	0.008 (0.001 ─ 0.016)	0.7852
**Ozone depletion**	0.68 (0.02)	0.68 (0.02)	0.71 (0.02)	0.68 (0.02)	0.70 (0.01)	0.5752		
**Ionizing radiation**	1.15 (0.02)	1.23 (0.02)	1.20 (0.02)	1.15 (0.02)	1.19 (0.02)	0.011	0.008 (0.000 ─ 0.016)	0.2749
**Photochemical ozone formation**	14.92 (0.41)	17.38 (0.42)	20.27 (0.69)	19.18 (0.68)	21.15 (0.58)	< 0.0001	0.051 (0.031 ─ 0.071)	< 0.0001
**Particulate matter emissions**	0.53 (0.01)	0.57 (0.01)	0.58 (0.02)	0.53 (0.01)	0.57 (0.01)	0.0139	0.007 (0.000 ─ 0.015)	0.1807
**Acidification**	0.07 (0.00)	0.08 (0.00)	0.08 (0.00)	0.07 (0.00)	0.08 (0.00)	0.0163	0.007 (0.000 ─ 0.015)	0.5213
**Terrestrial eutrophication**	23.90 (0.46)	26.30 (0.55)	26.17 (0.60)	24.58 (0.53)	26.77 (0.58)	0.0003	0.012 (0.003 ─ 0.023)	0.0004
**Freshwater eutrophication**	0.30 (0.01)	0.32 (0.01)	0.31 (0.01)	0.28 (0.01)	0.30 (0.01)	0.0145	0.007 (0.000 ─ 0.015)	0.2484
**Marine eutrophication**	0.77 (0.01)	0.80 (0.01)	0.81 (0.02)	0.79 (0.02)	0.82 (0.02)	0.2049		
**Land use**	76.20 (1.53)	76.27 (1.23)	74.86 (1.35)	73.99 (1.47)	76.17 (1.44)	0.7744		
**Freshwater ecotoxicity**	327.18 (7.77)	348.79 (8.29)	332.69 (10.26)	304.46 (7.84)	336.68 (9.43)	0.0169	0.007 (0.000 ─ 0.015)	0.1425
**Water use**	6.98 (0.21)	7.97 (0.21)	8.34 (0.24)	8.82 (0.28)	8.71 (0.25)	< 0.0001	0.025 (0.011 ─ 0.039)	< 0.0001
**Fossils resource**	53.18 (0.91)	57.25 (0.81)	58.02 (0.92)	56.37 (0.94)	59.29 (0.85)	< 0.0001	0.017 (0.010 ─ 0.029)	< 0.0001
**Metals and minerals resource**	29.73 (0.61)	30.59 (0.54)	30.91 (0.63)	30.80 (0.75)	31.56 (0.66)	0.3351		
**Ratio PANDiet/PEF**	0.99 (0.02)	0.96 (0.01)	0.98 (0.02)	1.03 (0.02)	1.00 (0.02)	0.1399		

Note. Data presented as mean (standard error). Q = quintile.

Supplementary Table 41. Environmental impact across quartiles of HSDI							
	**Q1**	**Q2**	**Q3**	**Q4**	**p**	**η2 (95%CI)**	**Trend**
**Product Environmental Footprint**	0.75 (0.01)	0.74 (0.01)	0.71 (0.01)	0.69 (0.01)	0.0052	0.007 (0.001 ─ 0.016)	< 0.0001
**Greenhouse gas emission**	5.78 (0.08)	5.75 (0.12)	5.39 (0.12)	5.25 (0.13)	0.0016	0.009 (0.001 ─ 0.018)	< 0.0001
**Ozone depletion**	0.72 (0.01)	0.71 (0.02)	0.67 (0.02)	0.60 (0.02)	< 0.0001	0.018 (0.006 ─ 0.030)	< 0.0001
**Ionizing radiation**	1.20 (0.01)	1.19 (0.02)	1.17 (0.02)	1.14 (0.02)	0.1002		
**Photochemical ozone formation**	19.57 (0.42)	18.29 (0.51)	16.87 (0.56)	15.46 (0.41)	< 0.0001	0.022 (0.009 ─ 0.036)	< 0.0001
**Particulate matter emissions**	0.58 (0.01)	0.56 (0.01)	0.54 (0.01)	0.52 (0.01)	0.0007	0.010 (0.002 ─ 0.019)	< 0.0001
**Acidification**	0.08 (0.00)	0.08 (0.00)	0.07 (0.00)	0.07 (0.00)	0.0004	0.011 (0.002 ─ 0.021)	< 0.0001
**Terrestrial eutrophication**	26.60 (0.44)	25.51 (0.46)	24.29 (0.50)	23.85 (0.53)	0.0002	0.011 (0.003 ─ 0.022)	< 0.0001
**Freshwater eutrophication**	0.32 (0.01)	0.31 (0.01)	0.30 (0.01)	0.29 (0.01)	0.0090	0.007 (0.001 ─ 0.015)	< 0.0001
**Marine eutrophication**	0.82 (0.01)	0.79 (0.01)	0.79 (0.02)	0.77 (0.02)	0.0244	0.005 (0.000 ─ 0.013)	< 0.0001
**Land use**	76.59 (0.99)	75.39 (1.17)	73.69 (1.55)	75.69 (1.72)	0.4707		
**Freshwater ecotoxicity**	333.64 (6.07)	339.47 (8.28)	323.42 (8.59)	324.19 (10.04)	0.4794		
**Water use**	7.41 (0.16)	8.67 (0.21)	8.10 (0.24)	8.30 (0.27)	< 0.0001	0.014 (0.004 ─ 0.026)	< 0.0001
**Fossils resource**	57.94 (0.63)	57.01 (0.81)	54.92 (0.98)	53.79 (0.92)	0.0014	0.009 (0.002 ─ 0.019)	< 0.0001
**Metals and minerals resource**	31.41 (0.48)	31.26 (0.54)	29.36 (0.60)	28.85 (0.64)	0.0025	0.008 (0.001 ─ 0.018)	< 0.0001
**Ratio PANDiet/PEF**	0.94 (0.01)	0.98 (0.02)	1.03 (0.02)	1.08 (0.02)	< 0.0001	0.026 (0.012 ─ 0.041)	< 0.0001

Note. Data presented as mean (standard error). Q = quartile.

Supplementary Table 42. Environmental impact across quartiles of ELDS

	Q1	Q2	Q3	Q4	p	η2 (95%CI)	Trend
**Product Environmental Footprint**	0.80 (0.01)	0.75 (0.01)	0.67 (0.01)	0.64 (0.01)	< 0.0001	0.062 (0.041 ─ 0.084)	< 0.0001
**Greenhouse gas emission**	6.30 (0.11)	5.77 (0.11)	5.05 (0.09)	4.76 (0.09)	< 0.0001	0.074 (0.051 ─ 0.097)	< 0.0001
**Ozone depletion**	0.74 (0.02)	0.72 (0.02)	0.65 (0.01)	0.58 (0.01)	< 0.0001	0.035 (0.019 ─ 0.052)	< 0.0001
**Ionizing radiation**	1.25 (0.02)	1.22 (0.02)	1.12 (0.02)	1.07 (0.02)	< 0.0001	0.042 (0.025 ─ 0.061)	< 0.0001
**Photochemical ozone formation**	19.94 (0.49)	18.80 (0.52)	16.39 (0.45)	15.54 (0.38)	< 0.0001	0.030 (0.015 ─ 0.047)	< 0.0001
**Particulate matter emissions**	0.63 (0.01)	0.58 (0.01)	0.50 (0.01)	0.46 (0.01)	< 0.0001	0.071 (0.049 ─ 0.094)	< 0.0001
**Acidification**	0.09 (0.00)	0.08 (0.00)	0.07 (0.00)	0.06 (0.00)	< 0.0001	0.073 (0.050 ─ 0.096)	< 0.0001
**Terrestrial eutrophication**	28.26 (0.52)	25.77 (0.48)	22.99 (0.38)	22.49 (0.42)	< 0.0001	0.055 (0.035 ─ 0.076)	< 0.0001
**Freshwater eutrophication**	0.35 (0.01)	0.31 (0.01)	0.27 (0.01)	0.25 (0.01)	< 0.0001	0.071 (0.049 ─ 0.094)	< 0.0001
**Marine eutrophication**	0.86 (0.01)	0.81 (0.01)	0.75 (0.01)	0.73 (0.01)	< 0.0001	0.034 (0.019 ─ 0.052)	< 0.0001
**Land use**	81.28 (1.22)	76.71 (1.22)	70.04 (1.18)	69.63 (1.32)	< 0.0001	0.035 (0.019 ─ 0.053)	< 0.0001
**Freshwater ecotoxicity**	370.39 (7.71)	337.17 (7.97)	300.11 (6.64)	286.67 (7.83)	< 0.0001	0.042 (0.024 ─ 0.060)	< 0.0001
**Water use**	7.67 (0.19)	8.18 (0.24)	7.96 (0.18)	8.47 (0.24)	0.0492	0.005 (0.000 ─ 0.012)	0.0002
**Fossils resource**	60.23 (0.81)	58.24 (0.82)	52.83 (0.68)	51.12 (0.70)	< 0.0001	0.049 (0.031 ─ 0.069)	< 0.0001
**Metals and minerals resource**	32.63 (0.56)	32.00 (0.60)	28.48 (0.48)	27.20 (0.49)	< 0.0001	0.037 (0.020 ─ 0.054)	< 0.0001
**Ratio PANDiet/PEF**	0.88 (0.01)	0.97 (0.01)	1.06 (0.02)	1.14 (0.02)	< 0.0001	0.089 (0.064 ─ 0.113)	< 0.0001

Note. Data presented as mean (standard error). Q = quartile.

Supplementary Table 43. Environmental impact across quartiles of DI							
	**Q1**	**Q2**	**Q3**	**Q4**	**p**	**η2 (95%CI)**	**Trend**
**Product Environmental Footprint**	0.73 (0.01)	0.70 (0.01)	0.68 (0.01)	0.66 (0.02)	< 0.0001	0.014 (0.004 ─ 0.026)	< 0.0001
**Greenhouse gas emission**	5.61 (0.07)	5.33 (0.10)	5.06 (0.12)	4.83 (0.15)	< 0.0001	0.022 (0.009 ─ 0.036)	< 0.0001
**Ozone depletion**	0.68 (0.01)	0.65 (0.01)	0.64 (0.02)	0.64 (0.02)	0.0125	0.006 (0.000 ─ 0.014)	0.0061
**Ionizing radiation**	1.19 (0.01)	1.15 (0.02)	1.13 (0.02)	1.11 (0.03)	0.0002	0.011 (0.003 ─ 0.022)	0.0009
**Photochemical ozone formation**	18.43 (0.37)	17.13 (0.42)	17.28 (0.57)	16.88 (0.77)	0.0017	0.009 (0.001 ─ 0.018)	0.0170
**Particulate matter emissions**	0.56 (0.01)	0.53 (0.01)	0.50 (0.01)	0.48 (0.02)	< 0.0001	0.021 (0.009 ─ 0.035)	< 0.0001
**Acidification**	0.08 (0.00)	0.07 (0.00)	0.07 (0.00)	0.06 (0.00)	< 0.0001	0.023 (0.009 ─ 0.037)	< 0.0001
**Terrestrial eutrophication**	25.50 (0.37)	24.76 (0.44)	23.73 (0.53)	23.18 (0.69)	< 0.0001	0.013 (0.004 ─ 0.024)	0.0003
**Freshwater eutrophication**	0.31 (0.00)	0.29 (0.01)	0.27 (0.01)	0.26 (0.01)	< 0.0001	0.023 (0.010 ─ 0.038)	< 0.0001
**Marine eutrophication**	0.80 (0.01)	0.80 (0.02)	0.75 (0.02)	0.75 (0.02)	0.0202	0.006 (0.000 ─ 0.013)	0.0009
**Land use**	75.92 (0.93)	74.46 (1.17)	71.65 (1.38)	70.35 (1.87)	0.0553		
**Freshwater ecotoxicity**	330.44 (5.24)	319.39 (7.30)	301.41 (8.81)	290.57 (11.79)	0.0017	0.009 (0.013 ─ 0.018)	< 0.0001
**Water use**	7.79 (0.15)	8.29 (0.21)	8.41 (0.26)	8.83 (0.33)	0.0008	0.010 (0.002 ─ 0.019)	0.0004
**Fossils resource**	56.78 (0.60)	54.78 (0.71)	53.72 (0.89)	53.15 (1.20)	0.0003	0.011 (0.002 ─ 0.021)	0.0014
**Metals and minerals resource**	30.46 (0.42)	29.70 (0.59)	28.76 (0.65)	28.70 (0.87)	0.0900		
**Ratio PANDiet/PEF**	0.98 (0.01)	1.03 (0.02)	1.07 (0.02)	1.12 (0.03)	< 0.0001	0.025 (0.011 ─ 0.040)	< 0.0001

Note. Data presented as mean (standard error). Q = quartile.

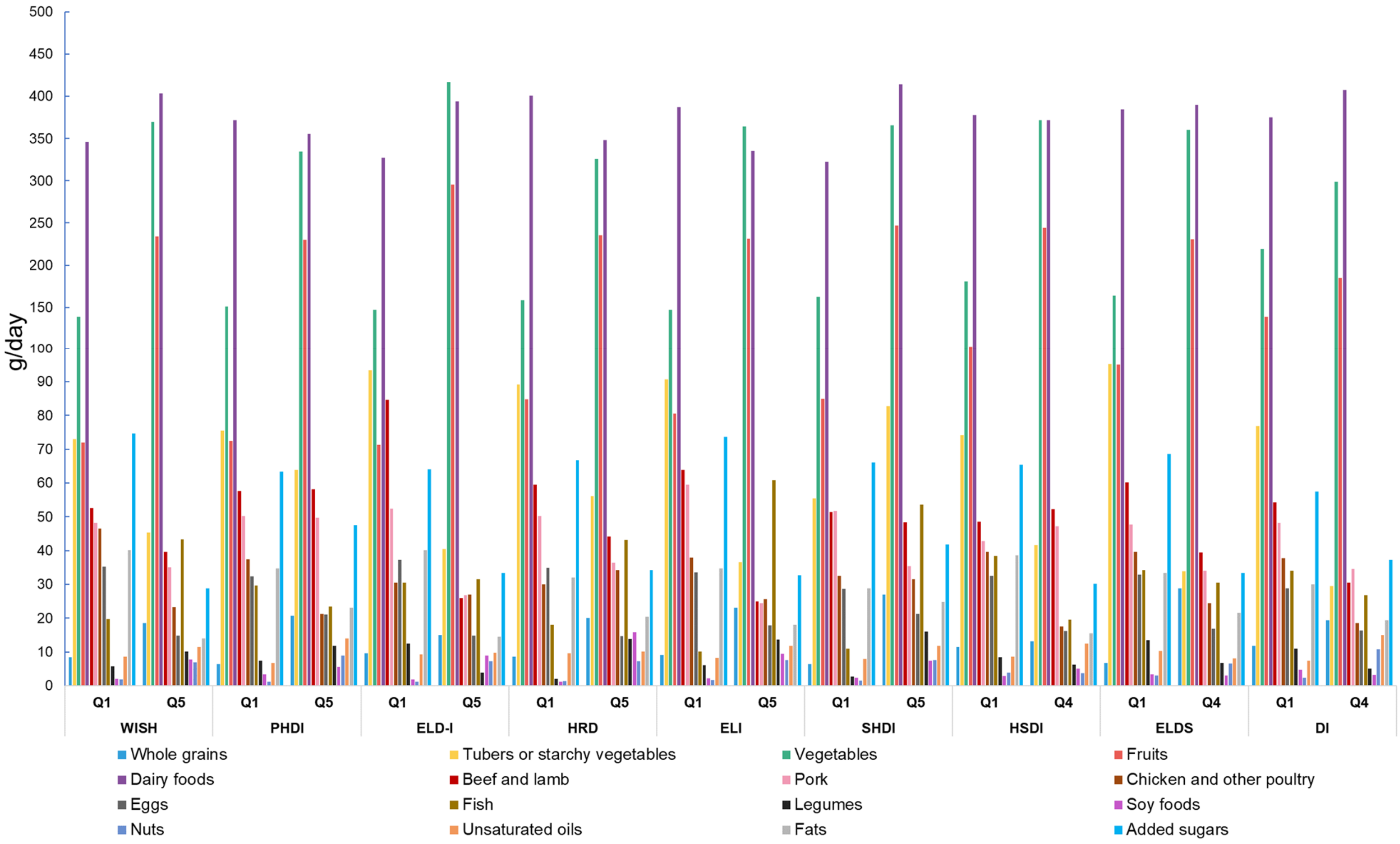

Supplementary Figure 5. Mean daily intake (g/day) of Eat-Lancet food components for participants in the highest and lowest quantiles of each dietary index. The indices HSDI, ELDS, and DI are categorized into quartiles, while the remaining indices are categorized into quintiles.
